# Transcriptome Analysis of Artificial Hybrid Pufferfish *Jiyan-1* and Its Parental Species: Implications for Pufferfish Heterosis

**DOI:** 10.1371/journal.pone.0058453

**Published:** 2013-03-08

**Authors:** Yang Gao, Huan Zhang, Qiang Gao, Lingling Wang, Fuchong Zhang, Vinu S. Siva, Zhi Zhou, Linsheng Song, Shicui Zhang

**Affiliations:** 1 Key Laboratory of Experimental Marine Biology, Institute of Oceanology, Chinese Academy of Sciences, Qingdao, China; 2 University of Chinese Academy of Sciences, Beijing, China; 3 Ocean and Fishery Sciences Research Institute of Hebei Province, Qinhuangdao, China; 4 Ocean University of China, Qingdao, China; Temasek Life Sciences Laboratory, Singapore

## Abstract

*Jiyan-1* puffer, the F_1_ hybrid of *Takifugu rubripes* and *Takifugu flavidus*, displays obvious heterosis in the growth performance, flavor and stress tolerance. In the present study, comparative analysis for the transcriptomes of *T. rubripes*, *T. flavidus* and *Jiyan-1* was performed aiming to reveal the possible mechanisms of heterosis in pufferfish. Whole transcriptomes were sequenced using the SOLiD4 platform, and a total of 44,305 transcripts corresponding to 18,164 genes were identified collectively. A total of 14,148 transcripts were differentially expressed. By comparing the gene expression patterns of the three samples, the coexistence of overdominance, dominance, underdominance and additivity was observed in the gene action modes of *Jiyan-1*. There were 2,237 transcripts in the intersection of the differentially expressed transcripts from *Jiyan-1* versus *T. rubripes* and *Jiyan-1* versus *T. flavidus*, among which 213 transcripts were also in the *T. rubripes* versus *T. flavidus*. The potential functions of the remaining 2,024 transcripts were mainly associated with metabolic process, nucleotide binding and catalytic activity. The enrichment results indicated metabolism was the most activated biological function in the heterosis. In addition, 35 KEGG pathways were retrieved as affiliated with more than three differentially expressed transcripts and 8,579 potentially novel transcript isoforms were identified for *Jiyan-1*. The present study revealed the coexistence of multiple gene actions in the hybrid puffer, indicated the importance of metabolism, ion binding function and kinase activity, as well as provided a list of candidate genes and pathways for heterosis. It could be helpful for the better understanding of the determination and regulation mechanisms of heterosis.

## Introduction

Pufferfishes of the genus *Takifugu* are mainly distributed in the inshore waters of China, Japan and Korea. They have been the most popular and expensive fishes in Japan for centuries because of their high protein content and special flavor [1], even though many of them contain fatal poison in the blood, liver and gonad. The pufferfishes have been artificial cultivated in Japan and China for decades, and there already is a big industry of pufferfish aquaculture in China. Tiger puffer (torafugu, *Takifugu rubripes*), and tawny puffer (*Takifugu flavidus*) are two representative species with different advantages. Tiger puffer possesses the largest body size in the *Takifugu* genus (total length is normally 40–50 cm, up to 80 cm; max body weight is more than 10 kg), and can attain a very fast growth rate under cultivation environment (reaching an average weight of 700 g after 18-month growth). It is naturally distributed in the Sea of Japan, the East China Sea and the Yellow Sea [2]. The edible history of tiger puffer was very long in Japan, which is why it was also called “Japanese puffer”. On the contrary, the habitat range of tawny puffer is much narrower, which is only distributed in the East China Sea, the Yellow Sea and the Bohai Bay [3]. Tawny puffer maintains a smaller body size (max total length is 40 cm) and slower growth rate (reaching an average weight of 150 g after 18-month growth) than tiger puffer. The relatively low economic yield of tawny puffer, coupled with the better flavor taste, make the price of tawny puffer to the highest among puffer species in China [3].

*Jiyan-1* puffer, the F_1_ hybrid of tiger puffer and tawny puffer, was artificially cultivated in China at 2006, to combine the desirable traits of the tiger puffer and the tawny puffer. The *Jiyan-1* puffer exhibits obvious hybrid vigor in the fields of growth performance, flavor and stress tolerance [4]. After 18-month growth, the body size of *Jiyan-1* is similar with tiger puffer and more than twice of tawny puffer and its weight is over 400 g averagely. The max total length of *Jiyan-1* can be over 80 cm, and max body weight can be more than 11 kg after 4-year growth. Moreover, *Jiyan-1* displays higher stress tolerance to environmental toxins, high sulfide concentration, low ambient temperature and water of variational pH value. The over-winter survival rate of *Jiyan-1* is over 60% and 30% higher than that of the parental puffer in outdoor cultivation and indoor cultivation, respectively. Besides, the taste flavor of *Jiyan-1* is considered to be equivalent with the tawny puffer, distinctly better than the tiger puffer.

The superiority of the hybrids was defined as heterosis by Shull [5] in 1908, and the phenomenon of heterosis has become a major strategy for increasing productivity and been exploited extensively in production of corps and livestocks such as maize, sorghum, rice, cattle and mule [6]–[9]. For pufferfish, natural hybrids occasionally appear, and the artificial cross breeding started decades ago [10]. But the detailed mechanism beneath heterosis remains unclear. The “dominance” and “overdominance” are two popular but competing genetic explanations for heterosis. The dominance hypothesis attributes the superiority of hybrids to the suppression of deleterious recessive alleles from one parent by dominant alleles from the other [11]. While the overdominance hypothesis refers to the allelic combination in the hybrid which make the heterozygous class performs better than either homozygous class [5]. Several system-wide studies have been applied with some corps for the better understanding of the heterosis genetic basis [12]–[14].

The sequencing technologies have been developed very fast in recent years, and the second generation sequence (Next Generation Sequence, NGS) technologies are already widely used for many applications [15]–[17]. The NGS-based transcriptome analyses have increasingly become routine tools for identifying quantitative differences among samples and detecting structural variants in transcripts. Due to the characteristics of *takifugu* puffer, such as the small but compact genome, similar gene quantity and exon length with other higher organisms [18], [19] and the heterosis superiority of the *Jiyan-1*, *Jiyan-1* is an excellent model for heterosis study on the genomic level.

In the present study, the whole transcriptomes of three individuals of *T. rubripes*, *T. flavidus* and *Jiyan-1* were sequenced using SOLiD 4 platform [20] with the aims to collect high coverage transcriptome data, to sort out and functionally analyze the differentially expressed transcripts among the three puffers and to identify the gene expression modes and novel transcripts of the hybrid. The information could help to ascertain candidate genes and pathways beneath heterosis and understand the determination and regulation mechanisms of heterosis.

## Results

### Reads Generation and Alignment

Three transcriptome fragment libraries were built with ribo-minus RNA from the tiger puffer, tawny puffer and *Jiyan-1* puffer, and sequenced using SOLiD 4 Genetic Analysis System. A total of 82,479,484, 70,915,736, and 90,401,656 single end reads with length of 50 bp were obtained, respectively. The combined sequence length was over 12,189 Mbp, representing 31.2-fold coverage of the tiger puffer genome size.

**Figure 1:**
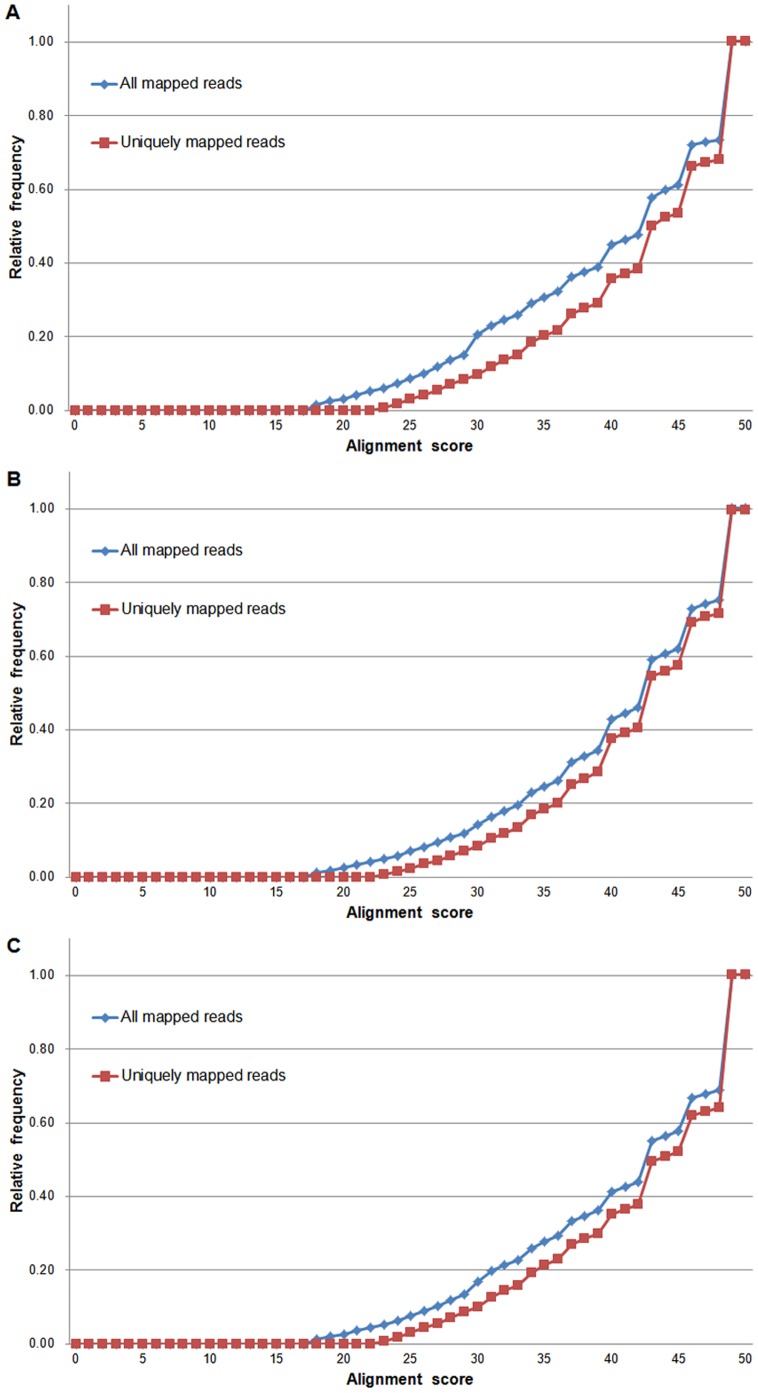
Cumulative distributions of reads alignment scores . **A.** Reads from tiger puffer. **B.** Reads from tawny puffer. **C.** Reads from *Jiyan-1* puffer.

After filtered with the sequences of adapter, Poly-N and known non-coding RNAs, 14,001 reads were removed. For tiger puffer, tawny puffer and *Jiyan-1*, the successfully mapping rates were 49.8% (41,074,430 mapped reads), 62.5% (44,304,273 mapped reads) and 56.4% (51,003,170 mapped reads), respectively; and the uniquely mapping rates were 75.9%, 79.2% and 76.9%, respectively (Table 1). 100% of uniquely mapped reads and 99.4% of all mapped reads were aligned with an alignment score above 20; and 98.7% of uniquely mapped reads had an alignment score above 30 (Figure 1).

**Table 1:** Transcriptome mapping statics.

Sample	Reads generated	Reads mapped	Mapping rate	Reads uniquely mapped	Uniquely mapping rate
Tiger puffer	82,479,484	41,074,430	49.8%	31,028,999	75.5%
Tawny puffer	70,915,736	44,304,273	62.5%	35,089,656	79.2%
*Jiyan-1*	90,401,656	51,003,170	56.4%	39,219,548	76.9%
Total	243,796,876	136,381,873	55.9%	105,338,203	77.2%

Mapping rate: mapped reads/all generated reads;

Uniquely mapped rate: uniquely mapped reads/all mapped reads * 100%.

### Transcript Abundance

There were 44,305 transcripts (corresponding to 18,164 genes) in the union set of three samples, which was 91.0% and 93.7% of all transcripts and genes in the reference (Table 2). Among them, 30,291 transcripts, corresponding to 15,229 genes, were in the intersection of the three samples. The transcript numbers were 36,760, 38,238 and 38,178, corresponding to 16,399, 16,912 and 17,032 genes, for tiger puffer, tawny puffer and *Jiyan-1* puffer respectively. The numbers of unique transcripts (only in one sample) were 1,676, 2,096 and 1,953, corresponding to 325, 470 and 517 genes (Table S3). The numbers of transcripts with FPKM>100 (FPKM: Fragments Per Kilobase of exon per Million fragments mapped) in each sample were 836, 1,101 and 1,427. The 100 most abundant transcripts were aligned with nr database using Blastn (*E*-value<1E−5), and most of them were identified as small nucleolar RNA, spliceosomal RNA and miRNA (86, 85 and 93 in in tiger puffer, tawny puffer and *Jiyan-1*, respectively). There were 19,444, 17,136 and 14,910 low abundant transcripts with FPKM<1, accounting for 52.9%, 44.8% and 39.1% of total transcripts in tiger puffer, tawny puffer and *Jiyan-1* puffer, respectively. (Table S1).

**Table 2:** Gene and transcript abundance summary.

Sample	Number of transcripts				Number of genes			
	All	Unique	FPKM<1	FPKM>100	All	Unique	Cov<0.2	Cov>0.8
Tiger puffer	36,760	1,676	19,444	836	16,399	325	7,039	3,154
Tawny puffer	38,238	2,096	17,136	1,101	16,912	470	5,179	5,384
*Jiyan-1*	38,178	1,953	14,910	1,427	17,032	517	5,080	5,634
Total	44,305				18,164			

All: all transcripts/genes identified (FPKM>0). Unique: only in the corresponding sample. FPKM: Fragments Per Kilobase of exon per Million fragments mapped. Cov: coverage, when coverage is equal to1, the gene locus is fully covered by reads.

### Differentially Expressed Transcripts

**Figure 2:**
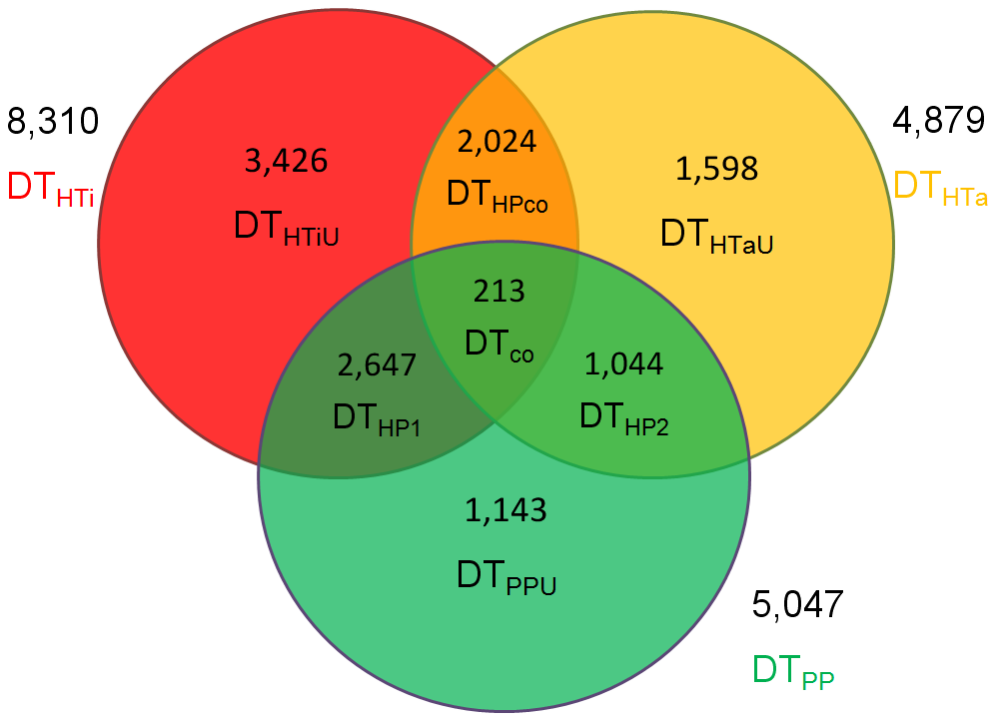
Grouping of differentially expressed transcripts. DT_HTi_, DT_HTa_ and DT_PP_ referred to DT from *Jiyan-1* versus tiger puffer, *Jiyan-1* versus tawny puffer, tiger puffer versus tawny puffer. DT_HTiU_, DT_HTaU_ and DT_PPU_ represented DT unique in corresponding groups. DT_HP1_, DT_HP2_ and DT_HPco_ were DT found in both two groups (DT_HTi_+DT_PP_, DT_HTi_+DT_PP_ and DT_HTi_+DT_HTa_, respectively). DT_co_ were DT found in all three comparisons.

There were 14,148 differentially expressed transcripts (DTs) in three comparison groups (*Jiyan-1* versus tiger puffer, *Jiyan-1* versus tawny puffer and tiger puffer versus tawny puffer), which were corresponding to 7,404 genes. The DTs were classified as DT_PP_ (DTs from tiger puffer versus tawny puffer), DT_HTi_ (DTs from *Jiyan-1* versus tiger puffer) and DT_HTa_ (DTs from *Jiyan-1* versus tawny puffer). Seven sub-groups, DT_HTiU_, DT_HTaU_, DT_PPU_, DT_HPco_, DT_HP1_, DT_HP2_ and DT_co_ were distinguished according to the relationship (Figure 2, Table S4). The expression patterns of all DTs in *Jiyan-1* were examined and grouped (Table 3). For the 10,952 DTs in *Jiyan-1*, 7,529 transcripts (68.7%) were designated as “above high parent” because the expression levels in the hybrid were significantly higher than that in both parental samples. 742 transcripts (6.8%) were classified as “below low parent” because their expression levels in the hybrid were significantly lower than that in both parents. 507 transcripts (4.6%) were “mid-parent” with the expression levels approximate to the mean values of two parental samples. 1,132 transcripts (10.3%) and 1,042 transcripts (9.5%) were designated as “low parent” and “high parent”, respectively, because the expression levels in *Jiyan-1* were approximate to that in the parent with the lower or higher expression level. For DT_co_, DT_HPco_, DT_HTiU_ and DT_HTaU_, the major part of DTs were classified as “above high parent”, while the major part of DT_HP2_ were classified as “low parent”. For DT_HP1_, the “above high parent” transcripts (1,173) were almost as many as the union set of transcripts from the “low parent” and the “high parent” (1,060).

**Table 3:** *Jiyan-1* gene action summary.

	Below_low	Low_parent	Mid_parent	High_parent	Above_high
DT_HTiU_	187	65	1	79	3,094
DT_HP1_	7	210	407	850	1,173
DT_HPco_	237	0	0	0	1,787
DT_co_	16	35	20	0	142
DT_HTaU_	289	176	6	13	1,114
DT_HP2_	6	646	73	100	219
Total	742	1,132	507	1,042	7,529

The numbers of transcripts were shown in the table. Below_low: expression level in *Jiyan-1* was lower than that in both parental samples. Low_parent: expression level in *Jiyan-1* was approximate to that in the parent with the lower expression level. Mid_parent: the expression level in *Jiyan-1* was approximate to the mean value of two parental samples. High_parent: expression level in *Jiyan-1* was approximate to that in the parent with the higher expression level. Above_high: expression level in *Jiyan-1* was higher than that in both parental samples.

### GO and KEGG Annotation of DT_HPco_

**Figure 3:**
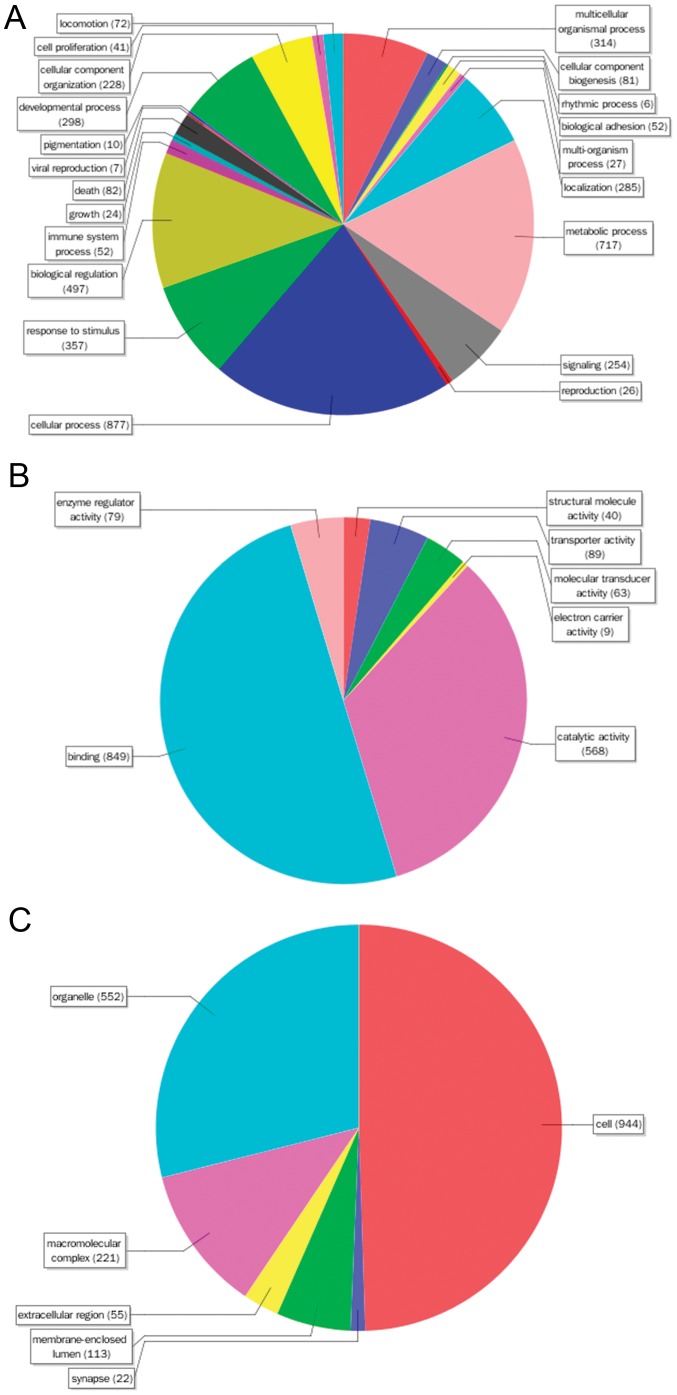
Sequence distribution by GO at level 2 for DT_HPco_. **A.** biological process. **B.** molecular function. **C.** cellular component. Numbers shown in the chart represented the amount of sequences with corresponding GO terms.

There were 2,237 differentially expressed transcripts in both of the comparison groups of *Jiyan-1* versus *T. rubripes* and *Jiyan-1* versus *T. flavidus*. Among them, 2,024 were not in the *T. rubripes* versus *T. flavidus* (DT_HPco_), and 213 were in all three comparison groups (DT_co_). DT_PP_, the differentially expressed transcripts in the comparison of tiger puffer versus tawny puffer (including DT_PPU_, DT_HP1_, DT_HP2_ and DT_co_), donated the differences between tiger puffer and tawny puffer, likewise DT_HPco_ were more likely related to the superior traits of hybrid offspring. The GO (Gene Ontology) functional analysis of DT_HPco_ was applied and 1,538 transcripts were assigned with GO terms. The GO terms were summarized into three main categories: biological process, molecular function and cellular component (Figure 3 and Figure S1). In biological process category, the top-3 GO terms were cellular process (20.4%), metabolic process (16.6%) and biological regulation (11.5%). In the category of molecular function, binding (50.0%), catalytic activity (33.5%) and transporter activity (5.2%) accounted for the major portion. And the cellular component category mainly consisted of cell (49.5%), organelle (28.9%) and macromolecular complex (11.6%).

**Figure 4:**
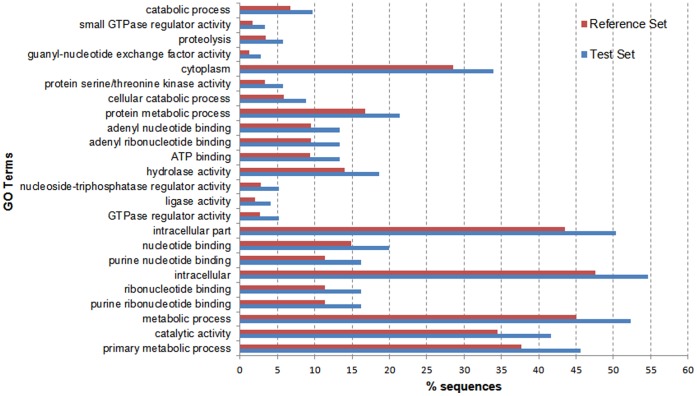
The enrichment analysis result for DT_HPco_. The percentages of sequences for test and reference set were shown in the bar chart in ascending order of significance level.

The distributions of enriched GO term were shown at multiple GO levels in Figure 4. The major enriched GO terms in Biological Process category were (FDR<7.44E−6), metabolic process (FDR<5.11E−5), protein metabolic process (FDR<2.10E−3), cellular catabolic process (FDR<2.33E−3), catabolic process (FDR<7.69E−3) and proteolysis (FDR<5.36E−3). In the Molecular Function category, the enriched GO terms were catalytic activity (FDR<5.11E−5), purine nucleotide binding (FDR<7.12E−5), purine ribonucleotide binding (FDR<7.12E−5), nucleoside-triphosphatase regulator activity (FDR<1.88E−4), GTPase regulator activity (FDR<2.81E−4), ligase activity (FDR<2.81E−4), hydrolase activity (FDR<5.86E−4) and protein serine/threonine kinase activity (FDR<2.37E−3). In addition, intracellular (FDR<7.12E−5), intracellular part (FDR<1.99E−4) and cytoplasm (FDR<2.54E−3) were enriched in the category of Cellular Component (Table S5).

The enriched transcripts with level-4 GO terms were classified according to the fold change value of abundance (Table 4). 61.5% of enriched transcripts (735+148) in *Jiyan-1* had absolute values of ln values above 2, which donated a more dramatic variation of expression level. Among the 1,184 enriched differentially expressed transcripts, 783 transcripts (66.1%) were related to the metabolism function (primary metabolic process, metabolic process, protein metabolic process, cellular catabolic process, proteolysis and catabolic process categories in Table S5).

**Table 4:** Distribution of transcripts in enriched GO terms at level 4.

GO terms	Num of Up1	Num of Up2	Num of Down1	Num of Down2	FDR
Intracellular	215	296	14	63	7.12E−5
Nucl B	76	111	9	21	1.88E−4
GTPase A	17	26	2	7	2.81E−4
Ligase A	23	15	5	3	2.81E−4
Hydro A	65	110	4	25	5.86E−4
Pro MP	72	136	5	22	2.10E−3
Cell CP	43	41	3	7	2.33E−3
Total	511	735	42	148	

Nucl B: Nucleotide binding, GTPase A: GTPase regulator activity, Ligase A: Ligase activity, Hydro A: Hydrolase activity, Pro MP: Protein metabolic process, Cell CP: Cellular catabolic process, FDR: False Discovery Rate. Up1: Up-regulated group1, 0>ln(x/hybrid)> −2; Up2: Up-regulated group2, −2>ln(x/hybrid); Down1: Down-regulated group1, 2>ln(x/hybrid)>0; Down2: Down-regulated group2, ln(x/hybrid)>2.

There were 80 KEGG pathways successfully retrieved, among which 35 pathways contained more than three transcripts from DT_HPco_ (Figure S2). After sorted by the number of mapped transcripts, the top-5 KEGG pathways were purine metabolism pathway (34), phosphatidylinositol signaling system pathway (17), inositol phosphate metabolism pathway (14), pyruvate metabolism pathway (11) and alanine aspartate/glutamate metabolism pathway (10).

### Potentially Novel Transcript Isoforms in Jiyan-1

There were 11,700, 14,568 and 14,680 transfrags with at least one splice junction shared with reference transcripts in the transcriptome of the tiger puffer, the tawny puffer and *Jiyan-1*, respectively. From them, 8,579 potentially novel transcript isoforms were identified to *Jiyan-1*, corresponding to 3,476 genes (Table S7). The longest isoform was 19,534 bp, and the shortest was 211 bp. The average length of all potentially novel isoforms was 2,630 bp. There were 316 genes (9.1%) with more than three potentially novel isoforms and 66.8% of them (211 genes) were annotated to have functions related to the binding function for ion, nucleic acid and protein. The genes with the most novel transcript isoforms were *IPO4*, *LPIN1* and ENSTRUG00000005582 (6 novel isoforms).

The sequence similarity analysis revealed that 8,176 transcript isoforms were prior matched by the sequences from pufferfish (*T.rubripes* and *T. nigroviridis*). The remaining 403 transcript isoforms preserving a high similarity with transcripts from other organisms, for instance, 301 isoforms (74.7%) were similar with transcripts of the Nile tilapia (*O. niloticus*) and 22 isoforms (5.5%) were similar with transcripts of the zebrafish (*D. rerio*). The 9,278 potential protein sequences were obtained with 3-frame translation and subsequently used for function prediction. There were 1,763, 476 and 547 protein signatures (domains/superfamilies) identified for the Pfam, SMART and SUPERFAMILY database, and the most abundant ones were mainly involved in the ion binding, protein binding and kinase activity (Table 5 and Table S8).

**Table 5:** Top 10 annotations of novel transcript isoforms.

	Annotations of Pfam			Annotations of SMART				
Rank	Entry ID	Number	Description	Entry ID	Number	Description		
1	PF00096	557	Zinc finger, zinc ion binding	SM00355	958		Zinc finger, zinc ion binding	
2	PF00400	534	WD40 repeat, protein binding	SM00181	875		Epidermal growth factor-like	
3	PF01391	423	Collagen triple helix repeat	SM00320	728		WD40 repeat, protein binding	
4	PF00041	415	Fibronectin type III, protein binding	SM00248	555		Ankyrin repeat, protein binding	
5	PF07679	321	Immunoglobulin I-set	SM00179	526		EGF-like calcium-binding, calcium ion binding	
6	PF00069	297	Protein kinase activity, ATP binding, phosphorylation	SM00409	491		Immunoglobulin subtype	
7	PF00028	238	Cadherin, calcium ion binding, homophilic cell adhesion	SM00060	467		Fibronectin type III, protein binding	
8	PF07645	234	EGF-like, calcium ion binding	SM00220	357		Serine/Threonine kinase activity, ATP binding, protein phosphorylation	
9	PF00595	201	PDZ/DHR/GLGF	SM00408	350		Immunoglobulin subtype 2	
10	PF12796	200	Ankyrin repeat-containing domain	SM00219	310		Protein tyrosine kinase activity	

## Discussion

Heterosis, or hybrid vigor, refers to the phenomenon that the cross breeding offspring can combine the desirable traits of two parental species, even exhibits superiority which never appear on parents. The incentives beneath heterosis are undoubtedly complicated, and they have been the subjects of intense research and speculation for over a century. Even several genetic hypotheses of heterosis have been emerged, such as the “dominance” and “overdominance”, the basic mechanisms remain unclear [21]–[23].

To promote the understanding of heterosis mechanisms, the transcriptome of *Jiyan-1* hybrid puffer, as well as transcriptomes of the two parental species, were sequenced by the SOLiD 4 NGS platform for comparative analysis in the present study. 55.9% of all reads were successfully aligned. The uniquely mapping rates were similar among three puffers (from 75.5% to 79.2%), suggesting that their genomes were quite alike. The transcripts abundance analysis (Table 2, Table S1 and Table S2) revealed the most transcripts were of low abundance, which might be due to basal expression or rapid degradation, as well as demonstrated the transcriptome data in the present study was sensitive enough for the detection of traces of RNA. The abundant transcripts were mainly house-keeping transcripts such as small nucleolar RNA and miRNA, which were mainly involved in the pre-mRNA processing, RNA splicing and gene regulation [24]. It was corresponded with the fact that the puffers used in the study were under rapid growth period.

Among the 44,305 transcripts identified in the present study, 14,148 transcripts were differentially expressed among the three puffers (Figure 2 and Table S4). The differentially expressed transcripts between *Jiyan-1* and tiger puffer were about twice as many as that between *Jiyan-1* and tawny puffer, suggesting the transcription repertoire of *Jiyan-1* was more similar to that of tawny puffer. Meanwhile, the major of differentially expressed transcripts in *Jiyan-1* were up-regulated, indicating the transcription was highly activated in *Jiyan-1*. Moreover, 68.7% of all DTs were classified as “above high parent” that the expression levels in *Jiyan-1* were significantly higher than that in both parental samples. According to the “overdominance” hypothesis, the heterozygous gene in the hybrid performed better than either homozygous class, normally in the way of up-regulated gene expression level [25]. The data undoubtedly confirmed the existence of overdominant gene action mode. Besides, there were 6.8% DTs as “below low parent” which indicated the underdominant gene action mode. It was an interesting possibility that the co-existence of underdominant and overdominant gene action was related to the action of miRNA which could regulate gene expression by cleaving target mRNAs and transcriptional silencing [26]. And in this study, there were 16 miRNA in the top100 most abundant genes for *Jiyan-1* in the present study. On the other hand, 10.3% and 9.5% of the DTs were classified as “low parent” and “high parent” that the expression levels in *Jiyan-1* were approximate with that in the parent of either the higher expression levels or lower expression levels. These 19.8% transcripts exhibited the dominant gene action mode as the “dominance” hypothesis claimed, that deleterious recessive alleles from one parent were suppressed by dominant alleles from the other and the expression levels in the hybrid would be similar to the dominant one [6], [12], [14], [27]. Besides, 4.6% of all DTs (mid-parent) exhibited expression patterns that are not statistically distinguishable from additivity. The major of DTs exhibited overdominant action mode for all sub-groups except for the DT_HP1_ which the transcripts with dominant action mode was as many as the overdominant action mode. It indicated the overdominant action might be the main gene action mode in the *Jiyan-1*, as well as dominance, underdominance and additivity also coexisted in *Jiyan-1*. The finding was consistent with the hypothesis that multiple mechanisms contribute to the heterosis from previous results [25], [28], [29].

In order to determine the key components and regulatory network underlying the superior phenotypes, especially the novel ones in the hybrid, GO and KEGG analysis were applied to the 2,024 transcripts of DT_HPco_ which were in the intersection of DTs from *Jiyan-1* versus tiger puffer and DTs from *Jiyan-1* versus tawny puffer, but not in the comparison between tiger puffer and tawny puffer. The distribution of GO terms in the present study (Figure 3) was similar to previous studies of other organisms [30], [31]. Most transcripts were associated to cellular process and metabolic process (biological process), binding and catalytic activity (molecular function) and cell and organelle (cellular component) [30], [32], [33], which bespoke the increased activities of metabolism, growth and development in *Jiyan-1*. There was an interesting discovery that the transcripts related to metabolism were significantly enriched (783/1,184 = 66.1%), which indicated that metabolism was the most up-regulated function in *Jiyan-1*. The correlation of heterosis and enhanced metabolic activities was also reported in the hybrids of *Arabidopsis* [34]. As a result, it was suspected that the abnormal activities of metabolism were probably involved in some superior traits of *Jiyan-1* such as faster growth and large body size.

The enriched transcripts, especially the dramatically up-regulated/down-regulated ones in *Jiyan-1* (Table S5 and Table S6), were potential candidate contributors for the heterosis, such as GEF11 (Guanine nucleotide exchange factor 11, ENSTRUT00000001627), MEF2C (myocyte enhancer factor 2C, ENSTRUT00000002107) and MAPK6 (mitogen-activated protein kinase 6, ENSTRUT00000043718), which all expressed at remarkably higher levels in *Jiyan-1*. The MEF2C and MAPK6 were proved to response against inflammation and microbial infection, and functioned in host defense [35], [36]. And the alternation of GEF11 expression was observed more than once during acute and repeated stress [37]. Although there was no evidence about their potential contribution to heterosis, it was believed that these candidate genes took part in the determination and regulation of heterosis by controlling the activities of molecular switches in signaling pathways.

There were 35 KEGG pathways affiliated with more than three DT_HPco_ as complement elements which were predicted to be of more activities in *Jiyan-1*, and most of the pathways were involved in metabolism. The roles and contributions to heterosis of some pathways have been reported in other species. For example, the *Arabidopsis* allelic hybrids of the auxotrophic mutants of the thiamine pathway and the pyrimidine pathway exhibited strong complementary effect and gave rise to the superiority of growth comparing with homozygous auxotrophs [38]. But, the knowledge about the effects of most pathways on heterosis is still limited. It will be helpful to infer the potential roles of candidate pathways in the determination or regulation of heterosis based on the comprehensive consideration of the known functions of mapped components and the entire pathways.

The xenobiotics and drug metabolism pathways mediated by cytochrome P450 (CYP) may contribute to the higher survival of *Jiyan-1*. In the present study, four CYP transcripts (ENSTRUT00000001084, ENSTRUT00000035401, ENSTRUT00000040597 and ENSTRUT00000047091) were enriched in the GO enrichment analysis, and they were also assigned to the related KEGG pathways (Figure S2). Three transcripts were more abundant in *Jiyan-1*, and one of them (ENSTRUT00000047091, CYP family 24) was highest expressed in *Jiyan-1* which was about 80 times higher than that in other two, suggesting a distinctly higher enzyme activity of CYP in *Jiyan-1*. The CYP mediates the oxidation of xenobiotic substances such as drugs, insecticides and other toxic chemicals [39]. It has been confirmed that the xenobiotics and drug metabolism pathways mediated by CYP are extremely important for the resistance of insecticide and other toxic chemicals in plants, insects, animals and even humans [40]–[43]. Meanwhile, the overtranscription of CYP genes was also proved to be necessary and sufficient for the insecticide resistance [44]. Nowadays, the marine environment has been worsening because of the increasing discharge of pollutants and booming offshore human activity, which will surely have negative impacts on the pufferfish stock. The activation of the xenobiotics and drug metabolism pathways in *Jiyan-1* suggested the greater capability of the degradation and excretion of toxins, which consequently gave rise to the higher tolerance of hostile environment.

Metabolism of carbohydrate, amino acid and triglyceride are fundamental and crucial for the organisms to keep the normal physiological function. In the present study, several relative pathways with biased patterns of gene expression were revealed in *Jiyan-1*, such as four amino acid metabolism pathways (alanine aspartate and glutamate metabolism, glycine, serine and threonine metabolism, cysteine and methionine metabolism and valine leucine isoleucine degradation), five carbohydrate metabolism pathways (fructose and mannose metabolism, galactose metabolism, starch and sucrose metabolism, other glycan degradation and other types of O-glycan biosynthesis), two fatty acid metabolism pathways (fatty acid biosynthesis and fatty acid metabolism), two intermediate metabolism pathways (pyruvate metabolism and propanoate metabolism) and the TCA cycle pathway. Among all transcripts affiliated with these pathways, only two acetyl-CoA carboxylase transcripts (ENSTRUT00000025367 and ENSTRUT00000039731) were down-regulated in *Jiyan-1*, while all the other transcripts were dramatically up-regulated. It was proved that the hybrid maize and hybrid barley had higher respiratory control ratios and better integrity of the mitochondria due to the higher enzyme activities of the TCA cycle in the mitochondria [45], [46]. The relevance between heterosis and certain metabolism pathways, especially the TCA cycle pathway and its intermediates, was also established by the metabolite profiles in *Arabidopsis* [47]. The fatty acid and myelin compositions in the hybrid cattle and hybrid mice were found to be obviously different from their parents [48], [49]. In the present study, the expression alternation of genes involved in the metabolism related pathways suggested the different accumulation and consumption of carbohydrate, amino acid and fatty acid, which would consequently affect the composition of the body, and finally gave rise to the superiority of growth and development in *Jiyan-1*. The changed aminogram and fat content could also contribute to the great flavor of *Jiyan-1*. However, ESTs microarray analysis of rice hybrid [50] revealed that most genes functioning in carbohydrate metabolism, lipid metabolism, energy metabolism and protein degradation were down-regulated, and the genes functioning in amino acid metabolism were either up- or down-regulated. In the present study, there was no significant difference for the expression of most genes in the hybrid, while the majority of metabolism related genes in DT_HPco_ were up-regulated. The discord could be derived from the limited throughput of microarray and the species diversity between rice and pufferfish, which further indicated that the mechanisms underlying heterosis might be diverse among species, especially between plants and animals, and future studies should be deployed on various organisms.

The possible role of novel transcript isoforms in the heterosis was also studied. 11,700, 14,568 and 14,680 transfrags were identified from the tiger puffer, the tawny puffer and *Jiyan-1*, respectively. The quantity variance of identified transfrags between the tiger puffer and the tawny puffer was likely caused by the specific difference. The transcript size distribution of novel transcript isoforms showed no significant difference with the known transcripts in puffer. High sequence similarity with transcripts from other organisms was also observed. In the present study, there were 8,579 potentially novel transcript isoforms in *Jiyan-1*, which was about 22.5% of the known transcripts, while for more well-studied organisms, novel transcripts were also being discovered [51], [52]. The vast novel transcript isoforms suggested the variation of RNA splicing was more complex than expected in pufferfish, even though the number was maybe overestimated due to the algorithmic limitation. The functional annotation revealed that most protein products of the novel transcripts were involved in the functions related to ion binding and kinase activity, such as zinc ion binding, calcium ion binding and Serine/threonine-protein kinase (Table 5, Table S7 and Table S8). Multiple isoforms of zinc finger proteins and Ser/Thr kinase have been widely observed and found to be essential for the normal function in body [53], [54]. Therefore, the binding function and kinase activity might contribute to the regulation of heterosis of *Jiyan-1*. In addition, three genes (*IPO4*, *LPIN1* and ENSTRUG00000005582) were identified with six potentially novel transcript isoforms. The protein product of *LPIN1* was regarded as a key regulator of lipid metabolism and the product of the ENSTRUG00000005582 was believed to be involved in the glycosaminoglycan metabolism [55]. Moreover, there were already five and six known isoforms for the *LPIN1* and ENSTRUG00000005582 gene in pufferfish, respectively. The large quantities of transcript isoforms corroborated the analysis result of differentially expressed transcripts and emphasized the potentially important role of metabolism in the heterosis once again.

In conclusion, the comparative analysis of the deep sequencing transcriptome data indicated the overdominance was the main gene action mode, and confirmed the coexistence of multiple gene action modes including overdominance, dominance, underdominance and additivity in the *Jiyan-1* puffer. The present study highlighted the possibility that multiple molecular mechanisms contribute to the heterosis. The metabolism was revealed to be the most activated biological function in the *Jiyan-1* by the GO annotation of differentially expressed genes and novel transcript analysis. In addition, there were 1,184 transcripts and 35 pathways were identified as potential contributors for the determination and regulation of heterosis in *Jiyan-1*, which could be excellent object for polymorphism analysis, alternative splice discovery and functional studies. The possible role of ion binding protein and kinase enzyme in the heterosis was also pointed out by the novel transcript study. The information could be helpful for both the fundamental study and applied research of pufferfish.

## Materials and Methods

### Ethics Statement

The pufferfish used in the present study are aquaculture cultured animals, and all the experiments were conducted in accordance with the recommendations in the Guide for the Care and Use of Laboratory Animals of the National Institutes of Health. The study protocol was approved by the Experimental Animal Ethics Committee, Institute of Oceanology, Chinese Academy of Sciences, China. Spinal tap was carried out to minimize suffering before the dissection and tissue collection.

### Biological Material and RNA Extraction

All pufferfish used in the study have been cultured in Hebei Institute of Fisheries for research purpose. The tawny puffer and tiger puffer are both from inbred lines produced through several generations of sister-brother mating. The hybrid puffers are the F1 offspring of these two lines, which are siblings within lines and half-blooded between lines. All puffers were cultivated under the same breeding conditions until five month old when the superiorities of growth rate and livability of hybrid puffer began to show. One individual was randomly selected from the populations of tiger puffer, tawny puffer and *Jiyan-1* hybrid puffer, respectively. After dissection, tissues of blood, muscle, kidney, liver, gill and spleen were immediately sampled and kept in RNALater (Ambion) [56] at 4°C, and then stored at −80°C. Total RNA was separately extracted with liquid nitrogen and TRIzol (Invitrogen), and kept at −80°C until use.

### Library Preparation

Total RNA was quantified by Nanodrop 2000 (Thermo Scientific) and checked for the integrity with Angilent 2100 Bioanalyzer (Agilent Technologies). The RNA from six tissues of one individual was pooled in equal proportions as one sample. Twenty micrograms of mixed RNA from each sample was employed for the rRNA depletion. The ribosomal RNA was removed with RiboMinus Eukaryote Kit for RNA-Seq (Invitrogen) following the manufacturer’s instructions. There was 2–4 µg ribo-minus RNA left for each sample.

The single-end fragment library was constructed following the SOLiD Total RNA-Seq Kit protocol (Life Technologies, PN4452437). Ribo-minus RNA was fragmented by RNase III and purified using the RiboMinus Concentration Module (Invitrogen). RNA fragment was linked with the adaptor using the hybridization master mix (SOLiD Total RNA-Seq Kit), and reverse transcription was performed subsequently. The purified cDNA was size selected after DNA electrophoresis with the Novex TBE-Urea Gel (Invitrogen) at 180 V for 20 min. The gel block containing 150–250 nt cDNA was precisely excised and used as amplification template. The PCR reactions were performed at 95°C for 5 min and then cycled at 95°C for 30 sec, 62°C for 30 sec and 72°C for 30 sec for 15 cycles in a thermal cycler. All of the components used in the amplification were from the SOLiD Total RNA-Seq Kit. The yield and size distribution of PCR products were checked by Agilent 2100 Bioanalyzer.

Emulsion PCR and bead enrichment were performed using the SOLiD EZ Bead™ system (Life Technologies). Workflow analysis (WFA) was performed first to verify the quality and density of the template beads. About 120 million enriched beads for each sample were then deposited on the sequencing slide. Finally, the libraries were sequenced on the SOLiD 4 platform and color-space reads were outputted. The raw sequencing reads have been submitted to NCBI Short Read Archive under the accession number of SRA058916.

### Bioinformatics Analysis

The reads alignment was performed using BioScope™ software (Life Technologies) with default parameters. The genome of *Takifugu rubripes* (v4.64) was downloaded from Ensembl [57] as the reference. The classic mapping strategy “seed-and-extend” approach was adopted, with “25.2.0∶20” as mapping scheme (for the 50 base reads, the seed might be 25 base long with up to two mismatches allowed, and the start site of seed could be 0 or 20). Reads alignment score was calculated as: score = len-nm*1(1+mp)−jp, where len = number of alignment hits (colors for SOLiD reads), nm = mismatch number, mp = mapping mismatch penalty, jp = penalty for alignment to a junction. After alignment, only the unique alignments or the alignments sufficiently better than any suboptimal hits could be outputted.

For abundance analysis, a home-made Perl script was used to extract the gene loci coordinates from the gene set file of *T. rubripes* downloaded from Ensembl. The gene loci coverage was estimated using the CoverageBed tool from BEDtools [58]. Cufflinks [59] was used to calculate transcript abundances and identify the DT based on the mapping results. The variation of transcripts abundance was described by fold change value: ln(x/*Jiyan-1*), while two ln (x/*Jiyan-1*) values were calculated separately for each transcript (x = tiger puffer or tawny puffer).

BioMart [60] was used for GO identifier retrieving and GO terms assignment of all tiger puffer (*T.rubripes*) sequences. For other sequences, local blastp search was first performed to align sequences to the non-redundant database of NCBI with *E*-value<1E−5. Then alignment result was parsed by Blast2GO [61] for assigning GO terms with parameters of *E*-value<1E−5, annotation cutoff >55 and GO weight >5. The GO enrichment analysis was implemented by the one-tailed Fisher’s exact test with filter value was set as 0.01. DT_HPco_ were selected as test set while all identified transcripts were taken as the reference set. The GO terms which were significantly over-enriched in test set were reported. DT_HPco_ were mapped to the KEGG database [62], using built-in function of Blast2GO for the pathway retrieving. All mapping and retrieving steps were performed by default setting.

The novel transcript prediction was performed by the Reference Annotation Based Transcript (RABT) assembly of Cufflinks. The mapping results (bam files) were assembled with the aid of reference annotation. Potentially novel transcript isoform was defined as at least one splice junction was shared with a reference transcript. Three predicted transcript annotation files were compared with the *T.rubripes* annotation and each other using cuffcompare. Potentially novel isoforms with low evidence number were discarded and the remaining ones were summarized by home-made Perl script. Sequences of predicted transcripts were extracted by the gffread tool. Sequence alignment was performed using blast+ [63]. The protein sequences were obtained with 3-frame translation, and only the sequences with the length from 50aa to 3,000aa and no premature stop codon were used for the annotation by InterProScan [64].

## Supporting Information

Figure S1 **Distribution of third level GO terms for DT_HPco_.**
(PDF)

Figure S2 **Thirty-five KEGG pathway maps with more than 3 DT_HPco_.**
(PDF)

Table S1 **FPKM values of transcripts.**
(XLSX)

Table S2 **Distribution of gene loci coverage.**
(XLSX)

Table S3 **Summary of unique genes and transcripts.**
(XLSX)

Table S4 **Differentially expressed transcripts in sub groups.**
(XLSX)

Table S5 **Enriched GO terms and sequences in DT_HPco_.**
(XLSX)

Table S6 **Annotation for all enriched sequence.**
(XLSX)

Table S7 **Potentially novel transcripts and GO annotation.**
(XLSX)

Table S8 **Novel transcripts functional prediction.**
(XLSX)
