# Supplementary figures and images for: Transcriptome Analysis of Artificial Hybrid Pufferfish Jiyan-1 and Its Parental Species: Implications for Pufferfish Heterosis
Source: PLoS One. 2013 Mar 8;8(3):e58453. doi: 10.1371/journal.pone.0058453 (PMC3592836; doi:10.1371/journal.pone.0058453)

# biological\_process Level 3

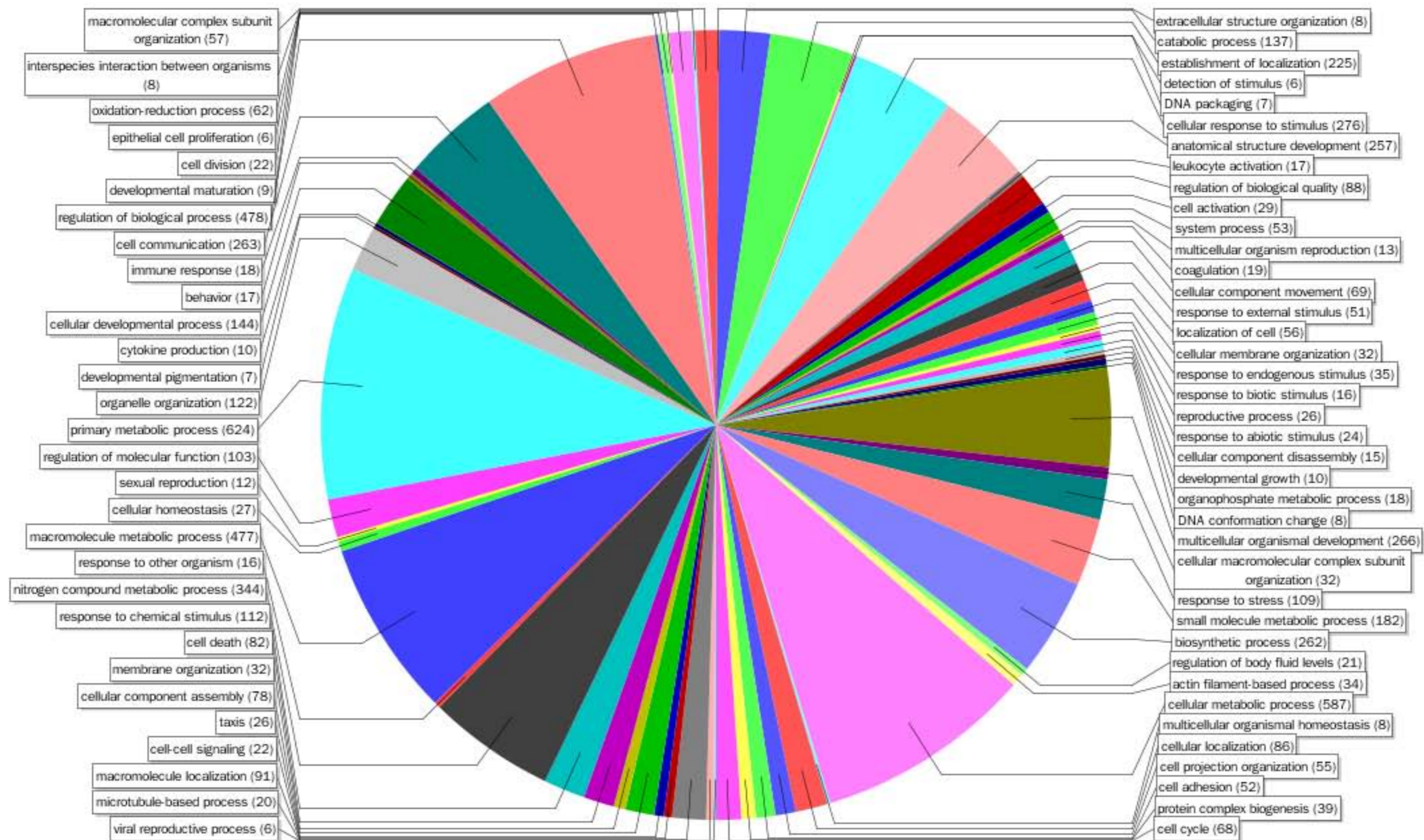

# molecular\_function Level 3

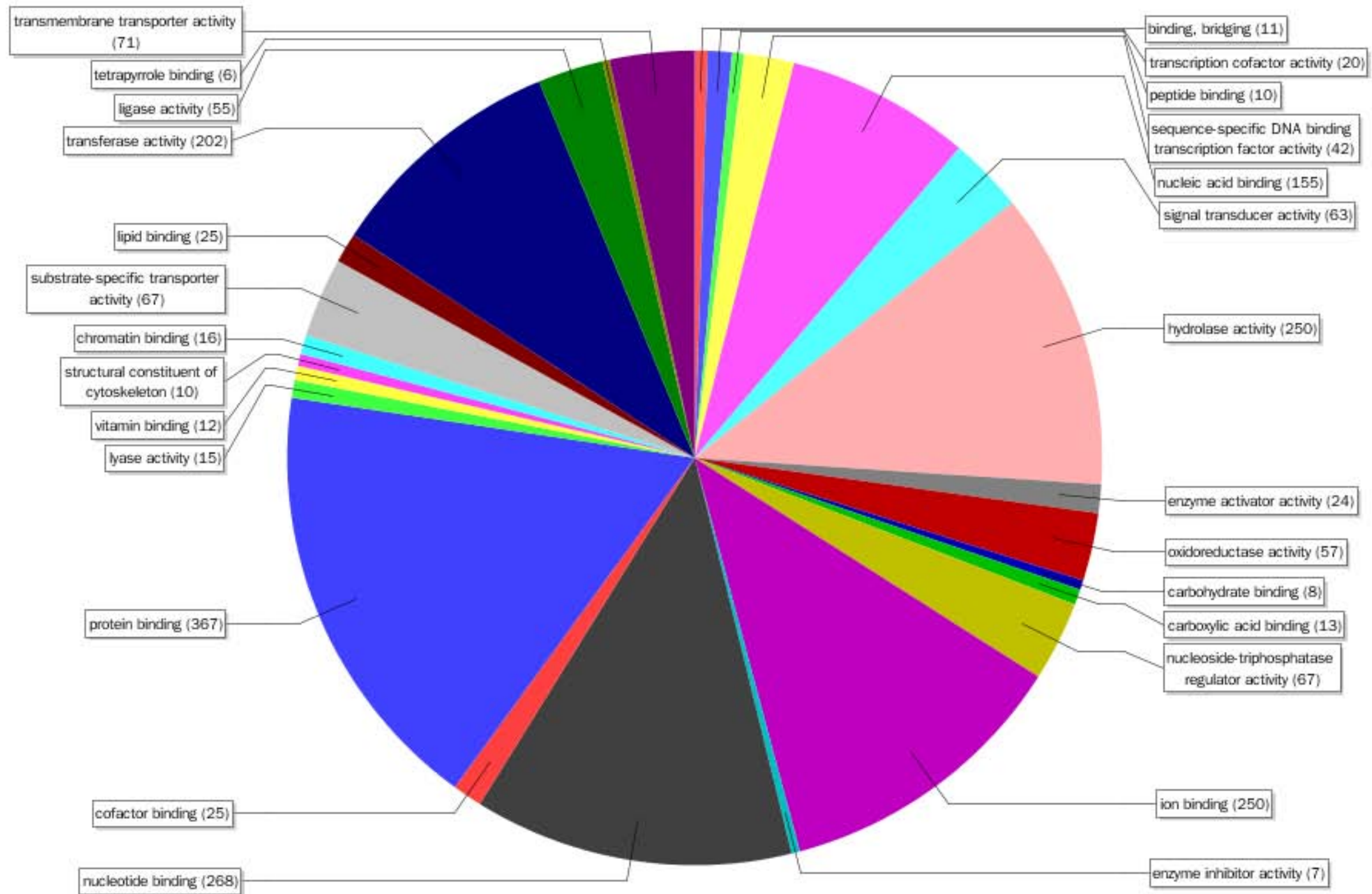

# cellular\_component Level 3

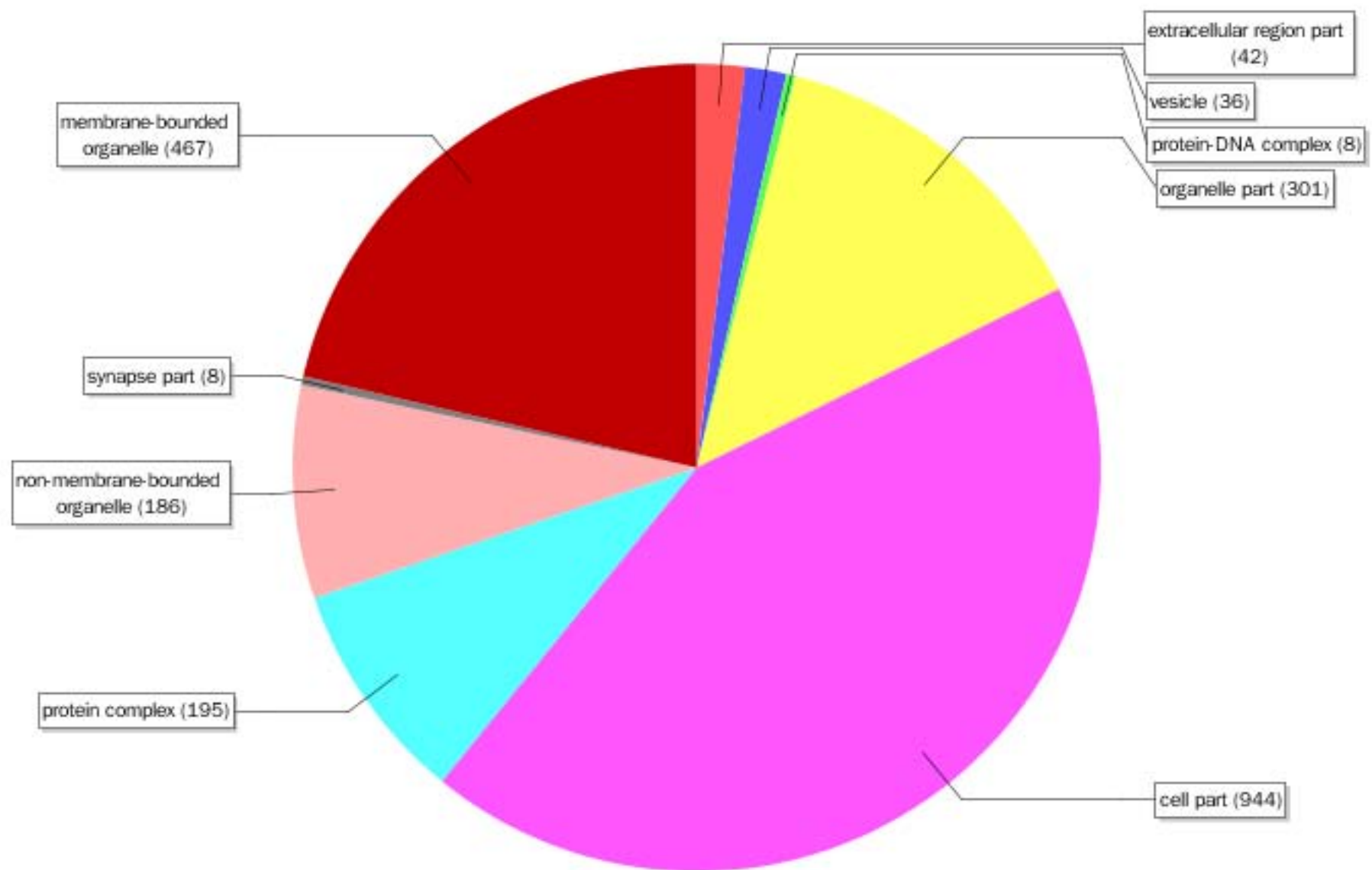

Supplement: Figure S1 — Distribution of third level GO terms for DTHPco. (PDF) [file pone.0058453.s001.pdf]
