# Supplementary material for: Transcriptome Analysis of Artificial Hybrid Pufferfish Jiyan-1 and Its Parental Species: Implications for Pufferfish Heterosis
Source: PLoS One. 2013 Mar 8;8(3):e58453. doi: 10.1371/journal.pone.0058453 (PMC3592836; doi:10.1371/journal.pone.0058453)
Supplement: Figure S2 — Thirty-five KEGG pathway maps with more than 3 DTHPco. (PDF) [file pone.0058453.s002.pdf]

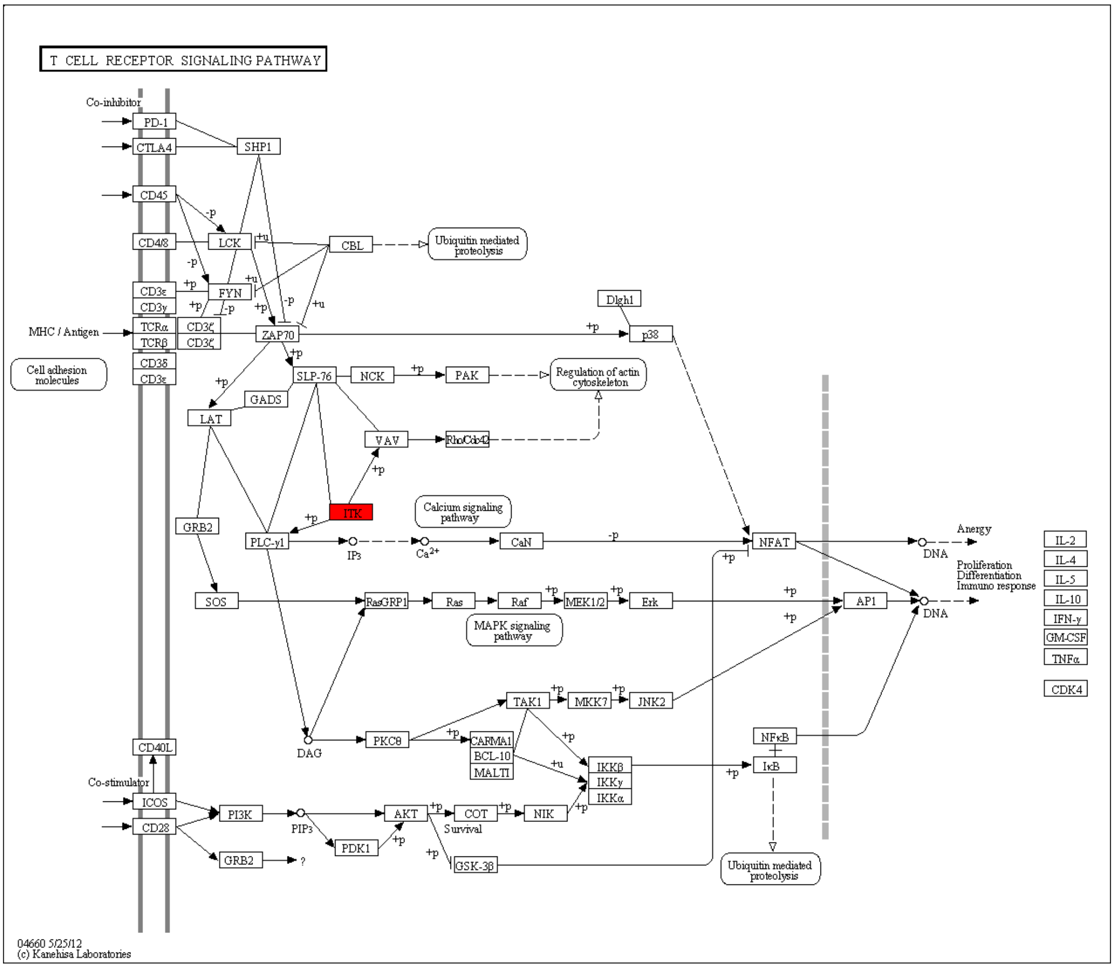
T CELL RECEPTOR SIGNALING PATHWAY
Co-inhibitor
PD-1
CTLA4
SHP1
CD45
CD4/8
LCK
CBL
Ubiquitin mediated proteolysis
CD3ε
CD3γ
FYN
MHC / Antigen
TCRα
TCRβ
CD3ζ
CD3ζ
ZAP70
Dlgh1
p38
Cell adhesion molecules
CD3δ
CD3ε
SLP-76
NCK
PAK
Regulation of actin cytoskeleton
GADS
LAT
VAV
Rho/Cdc42
ITK
Calcium signaling pathway
GRB2
PLC-γ1
IP3
Ca2+
CaN
NFAT
DNA
Anergy
Proliferation
Differentiation
Immuno response
IL-2
IL-4
IL-5
IL-10
IFN-γ
GM-CSF
TNFα
CDK4
SOS
RasGRP1
Ras
Raf
MEK1/2
Erk
AP1
DNA
MAPK signaling pathway
TAK1
MKK7
JNK2
DAG
PKCθ
CARMA1
BCL-10
MALT1
IKKβ
IKKγ
IKKα
NFκB
IκB
CD40L
Co-stimulator
ICOS
CD28
PI3K
PIP3
AKT
COT
NIK
Survival
PDK1
GRB2
?
GSK-3β
Ubiquitin mediated proteolysis
04660 5/25/12
(c) Kanehisa Laboratories

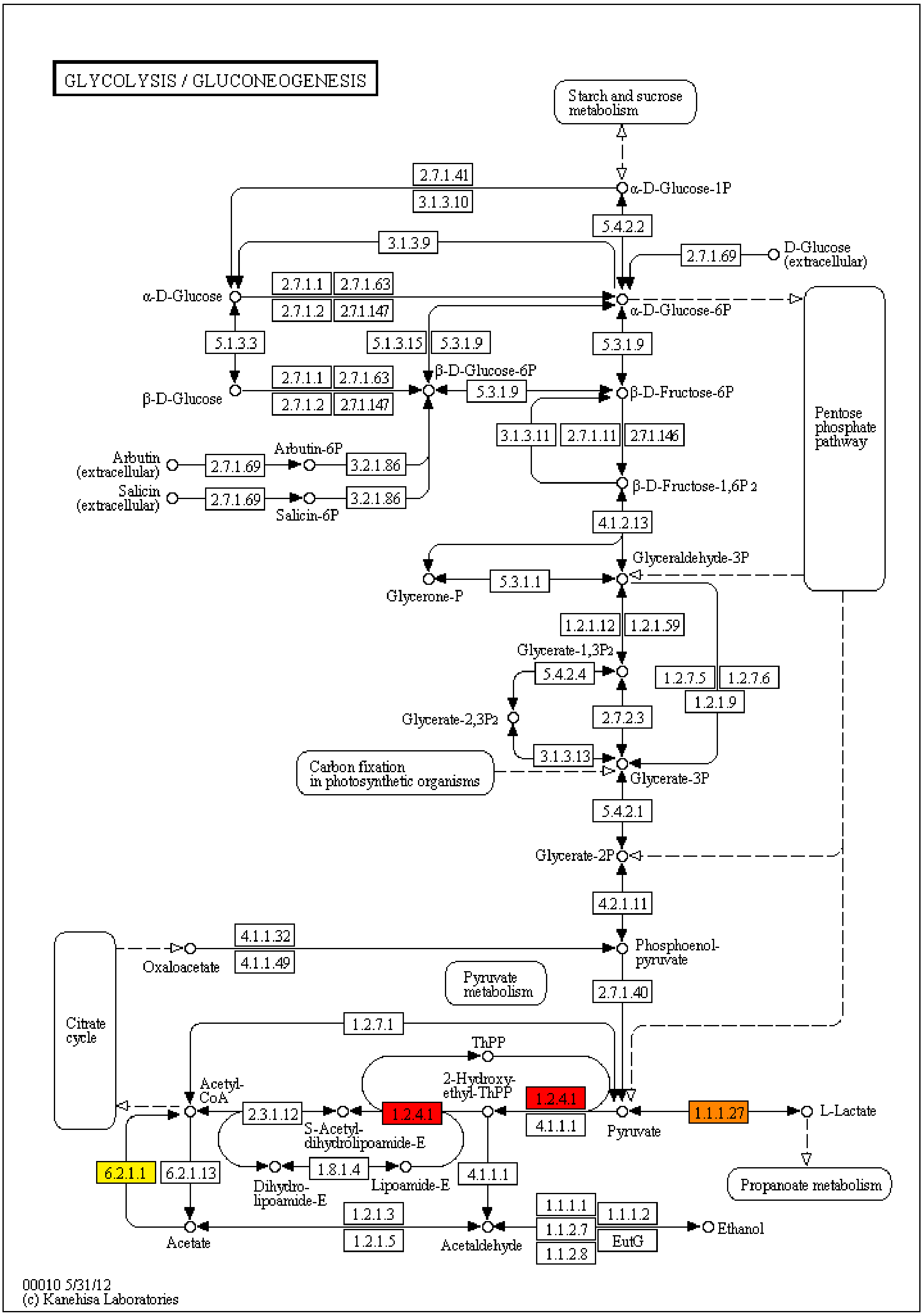
GLYCOLYSIS / GLUCONEOGENESIS
Starch and sucrose metabolism
2.7.1.41
3.1.3.10
α-D-Glucose-1P
5.4.2.2
3.1.3.9
2.7.1.69
D-Glucose (extracellular)
α-D-Glucose
2.7.1.1
2.7.1.63
2.7.1.2
2.7.1.147
α-D-Glucose-6P
5.1.3.3
5.1.3.15
5.3.1.9
5.3.1.9
β-D-Glucose
2.7.1.1
2.7.1.63
2.7.1.2
2.7.1.147
β-D-Glucose-6P
5.3.1.9
β-D-Fructose-6P
Pentose phosphate pathway
3.1.3.11
2.7.1.11
2.7.1.146
Arbutin (extracellular)
2.7.1.69
Arbutin-6P
3.2.1.86
Salicin (extracellular)
2.7.1.69
3.2.1.86
Salicin-6P
β-D-Fructose-1,6P2
4.1.2.13
Glyceraldehyde-3P
5.3.1.1
Glycerone-P
1.2.1.12
1.2.1.59
Glycerate-1,3P2
5.4.2.4
1.2.7.5
1.2.7.6
1.2.1.9
Glycerate-2,3P2
2.7.2.3
3.1.3.13
Carbon fixation in photosynthetic organisms
Glycerate-3P
5.4.2.1
Glycerate-2P
4.2.1.11
4.1.1.32
4.1.1.49
Oxaloacetate
Phosphoenol-pyruvate
Citrate cycle
Pyruvate metabolism
2.7.1.40
1.2.7.1
ThPP
2-Hydroxy-ethyl-ThPP
1.2.4.1
Acetyl-CoA
2.3.1.12
1.2.4.1
4.1.1.1
Pyruvate
1.1.1.27
L-Lactate
S-Acetyl-dihydrolipoamide-E
6.2.1.1
6.2.1.13
Dihydro-lipoamide-E
1.8.1.4
Lipoamide-E
4.1.1.1
Propanoate metabolism
1.2.1.3
1.1.1.1
1.1.1.2
1.1.2.7
Ethanol
1.2.1.5
EutG
1.1.2.8
Acetate
Acetaldehyde
00010 5/31/12
(c) Kanehisa Laboratories

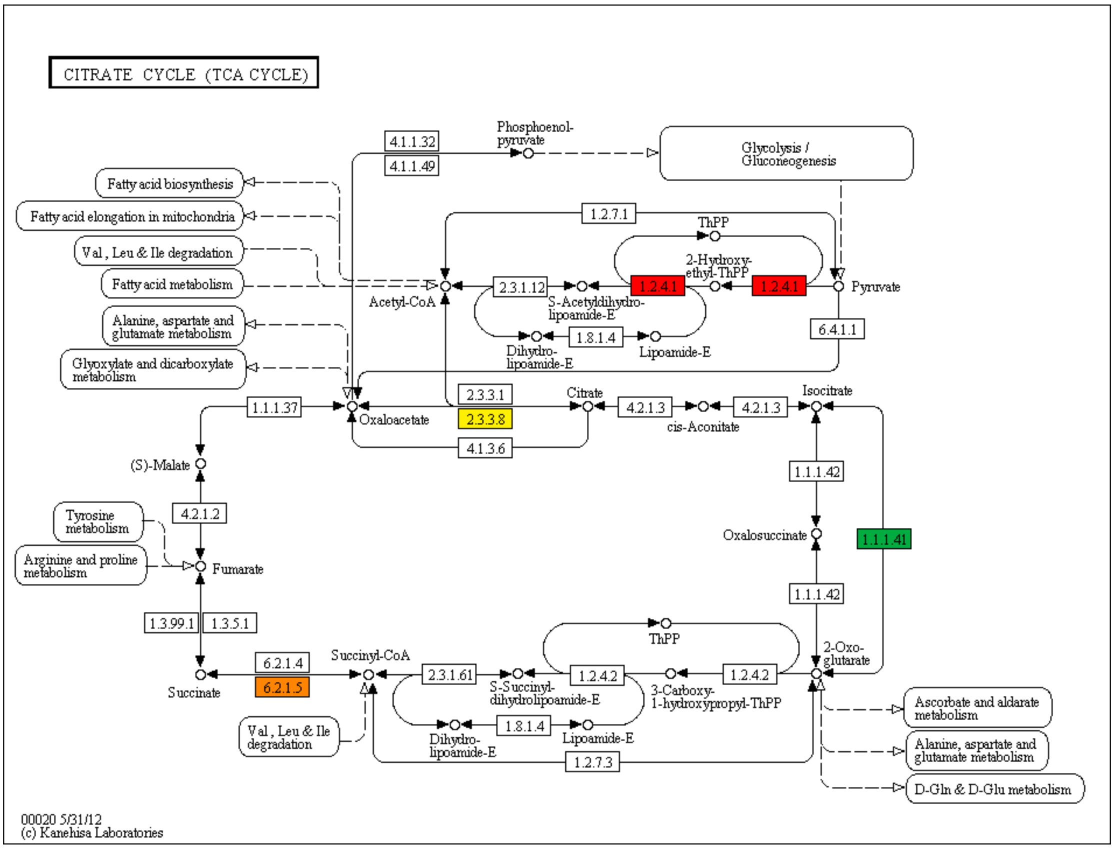
CITRATE CYCLE (TCA CYCLE)
4.1.1.32
4.1.1.49
Phosphoenol-pyruvate
Glycolysis / Gluconeogenesis
Fatty acid biosynthesis
Fatty acid elongation in mitochondria
Val, Leu & Ile degradation
Fatty acid metabolism
Alanine, aspartate and glutamate metabolism
Glyoxylate and dicarboxylate metabolism
1.2.7.1
ThPP
2-Hydroxy-ethyl-ThPP
Acetyl-CoA
2.3.1.12
S-Acetyldihydro-lipoamide-E
1.2.4.1
1.2.4.1
Pyruvate
Dihydro-lipoamide-E
1.8.1.4
Lipoamide-E
6.4.1.1
2.3.3.1
2.3.3.8
Citrate
4.2.1.3
4.2.1.3
Isocitrate
cis-Aconitate
1.1.1.37
Oxaloacetate
4.1.3.6
(S)-Malate
4.2.1.2
Tyrosine metabolism
Arginine and proline metabolism
Fumarate
1.1.1.42
Oxalosuccinate
1.1.1.41
1.1.1.42
1.3.99.1
1.3.5.1
ThPP
Succinyl-CoA
6.2.1.4
6.2.1.5
Succinate
2.3.1.61
S-Succinyl-dihydrolipoamide-E
1.2.4.2
3-Carboxy-1-hydroxypropyl-ThPP
1.2.4.2
2-Oxo-glutarate
Val, Leu & Ile degradation
Dihydro-lipoamide-E
1.8.1.4
Lipoamide-E
1.2.7.3
Ascorbate and aldarate metabolism
Alanine, aspartate and glutamate metabolism
D-Gln & D-Glu metabolism
00020 5/31/12
(c) Kanehisa Laboratories

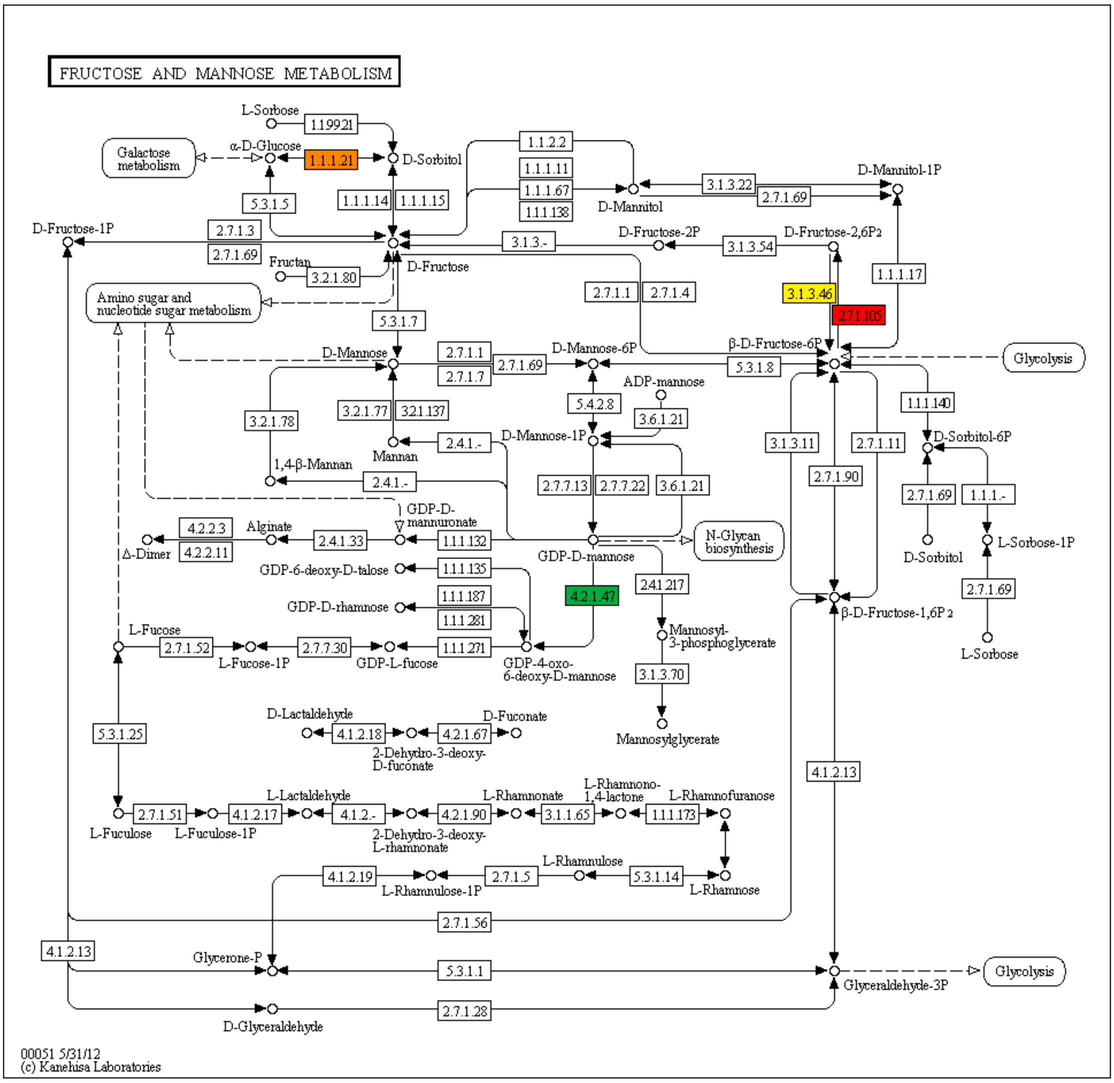
FRUCTOSE AND MANNOSE METABOLISM
L-Sorbose
1.1.99.21
Galactose metabolism
α-D-Glucose
1.1.1.21
D-Sorbitol
1.1.2.2
1.1.1.11
1.1.1.67
1.1.1.138
D-Mannitol
3.1.3.22
2.7.1.69
D-Mannitol-1P
5.3.1.5
1.1.1.14
1.1.1.15
D-Fructose-1P
2.7.1.3
2.7.1.69
3.1.3.-
D-Fructose-2P
3.1.3.54
D-Fructose-2,6P2
1.1.1.17
Fructan
3.2.1.80
D-Fructose
Amino sugar and nucleotide sugar metabolism
2.7.1.1
2.7.1.4
3.1.3.46
2.7.1.105
5.3.1.7
D-Mannose
2.7.1.1
2.7.1.7
2.7.1.69
D-Mannose-6P
β-D-Fructose-6P
5.3.1.8
Glycolysis
ADP-mannose
5.4.2.8
3.6.1.21
1.1.1.140
3.2.1.78
3.2.1.77
3.2.1.137
2.4.1.-
D-Mannose-1P
3.1.3.11
2.7.1.11
D-Sorbitol-6P
Mannan
1,4-β-Mannan
2.4.1.-
2.7.7.13
2.7.7.22
3.6.1.21
2.7.1.90
2.7.1.69
1.1.1.-
GDP-D-mannuronate
4.2.2.3
Alginate
2.4.1.33
1.1.1.132
N-Glycan biosynthesis
Δ-Dimer
4.2.2.11
GDP-D-mannose
D-Sorbitol
L-Sorbose-1P
GDP-6-deoxy-D-talose
1.1.1.135
2.4.1.217
1.1.1.187
4.2.1.47
2.7.1.69
GDP-D-rhamnose
1.1.1.281
β-D-Fructose-1,6P2
Mannosyl-3-phosphoglycerate
L-Fucose
2.7.1.52
2.7.7.30
1.1.1.271
L-Sorbose
L-Fucose-1P
GDP-L-fucose
GDP-4-oxo-6-deoxy-D-mannose
3.1.3.70
D-Lactaldehyde
D-Fuconate
5.3.1.25
4.1.2.18
4.2.1.67
Mannosylglycerate
2-Dehydro-3-deoxy-D-fuconate
4.1.2.13
L-Lactaldehyde
L-Rhamnonate
L-Rhamnono-1,4-lactone
L-Rhamnofuranose
2.7.1.51
4.1.2.17
4.1.2.-
4.2.1.90
3.1.1.65
1.1.1.173
L-Fuculose
L-Fuculose-1P
2-Dehydro-3-deoxy-L-rhamnonate
L-Rhamnulose
4.1.2.19
2.7.1.5
5.3.1.14
L-Rhamnulose-1P
L-Rhamnose
2.7.1.56
4.1.2.13
Glycerone-P
5.3.1.1
Glycolysis
Glyceraldehyde-3P
2.7.1.28
D-Glyceraldehyde
00051 5/31/12
(c) Kanehisa Laboratories

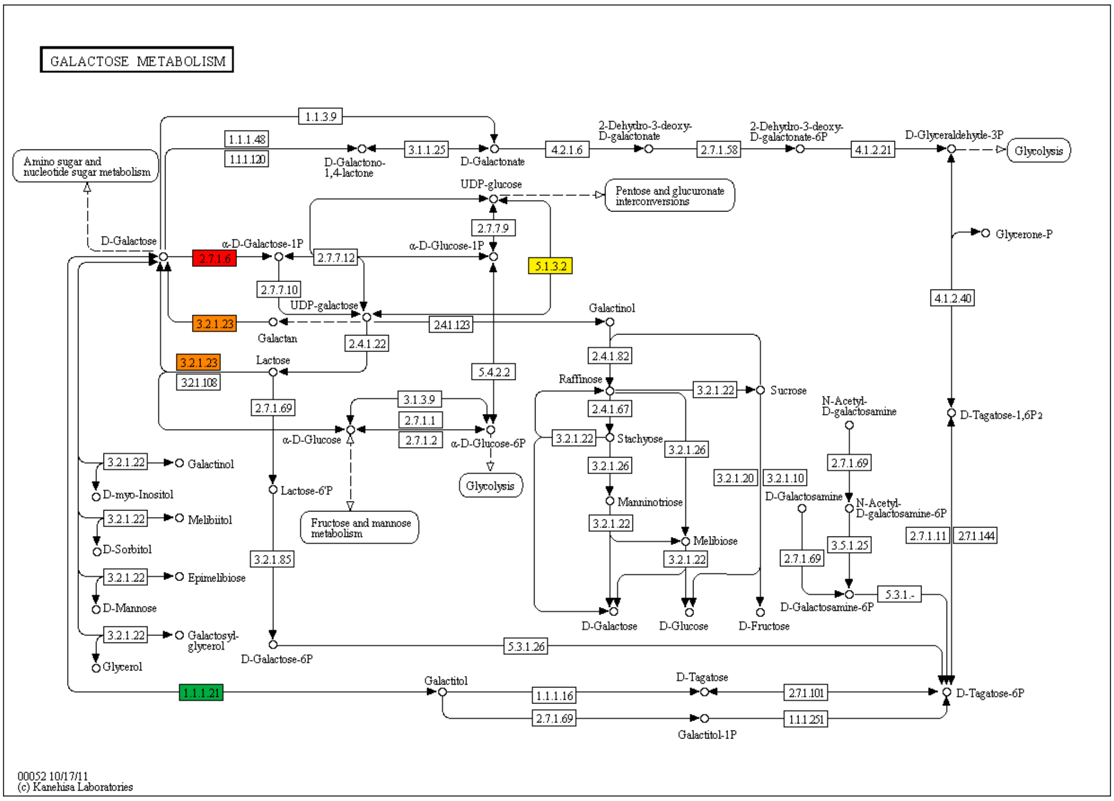
GALACTOSE METABOLISM
Amino sugar and nucleotide sugar metabolism
D-Galactose
1.1.3.9
1.1.1.48
1.1.1.120
D-Galactono-1,4-lactone
3.1.1.25
D-Galactonate
4.2.1.6
2-Dehydro-3-deoxy-D-galactonate
2.7.1.58
2-Dehydro-3-deoxy-D-galactonate-6P
4.1.2.21
D-Glyceraldehyde-3P
Glycolysis
UDP-glucose
Pentose and glucuronate interconversions
2.7.7.9
α-D-Galactose-1P
α-D-Glucose-1P
2.7.1.6
2.7.7.12
5.1.3.2
2.7.7.10
UDP-galactose
Glycerone-P
4.1.2.40
3.2.1.23
Galactan
2.4.1.22
2.4.1.123
Galactinol
Lactose
3.2.1.108
5.4.2.2
2.4.1.82
Raffinose
3.2.1.22
Sucrose
3.1.3.9
2.7.1.69
2.7.1.1
2.7.1.2
α-D-Glucose
α-D-Glucose-6P
2.4.1.67
Stachyose
3.2.1.26
N-Acetyl-D-galactosamine
D-Tagatose-1,6P2
Glycolysis
Lactose-6P
Fructose and mannose metabolism
Manninotriose
3.2.1.20
3.2.1.10
D-Galactosamine
N-Acetyl-D-galactosamine-6P
D-myo-Inositol
Melibiitol
D-Sorbitol
Epimelibiose
D-Mannose
Galactosyl-glycerol
Glycerol
3.2.1.85
Melibiose
3.5.1.25
2.7.1.11
2.7.1.144
5.3.1.-
D-Galactose
D-Glucose
D-Fructose
D-Galactosamine-6P
D-Galactose-6P
5.3.1.26
1.1.1.21
Galactitol
1.1.1.16
D-Tagatose
2.7.1.101
D-Tagatose-6P
2.7.1.69
1.1.1.251
Galactitol-1P
00052 10/17/11
(c) Kanehisa Laboratories

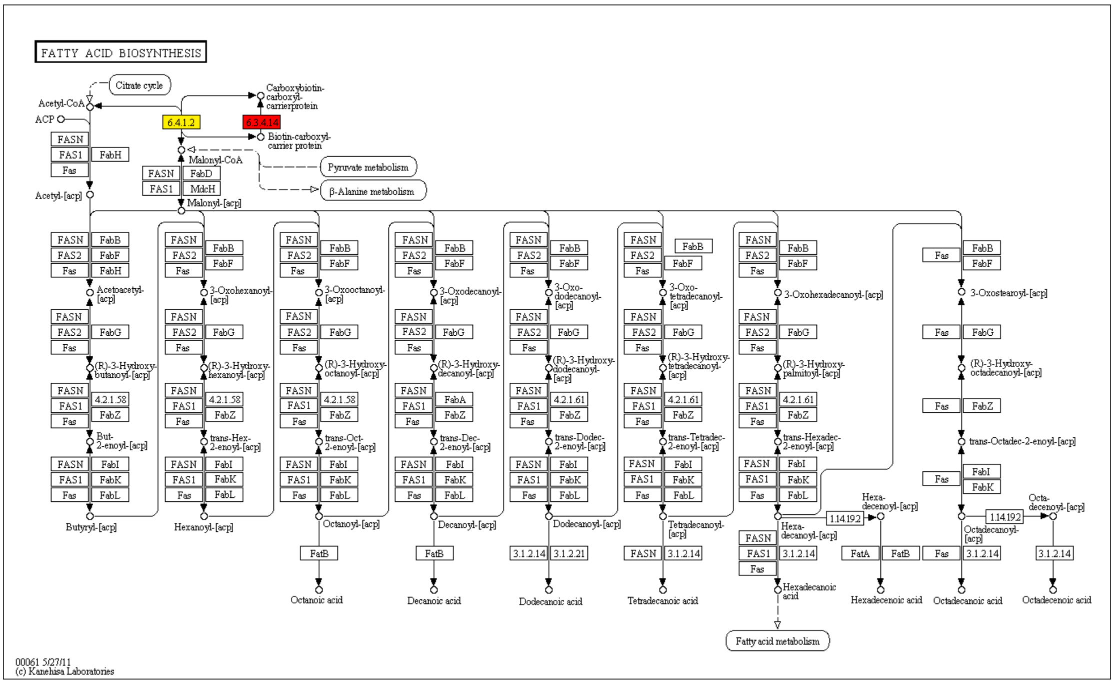
FATTY ACID BIOSYNTHESIS
Citrate cycle
Acetyl-CoA
ACP
Carboxybiotin-carboxyl-carrier protein
6.4.1.2
6.3.4.14
Biotin-carboxyl-carrier protein
FASN
FAS1
FabH
Fas
Malonyl-CoA
FabD
MdcH
Pyruvate metabolism
β-Alanine metabolism
Acetyl-[acp]
Malonyl-[acp]
FAS2
FabB
FabF
FabG
FabZ
FabI
FabK
FabL
FabA
4.2.1.58
4.2.1.61
Acetoacetyl-[acp]
3-Oxohexanoyl-[acp]
3-Oxooctanoyl-[acp]
3-Oxodecanoyl-[acp]
3-Oxo-dodecanoyl-[acp]
3-Oxo-tetradecanoyl-[acp]
3-Oxohexadecanoyl-[acp]
3-Oxostearoyl-[acp]
(R)-3-Hydroxy-butanoyl-[acp]
(R)-3-Hydroxy-hexanoyl-[acp]
(R)-3-Hydroxy-octanoyl-[acp]
(R)-3-Hydroxy-decanoyl-[acp]
(R)-3-Hydroxy-dodecanoyl-[acp]
(R)-3-Hydroxy-tetradecanoyl-[acp]
(R)-3-Hydroxy-palmitoyl-[acp]
(R)-3-Hydroxy-octadecanoyl-[acp]
But-2-enoyl-[acp]
trans-Hex-2-enoyl-[acp]
trans-Oct-2-enoyl-[acp]
trans-Dec-2-enoyl-[acp]
trans-Dodec-2-enoyl-[acp]
trans-Tetradec-2-enoyl-[acp]
trans-Hexadec-2-enoyl-[acp]
trans-Octadec-2-enoyl-[acp]
Butyryl-[acp]
Hexanoyl-[acp]
Octanoyl-[acp]
Decanoyl-[acp]
Dodecanoyl-[acp]
Tetradecanoyl-[acp]
Hexa-decanoyl-[acp]
Hexa-decenoyl-[acp]
1.14.19.2
Octadecanoyl-[acp]
Octa-decenoyl-[acp]
FatB
FatA
3.1.2.14
3.1.2.21
Octanoic acid
Decanoic acid
Dodecanoic acid
Tetradecanoic acid
Hexadecanoic acid
Hexadecenoic acid
Octadecanoic acid
Octadecenoic acid
Fatty acid metabolism
00061 5/27/11
(c) Kanehisa Laboratories

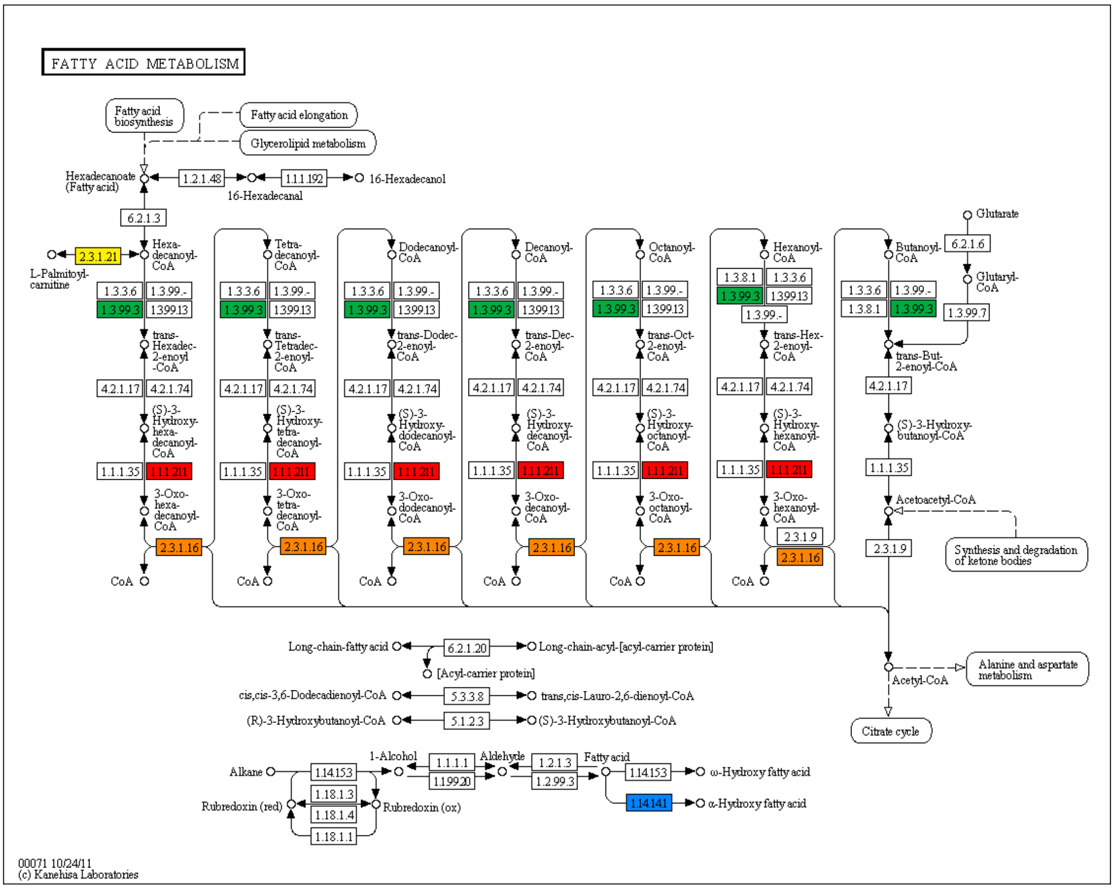
FATTY ACID METABOLISM
Fatty acid biosynthesis
Fatty acid elongation
Glycerolipid metabolism
Hexadecanoate (Fatty acid)
1.2.1.48
1.1.1.192
16-Hexadecanol
16-Hexadecanal
6.2.1.3
2.3.1.21
L-Palmitoyl-carnitine
Hexa-decanoyl-CoA
Tetra-decanoyl-CoA
Dodecanoyl-CoA
Decanoyl-CoA
Octanoyl-CoA
Hexanoyl-CoA
Butanoyl-CoA
Glutarate
6.2.1.6
Glutaryl-CoA
1.3.99.7
1.3.3.6
1.3.99.-
1.3.99.3
1.3.99.13
1.3.8.1
trans-Hexadec-2-enoyl-CoA
trans-Tetradec-2-enoyl-CoA
trans-Dodec-2-enoyl-CoA
trans-Dec-2-enoyl-CoA
trans-Oct-2-enoyl-CoA
trans-Hex-2-enoyl-CoA
trans-But-2-enoyl-CoA
4.2.1.17
4.2.1.74
(S)-3-Hydroxy-hexa-decanoyl-CoA
(S)-3-Hydroxy-tetra-decanoyl-CoA
(S)-3-Hydroxy-dodecanoyl-CoA
(S)-3-Hydroxy-decanoyl-CoA
(S)-3-Hydroxy-octanoyl-CoA
(S)-3-Hydroxy-hexanoyl-CoA
(S)-3-Hydroxy-butanoyl-CoA
1.1.1.35
1.1.1.211
3-Oxo-hexa-decanoyl-CoA
3-Oxo-tetra-decanoyl-CoA
3-Oxo-dodecanoyl-CoA
3-Oxo-decanoyl-CoA
3-Oxo-octanoyl-CoA
3-Oxo-hexanoyl-CoA
Acetoacetyl-CoA
2.3.1.16
2.3.1.9
CoA
Synthesis and degradation of ketone bodies
Acetyl-CoA
Alanine and aspartate metabolism
Citrate cycle
Long-chain-fatty acid
6.2.1.20
Long-chain-acyl-[acyl-carrier protein]
[Acyl-carrier protein]
cis,cis-3,6-Dodecadienoyl-CoA
5.3.3.8
trans,cis-Lauro-2,6-dienoyl-CoA
(R)-3-Hydroxybutanoyl-CoA
5.1.2.3
(S)-3-Hydroxybutanoyl-CoA
Alkane
1.14.15.3
1-Alcohol
1.1.1.1
1.1.99.20
Aldehyde
1.2.1.3
1.2.99.3
Fatty acid
ω-Hydroxy fatty acid
1.14.14.1
α-Hydroxy fatty acid
1.18.1.3
1.18.1.4
1.18.1.1
Rubredoxin (red)
Rubredoxin (ox)
00071 10/24/11
(c) Kanehisa Laboratories

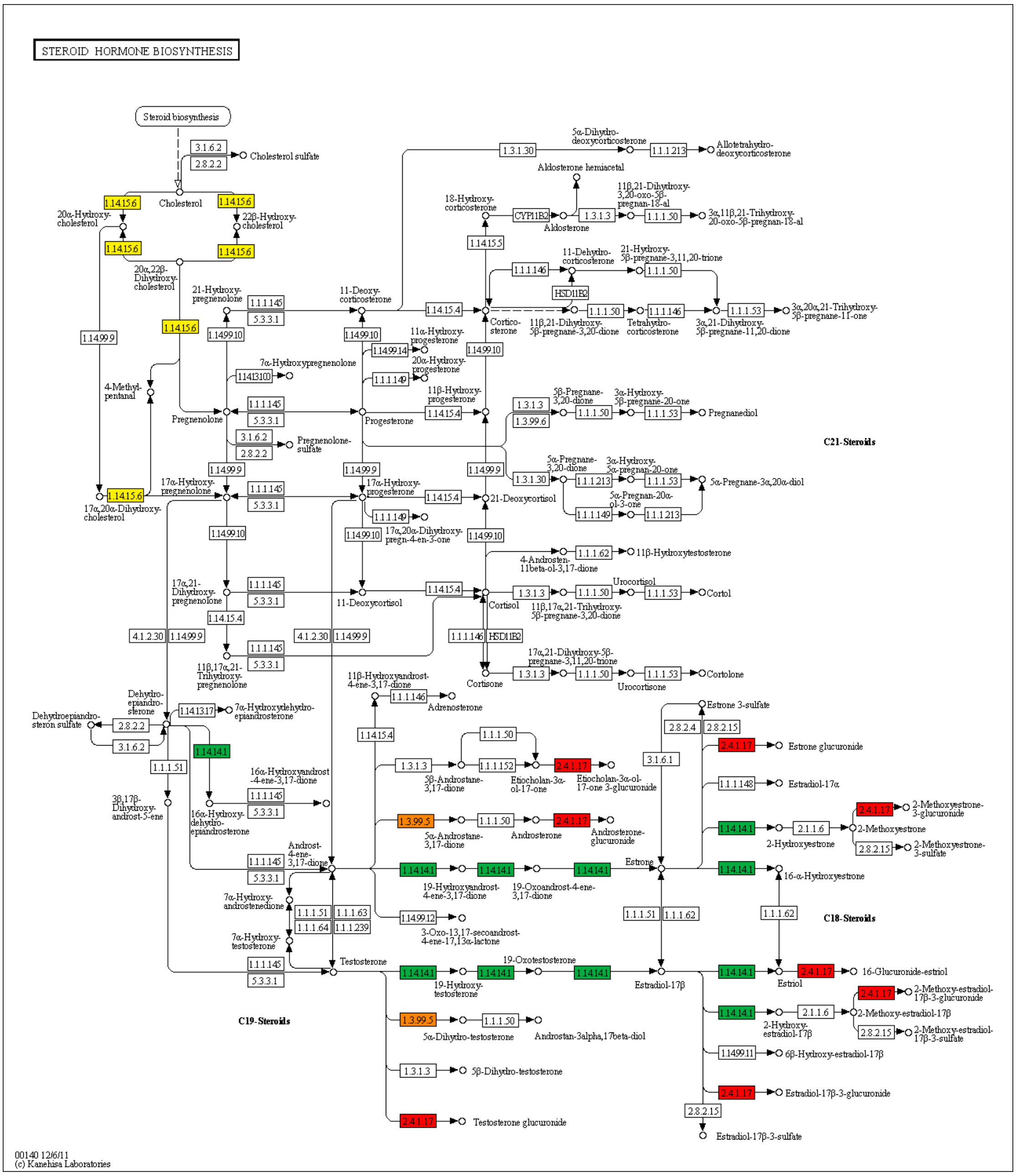
STEROID HORMONE BIOSYNTHESIS
Steroid biosynthesis
3.1.6.2
2.8.2.2
Cholesterol sulfate
1.14.15.6
Cholesterol
20α-Hydroxy-cholesterol
22β-Hydroxy-cholesterol
20α,22β-Dihydroxy-cholesterol
1.14.99.9
4-Methyl-pentanal
17α,20α-Dihydroxy-cholesterol
21-Hydroxy-pregnenolone
1.1.1.145
5.3.3.1
11-Deoxy-corticosterone
1.14.15.4
1.3.1.30
5α-Dihydro-deoxycorticosterone
1.1.1.213
Allotetrahydro-deoxycorticosterone
Aldosterone hemiacetal
18-Hydroxy-corticosterone
CYP11B2
1.3.1.3
Aldosterone
11β,21-Dihydroxy-3,20-oxo-5β-pregnan-18-al
1.1.1.50
3α,11β,21-Trihydroxy-20-oxo-5β-pregnan-18-al
1.14.15.5
11-Dehydro-corticosterone
1.1.1.146
21-Hydroxy-5β-pregnane-3,11,20-trione
HSD11B2
Cortico-sterone
11β,21-Dihydroxy-5β-pregnane-3,20-dione
Tetrahydro-corticosterone
3α,21-Dihydroxy-5β-pregnane-11,20-dione
1.1.1.53
3α,20α,21-Trihydroxy-5β-pregnane-11-one
1.14.99.10
1.14.99.14
11α-Hydroxy-progesterone
20α-Hydroxy-progesterone
7α-Hydroxypregnenolone
1.14.13.100
1.1.1.149
11β-Hydroxy-progesterone
Pregnenolone
Progesterone
5β-Pregnane-3,20-dione
3α-Hydroxy-5β-pregnane-20-one
1.3.99.6
Pregnanediol
C21-Steroids
Pregnenolone-sulfate
5α-Pregnane-3,20-dione
3α-Hydroxy-5α-pregnan-20-one
5α-Pregnane-3α,20α-diol
5α-Pregnan-20α-ol-3-one
17α-Hydroxy-pregnenolone
17α-Hydroxy-progesterone
21-Deoxycortisol
17α,20α-Dihydroxy-pregn-4-en-3-one
4-Androsten-11beta-ol-3,17-dione
1.1.1.62
11β-Hydroxytestosterone
17α,21-Dihydroxy-pregnenolone
11-Deoxycortisol
Cortisol
Urocortisol
11β,17α,21-Trihydroxy-5β-pregnane-3,20-dione
Cortol
4.1.2.30
11β,17α,21-Trihydroxy-pregnenolone
17α,21-Dihydroxy-5β-pregnane-3,11,20-trione
Cortisone
Urocortisone
Cortolone
11β-Hydroxyandrost-4-ene-3,17-dione
Adrenosterone
Dehydro-epiandro-sterone
1.14.13.17
7α-Hydroxydehydro-epiandrosterone
Dehydroepiandro-steron sulfate
1.14.14.1
1.1.1.51
3β,17β-Dihydroxy-androst-5-ene
16α-Hydroxyandrost-4-ene-3,17-dione
16α-Hydroxy-dehydro-epiandrosterone
5β-Androstane-3,17-dione
1.1.1.152
2.4.1.17
Etiocholan-3α-ol-17-one
Etiocholan-3α-ol-17-one 3-glucuronide
1.3.99.5
5α-Androstane-3,17-dione
Androsterone
Androsterone-glucuronide
Androst-4-ene-3,17-dione
19-Hydroxyandrost-4-ene-3,17-dione
19-Oxoandrost-4-ene-3,17-dione
Estrone
Estrone 3-sulfate
2.8.2.4
2.8.2.15
Estrone glucuronide
3.1.6.1
1.1.1.148
Estradiol-17α
2-Hydroxyestrone
2.1.1.6
2-Methoxyestrone
2-Methoxyestrone-3-glucuronide
2-Methoxyestrone-3-sulfate
16-α-Hydroxyestrone
C18-Steroids
7α-Hydroxy-androstenedione
1.1.1.63
1.1.1.64
1.1.1.239
7α-Hydroxy-testosterone
1.14.99.12
3-Oxo-13,17-secoandrost-4-ene-17,13α-lactone
Testosterone
19-Hydroxy-testosterone
19-Oxotestosterone
Estradiol-17β
Estriol
16-Glucuronide-estriol
C19-Steroids
5α-Dihydro-testosterone
Androstan-3alpha,17beta-diol
2-Hydroxy-estradiol-17β
2-Methoxy-estradiol-17β
2-Methoxy-estradiol-17β-3-glucuronide
2-Methoxy-estradiol-17β-3-sulfate
1.14.99.11
6β-Hydroxy-estradiol-17β
5β-Dihydro-testosterone
Estradiol-17β-3-glucuronide
Testosterone glucuronide
Estradiol-17β-3-sulfate
00140 12/6/11
(c) Kanehisa Laboratories

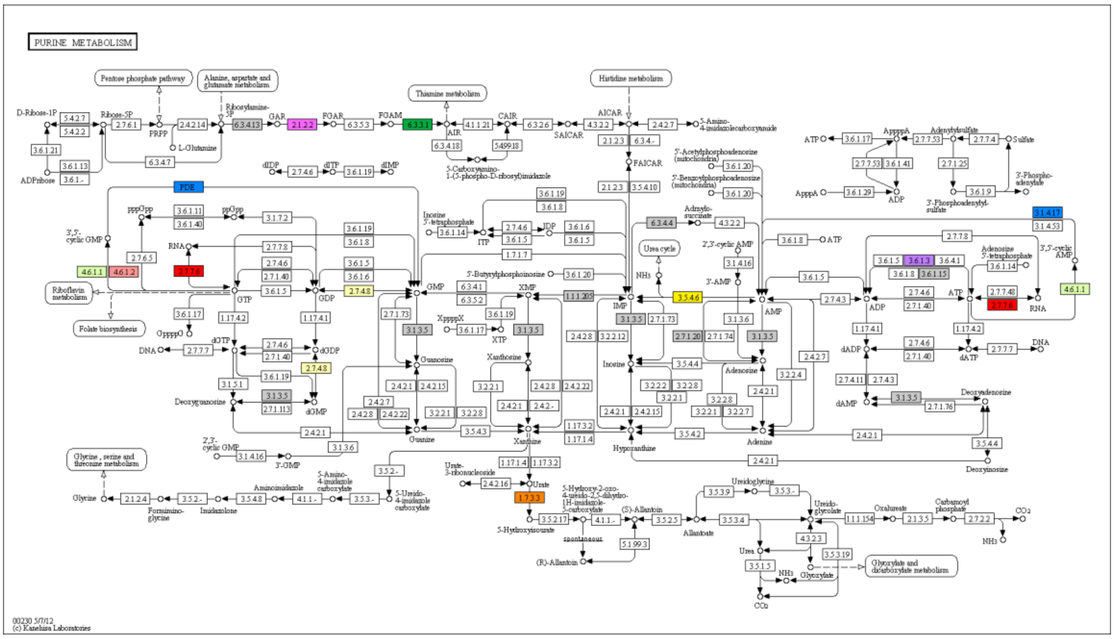
PURINE METABOLISM
Pentose phosphate pathway
Alanine, aspartate and glutamate metabolism
Thiamine metabolism
Histidine metabolism
Riboflavin metabolism
Folate biosynthesis
Glycine, serine and threonine metabolism
Urea cycle
Glyoxylate and dicarboxylate metabolism
00230 5/7/12
(c) Kanehisa Laboratories

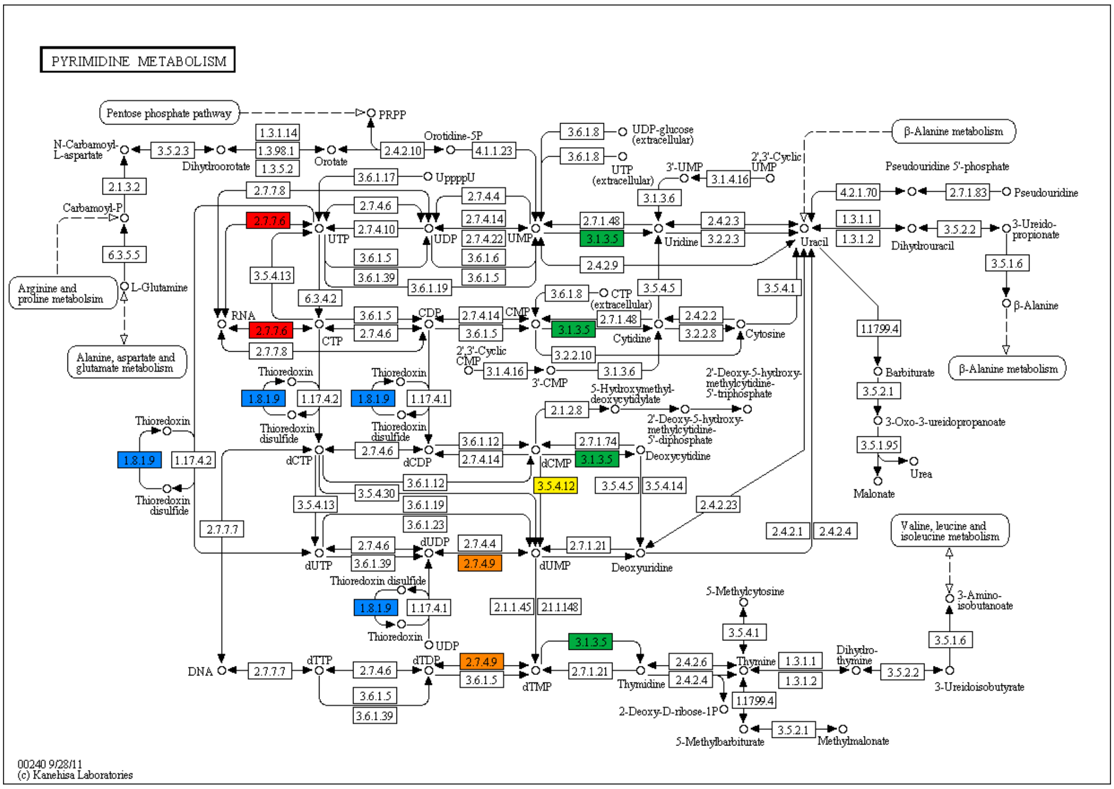
PYRIMIDINE METABOLISM
Pentose phosphate pathway
PRPP
N-Carbamoyl-L-aspartate
Dihydroorotate
Orotate
Orotidine-5P
Carbamoyl-P
L-Glutamine
Arginine and proline metabolsim
Alanine, aspartate and glutamate metabolism
UDP-glucose (extracellular)
UTP (extracellular)
3'-UMP
2',3'-Cyclic UMP
β-Alanine metabolism
Pseudouridine 5'-phosphate
Pseudouridine
UppppU
UTP
UDP
UMP
Uridine
Uracil
Dihydrouracil
3-Ureido-propionate
β-Alanine
RNA
CTP
CDP
CMP
CTP (extracellular)
Cytidine
Cytosine
2',3'-Cyclic CMP
3'-CMP
Thioredoxin
Thioredoxin disulfide
5-Hydroxymethyl-deoxycytidylate
2'-Deoxy-5-hydroxy-methylcytidine-5'-triphosphate
2'-Deoxy-5-hydroxy-methylcytidine-5'-diphosphate
Deoxycytidine
Barbiturate
3-Oxo-3-ureidopropanoate
Urea
Malonate
dCTP
dCDP
dCMP
dUTP
dUDP
dUMP
Deoxyuridine
Valine, leucine and isoleucine metabolism
3-Amino-isobutanoate
5-Methylcytosine
DNA
dTTP
dTDP
dTMP
Thymidine
Thymine
Dihydro-thymine
3-Ureidoisobutyrate
2-Deoxy-D-ribose-1P
5-Methylbarbiturate
Methylmalonate
00240 9/28/11
(c) Kanehisa Laboratories

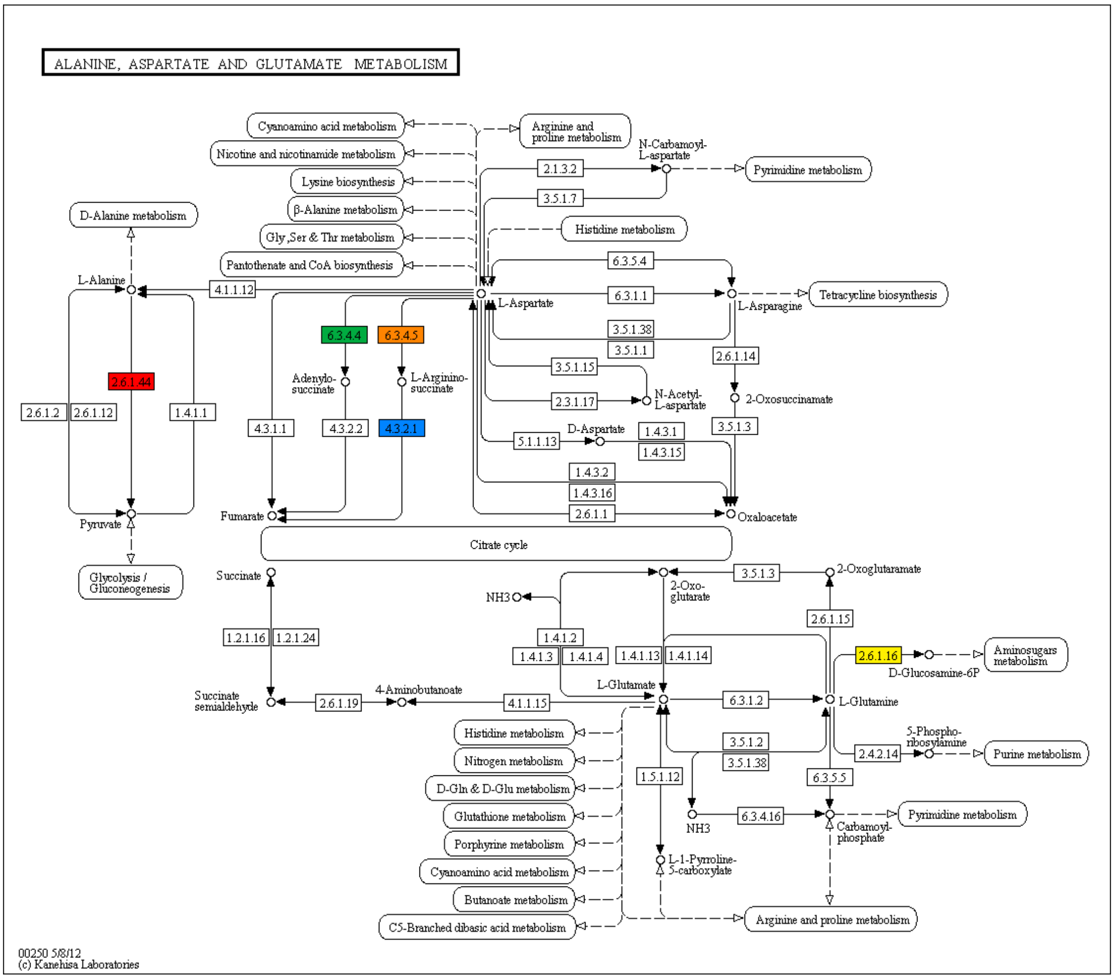
ALANINE, ASPARTATE AND GLUTAMATE METABOLISM
Cyanoamino acid metabolism
Nicotine and nicotinamide metabolism
Lysine biosynthesis
β-Alanine metabolism
Gly,Ser & Thr metabolism
Pantothenate and CoA biosynthesis
Arginine and proline metabolism
N-Carbamoyl-L-aspartate
Pyrimidine metabolism
2.1.3.2
3.5.1.7
Histidine metabolism
D-Alanine metabolism
6.3.5.4
L-Alanine
4.1.1.12
L-Aspartate
6.3.1.1
L-Asparagine
Tetracycline biosynthesis
6.3.4.4
6.3.4.5
3.5.1.38
3.5.1.1
2.6.1.44
Adenylo-succinate
L-Arginino-succinate
3.5.1.15
2.6.1.14
2.6.1.2
2.6.1.12
1.4.1.1
2.3.1.17
N-Acetyl-L-aspartate
2-Oxosuccinamate
4.3.1.1
4.3.2.2
4.3.2.1
D-Aspartate
1.4.3.1
3.5.1.3
5.1.1.13
1.4.3.15
1.4.3.2
1.4.3.16
2.6.1.1
Pyruvate
Fumarate
Oxaloacetate
Citrate cycle
Glycolysis / Gluconeogenesis
Succinate
2-Oxo-glutarate
3.5.1.3
2-Oxoglutaramate
NH3
2.6.1.15
1.2.1.16
1.2.1.24
1.4.1.2
1.4.1.3
1.4.1.4
1.4.1.13
1.4.1.14
2.6.1.16
Aminosugars metabolism
D-Glucosamine-6P
L-Glutamate
Succinate semialdehyde
2.6.1.19
4-Aminobutanoate
4.1.1.15
6.3.1.2
L-Glutamine
Histidine metabolism
Nitrogen metabolism
D-Gln & D-Glu metabolism
Glutathione metabolism
Porphyrine metabolism
Cyanoamino acid metabolism
Butanoate metabolism
C5-Branched dibasic acid metabolism
3.5.1.2
3.5.1.38
5-Phospho-ribosylamine
2.4.2.14
Purine metabolism
1.5.1.12
6.3.5.5
NH3
6.3.4.16
Carbamoyl-phosphate
Pyrimidine metabolism
L-1-Pyrroline-5-carboxylate
Arginine and proline metabolism
00250 5/8/12
(c) Kanehisa Laboratories

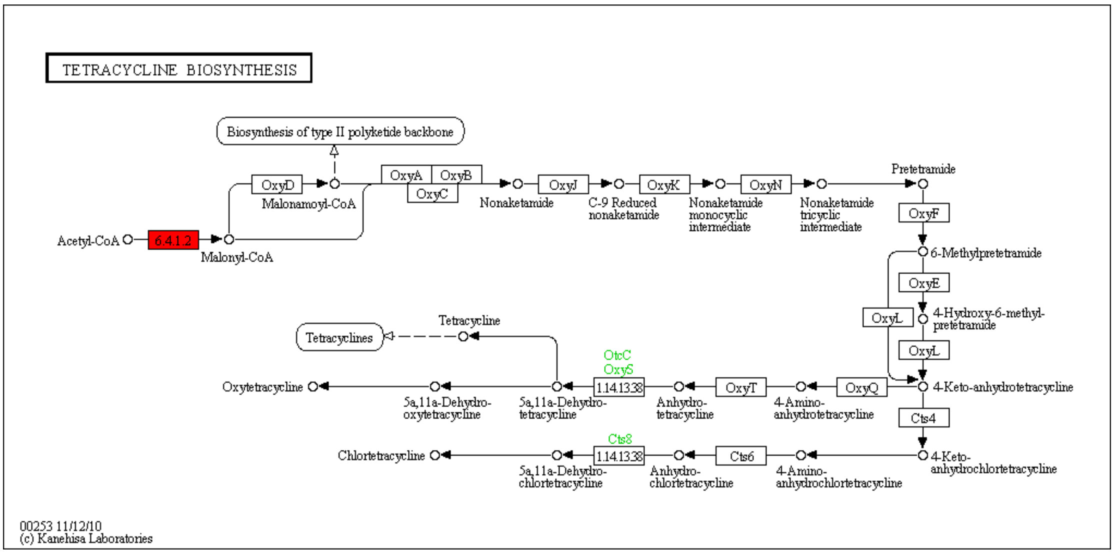
TETRACYCLINE BIOSYNTHESIS
Biosynthesis of type II polyketide backbone
OxyD
Malonamoyl-CoA
OxyA
OxyB
OxyC
OxyJ
OxyK
OxyN
Pretetramide
Acetyl-CoA
6.4.1.2
Malonyl-CoA
Nonaketamide
C-9 Reduced nonaketamide
Nonaketamide monocyclic intermediate
Nonaketamide tricyclic intermediate
OxyF
6-Methylpretetramide
OxyE
OxyL
4-Hydroxy-6-methyl-pretetramide
OxyL
Tetracyclines
Tetracycline
OtcC
OxyS
Oxytetracycline
1.14.13.38
OxyT
OxyQ
4-Keto-anhydrotetracycline
5a,11a-Dehydro-oxytetracycline
5a,11a-Dehydro-tetracycline
Anhydro-tetracycline
4-Amino-anhydrotetracycline
Cts4
Cts8
Chlortetracycline
1.14.13.38
Cts6
4-Keto-anhydrochlortetracycline
5a,11a-Dehydro-chlortetracycline
Anhydro-chlortetracycline
4-Amino-anhydrochlortetracycline
00253 11/12/10
(c) Kanehisa Laboratories

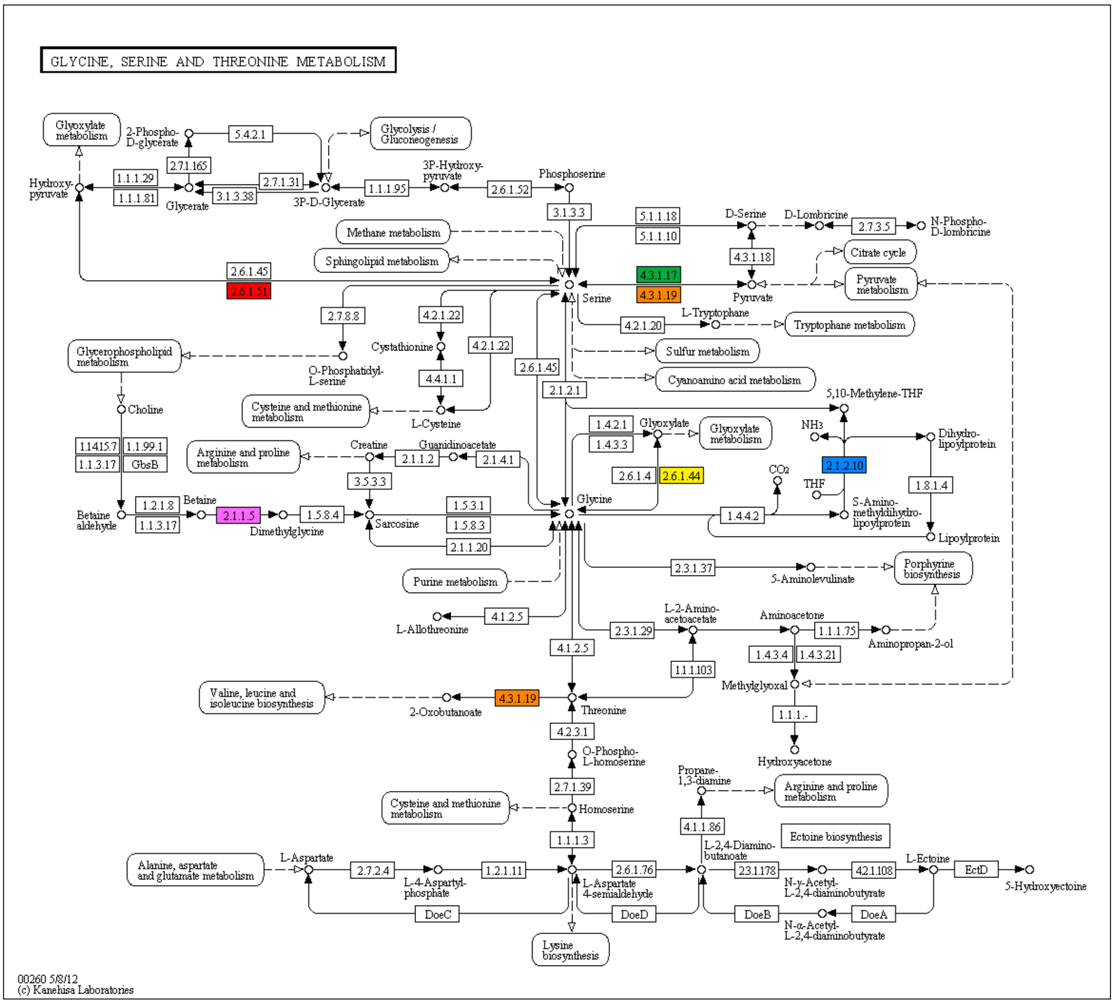
GLYCINE, SERINE AND THREONINE METABOLISM
Glyoxylate metabolism
2-Phospho-D-glycerate
5.4.2.1
Glycolysis / Gluconeogenesis
2.7.1.165
3P-Hydroxy-pyruvate
Phosphoserine
Hydroxy-pyruvate
1.1.1.29
1.1.1.81
Glycerate
3.1.3.38
2.7.1.31
3P-D-Glycerate
1.1.1.95
2.6.1.52
3.1.3.3
5.1.1.18
5.1.1.10
D-Serine
D-Lombricine
2.7.3.5
N-Phospho-D-lombricine
Methane metabolism
Sphingolipid metabolism
4.3.1.18
Citrate cycle
2.6.1.45
2.6.1.51
4.3.1.17
4.3.1.19
Serine
Pyruvate
Pyruvate metabolism
2.7.8.8
4.2.1.22
4.2.1.20
L-Tryptophan
Tryptophan metabolism
Cystathionine
4.2.1.22
Sulfur metabolism
Glycerophospholipid metabolism
O-Phosphatidyl-L-serine
4.4.1.1
2.6.1.45
Cyanoamino acid metabolism
2.1.2.1
5,10-Methylene-THF
Choline
Cysteine and methionine metabolism
L-Cysteine
1.4.2.1
Glyoxylate
Glyoxylate metabolism
NH3
Dihydro-lipoylprotein
1.1.4.15.7
1.1.99.1
1.4.3.3
1.1.3.17
GbsB
Arginine and proline metabolism
Creatine
Guanidinoacetate
2.1.1.2
2.1.4.1
2.6.1.4
2.6.1.44
CO2
2.1.2.10
3.5.3.3
THF
1.8.1.4
Betaine aldehyde
1.2.1.8
1.1.3.17
Betaine
2.1.1.5
1.5.8.4
Dimethylglycine
Sarcosine
1.5.3.1
1.5.8.3
2.1.1.20
Glycine
1.4.4.2
S-Amino-methyldihydro-lipoylprotein
Lipoylprotein
2.3.1.37
5-Aminolevulinate
Porphyrine biosynthesis
Purine metabolism
L-Allothreonine
4.1.2.5
L-2-Amino-acetoacetate
Aminoacetone
2.3.1.29
1.1.1.75
Aminopropan-2-ol
4.1.2.5
1.4.3.4
1.4.3.21
1.1.1.103
Methylglyoxal
Valine, leucine and isoleucine biosynthesis
2-Oxobutanoate
4.3.1.19
Threonine
1.1.1.-
4.2.3.1
O-Phospho-L-homoserine
Hydroxyacetone
Propane-1,3-diamine
2.7.1.39
Arginine and proline metabolism
Cysteine and methionine metabolism
Homoserine
4.1.1.86
Ectoine biosynthesis
1.1.1.3
L-2,4-Diamino-butanoate
Alanine, aspartate and glutamate metabolism
L-Aspartate
2.7.2.4
L-4-Aspartyl-phosphate
1.2.1.11
L-Aspartate 4-semialdehyde
2.6.1.76
2.3.1.178
N-γ-Acetyl-L-2,4-diaminobutyrate
4.2.1.108
L-Ectoine
EctD
5-Hydroxyectoine
DoeC
DoeD
DoeB
DoeA
N-α-Acetyl-L-2,4-diaminobutyrate
Lysine biosynthesis
00260 5/8/12
(c) Kanehisa Laboratories

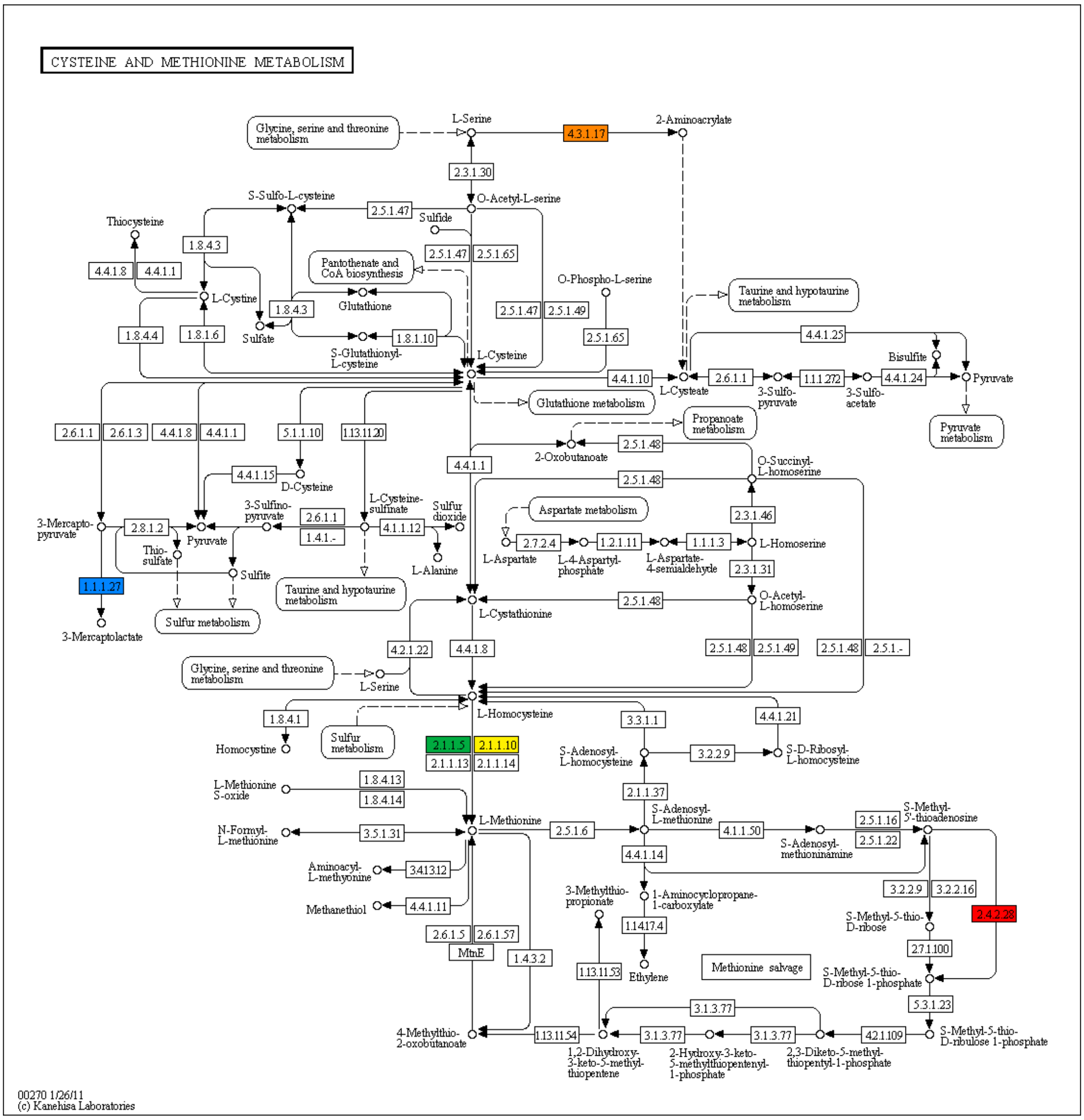
CYSTEINE AND METHIONINE METABOLISM
Glycine, serine and threonine metabolism
L-Serine
4.3.1.17
2-Aminoacrylate
2.3.1.30
O-Acetyl-L-serine
S-Sulfo-L-cysteine
2.5.1.47
Sulfide
Thiocysteine
1.8.4.3
4.4.1.8
4.4.1.1
2.5.1.47
2.5.1.65
Pantothenate and CoA biosynthesis
O-Phospho-L-serine
L-Cystine
1.8.4.3
Glutathione
Taurine and hypotaurine metabolism
2.5.1.47
2.5.1.49
1.8.4.4
1.8.1.6
Sulfate
1.8.1.10
S-Glutathionyl-L-cysteine
L-Cysteine
2.5.1.65
4.4.1.25
Bisulfite
4.4.1.10
L-Cysteate
2.6.1.1
1.1.1.272
4.4.1.24
Pyruvate
3-Sulfo-pyruvate
3-Sulfo-acetate
Glutathione metabolism
Propanoate metabolism
Pyruvate metabolism
2.6.1.1
2.6.1.3
4.4.1.8
4.4.1.1
5.1.1.10
1.13.11.20
2.5.1.48
2-Oxobutanoate
4.4.1.1
4.4.1.15
D-Cysteine
O-Succinyl-L-homoserine
2.5.1.48
L-Cysteine-sulfinate
Sulfur dioxide
Aspartate metabolism
3-Mercapto-pyruvate
2.8.1.2
3-Sulfino-pyruvate
2.6.1.1
4.1.1.12
1.4.1.-
Pyruvate
2.3.1.46
2.7.2.4
1.2.1.11
1.1.1.3
L-Homoserine
Thio-sulfate
L-Alanine
L-Aspartate
L-4-Aspartyl-phosphate
L-Aspartate-4-semialdehyde
Sulfite
2.3.1.31
1.1.1.27
Taurine and hypotaurine metabolism
2.5.1.48
O-Acetyl-L-homoserine
Sulfur metabolism
L-Cystathionine
3-Mercaptolactate
4.2.1.22
4.4.1.8
2.5.1.48
2.5.1.49
2.5.1.48
2.5.1.-
Glycine, serine and threonine metabolism
L-Serine
L-Homocysteine
1.8.4.1
3.3.1.1
4.4.1.21
Sulfur metabolism
Homocystine
2.1.1.5
2.1.1.10
2.1.1.13
2.1.1.14
S-Adenosyl-L-homocysteine
3.2.2.9
S-D-Ribosyl-L-homocysteine
L-Methionine S-oxide
1.8.4.13
1.8.4.14
2.1.1.37
S-Adenosyl-L-methionine
S-Methyl-5'-thioadenosine
N-Formyl-L-methionine
3.5.1.31
L-Methionine
2.5.1.6
4.1.1.50
2.5.1.16
S-Adenosyl-methioninamine
2.5.1.22
Aminoacyl-L-methyonine
3.4.13.12
4.4.1.14
3-Methylthio-propionate
1-Aminocyclopropane-1-carboxylate
3.2.2.9
3.2.2.16
Methanethiol
4.4.1.11
S-Methyl-5-thio-D-ribose
2.4.2.28
1.14.17.4
2.6.1.5
2.6.1.57
MtnE
2.7.1.100
1.4.3.2
Methionine salvage
1.13.11.53
Ethylene
S-Methyl-5-thio-D-ribose 1-phosphate
3.1.3.77
5.3.1.23
4-Methylthio-2-oxobutanoate
1.13.11.54
3.1.3.77
3.1.3.77
4.2.1.109
S-Methyl-5-thio-D-ribulose 1-phosphate
1,2-Dihydroxy-3-keto-5-methyl-thiopentene
2-Hydroxy-3-keto-5-methylthiopentenyl-1-phosphate
2,3-Diketo-5-methyl-thiopentyl-1-phosphate
00270 1/26/11
(c) Kanehisa Laboratories

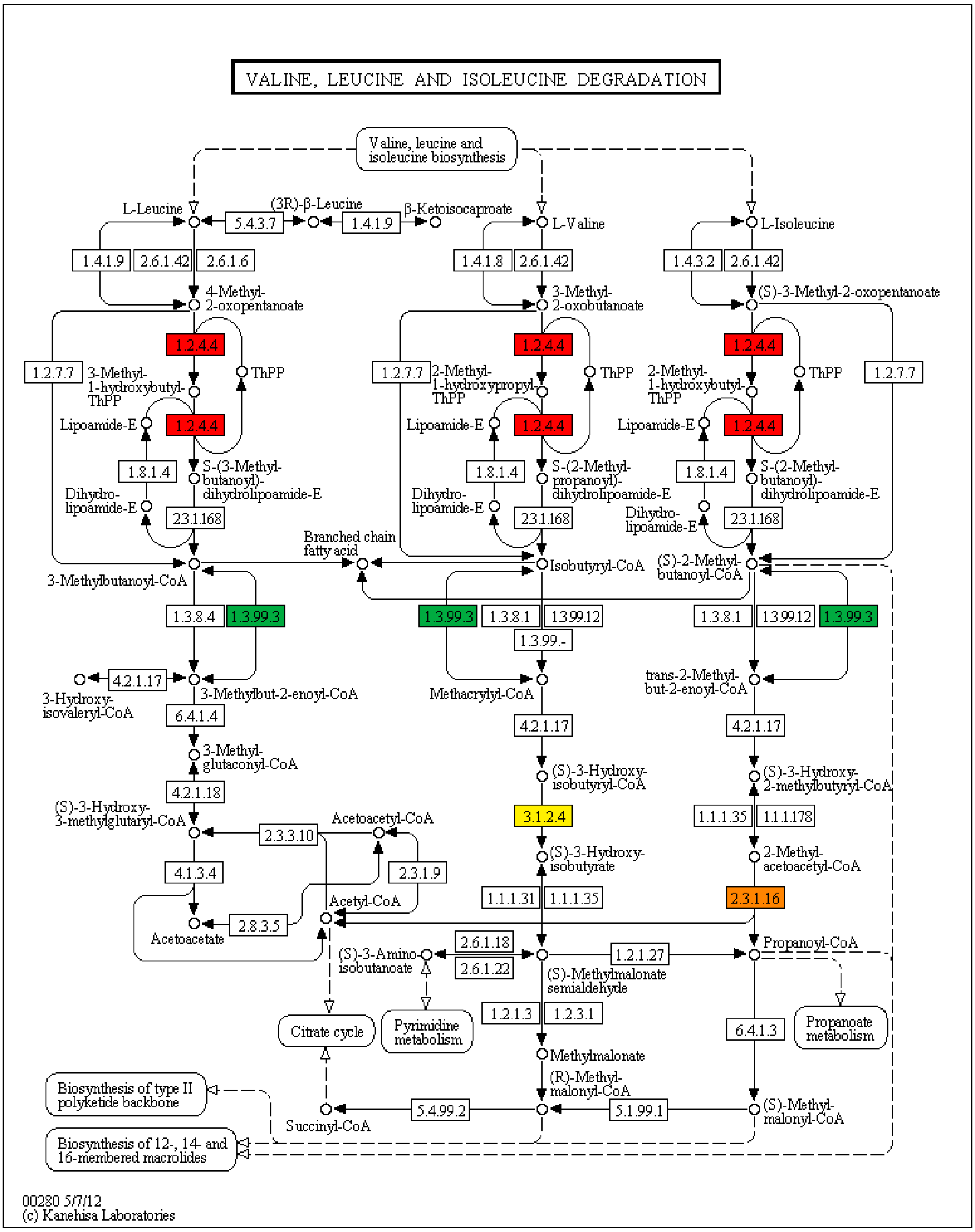
VALINE, LEUCINE AND ISOLEUCINE DEGRADATION
Valine, leucine and isoleucine biosynthesis
L-Leucine
(3R)-β-Leucine
β-Ketoisocaproate
L-Valine
L-Isoleucine
5.4.3.7
1.4.1.9
1.4.1.9
2.6.1.42
2.6.1.6
1.4.1.8
2.6.1.42
1.4.3.2
2.6.1.42
4-Methyl-2-oxopentanoate
3-Methyl-2-oxobutanoate
(S)-3-Methyl-2-oxopentanoate
1.2.4.4
ThPP
1.2.7.7
3-Methyl-1-hydroxybutyl-ThPP
2-Methyl-1-hydroxypropyl-ThPP
2-Methyl-1-hydroxybutyl-ThPP
Lipoamide-E
1.8.1.4
S-(3-Methylbutanoyl)-dihydrolipoamide-E
S-(2-Methylpropanoyl)-dihydrolipoamide-E
S-(2-Methylbutanoyl)-dihydrolipoamide-E
Dihydrolipoamide-E
2.3.1.168
Branched chain fatty acid
3-Methylbutanoyl-CoA
Isobutyryl-CoA
(S)-2-Methylbutanoyl-CoA
1.3.8.4
1.3.99.3
1.3.8.1
1.3.99.12
1.3.99.-
4.2.1.17
3-Hydroxy-isovaleryl-CoA
3-Methylbut-2-enoyl-CoA
Methacrylyl-CoA
trans-2-Methyl-but-2-enoyl-CoA
6.4.1.4
3-Methyl-glutaconyl-CoA
4.2.1.18
(S)-3-Hydroxy-3-methylglutaryl-CoA
(S)-3-Hydroxy-isobutyryl-CoA
(S)-3-Hydroxy-2-methylbutyryl-CoA
3.1.2.4
1.1.1.35
1.1.1.178
2.3.3.10
Acetoacetyl-CoA
2.3.1.9
(S)-3-Hydroxy-isobutyrate
2-Methyl-acetoacetyl-CoA
4.1.3.4
Acetyl-CoA
1.1.1.31
2.3.1.16
Acetoacetate
2.8.3.5
2.6.1.18
2.6.1.22
(S)-3-Amino-isobutanoate
1.2.1.27
Propanoyl-CoA
(S)-Methylmalonate semialdehyde
Citrate cycle
Pyrimidine metabolism
1.2.1.3
1.2.3.1
6.4.1.3
Propanoate metabolism
Methylmalonate
(R)-Methyl-malonyl-CoA
Biosynthesis of type II polyketide backbone
5.4.99.2
5.1.99.1
(S)-Methyl-malonyl-CoA
Succinyl-CoA
Biosynthesis of 12-, 14- and 16-membered macrolides
00280 5/7/12
(c) Kanehisa Laboratories

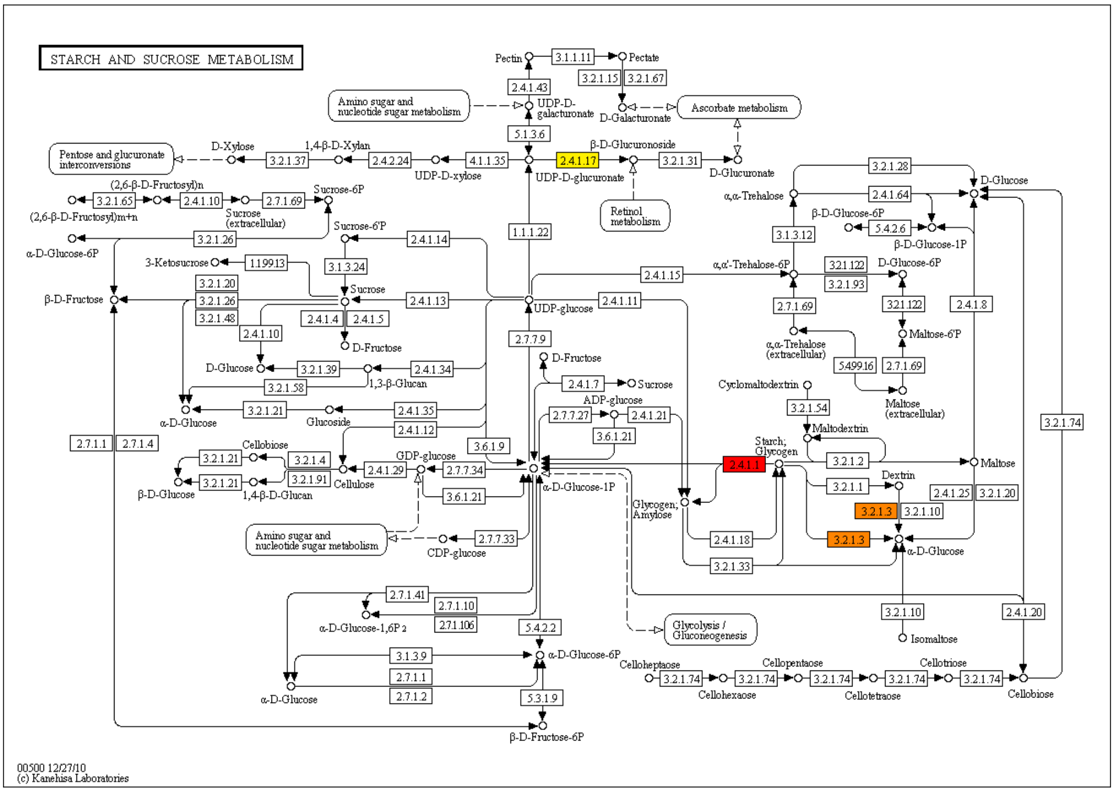
STARCH AND SUCROSE METABOLISM
Pectin
3.1.1.11
Pectate
2.4.1.43
3.2.1.15
3.2.1.67
Amino sugar and nucleotide sugar metabolism
UDP-D-galacturonate
D-Galacturonate
Ascorbate metabolism
5.1.3.6
Pentose and glucuronate interconversions
D-Xylose
1,4-β-D-Xylan
3.2.1.37
2.4.2.24
4.1.1.35
2.4.1.17
β-D-Glucuronoside
3.2.1.31
UDP-D-xylose
UDP-D-glucuronate
D-Glucuronate
(2,6-β-D-Fructosyl)n
3.2.1.65
2.4.1.10
2.7.1.69
Sucrose-6P
Sucrose (extracellular)
(2,6-β-D-Fructosyl)m+n
Sucrose-6'P
2.4.1.14
1.1.1.22
Retinol metabolism
3.2.1.28
D-Glucose
α,α-Trehalose
2.4.1.64
β-D-Glucose-6P
5.4.2.6
3.1.3.12
β-D-Glucose-1P
α-D-Glucose-6P
3.2.1.26
3-Ketosucrose
1.1.99.13
3.1.3.24
α,α'-Trehalose-6P
2.4.1.15
3.2.1.122
D-Glucose-6P
3.2.1.93
Sucrose
3.2.1.20
β-D-Fructose
3.2.1.26
2.4.1.13
2.4.1.11
UDP-glucose
3.2.1.48
2.4.1.4
2.4.1.5
2.7.1.69
3.2.1.122
2.4.1.8
2.4.1.10
Maltose-6'P
D-Fructose
2.7.7.9
α,α-Trehalose (extracellular)
D-Fructose
D-Glucose
3.2.1.39
2.4.1.34
5.4.99.16
2.7.1.69
1,3-β-Glucan
2.4.1.7
Sucrose
Cyclomaltodextrin
3.2.1.58
ADP-glucose
Maltose (extracellular)
3.2.1.21
2.4.1.35
2.7.7.27
2.4.1.21
3.2.1.54
α-D-Glucose
Glucoside
3.2.1.74
2.4.1.12
3.6.1.21
Maltodextrin
2.7.1.1
2.7.1.4
3.6.1.9
Starch; Glycogen
Cellobiose
GDP-glucose
2.4.1.1
3.2.1.2
Maltose
3.2.1.21
3.2.1.4
2.4.1.29
2.7.7.34
β-D-Glucose
3.2.1.21
3.2.1.91
Cellulose
α-D-Glucose-1P
3.2.1.1
Dextrin
1,4-β-D-Glucan
3.6.1.21
Glycogen; Amylose
2.4.1.25
3.2.1.20
3.2.1.3
3.2.1.10
Amino sugar and nucleotide sugar metabolism
2.7.7.33
CDP-glucose
2.4.1.18
3.2.1.3
α-D-Glucose
3.2.1.33
2.7.1.41
2.7.1.10
3.2.1.10
2.4.1.20
α-D-Glucose-1,6P2
2.7.1.106
5.4.2.2
Glycolysis / Gluconeogenesis
Isomaltose
3.1.3.9
α-D-Glucose-6P
Celloheptaose
Cellopentaose
Cellotriose
2.7.1.1
3.2.1.74
3.2.1.74
3.2.1.74
3.2.1.74
3.2.1.74
α-D-Glucose
2.7.1.2
5.3.1.9
Cellohexaose
Cellotetraose
Cellobiose
β-D-Fructose-6P
00500 12/27/10
(c) Kanehisa Laboratories

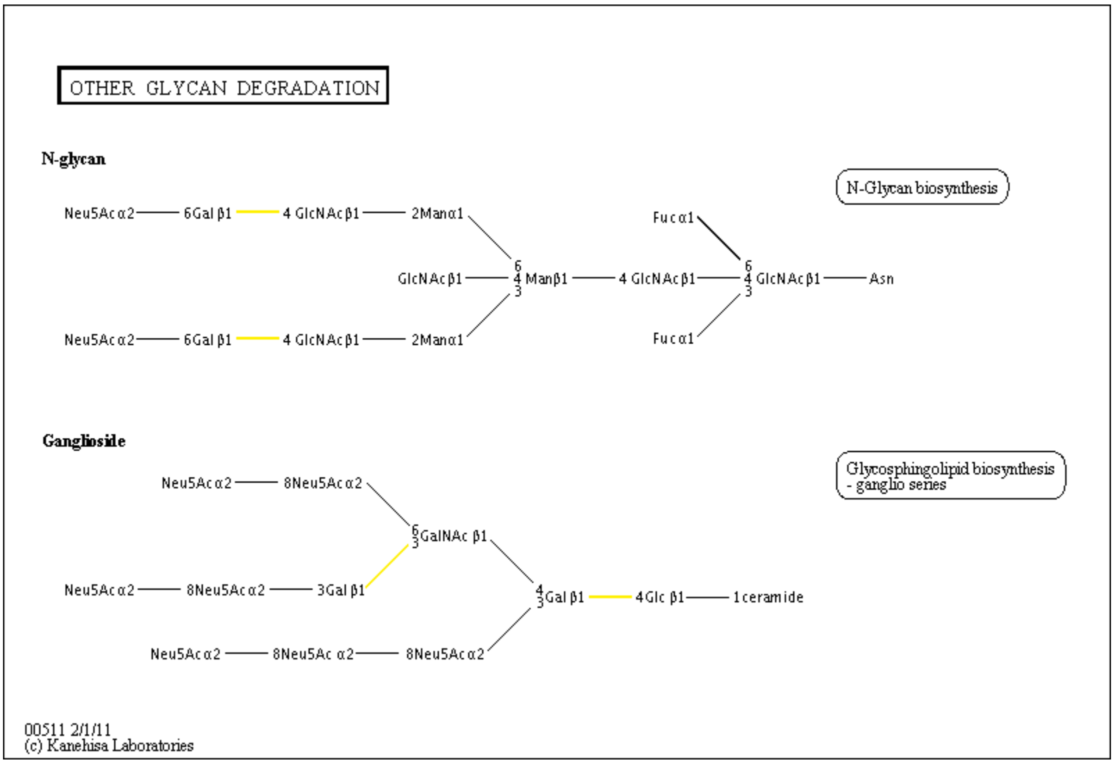
OTHER GLYCAN DEGRADATION
N-glycan
N-Glycan biosynthesis
Neu5Acα2 — 6Galβ1 — 4GlcNAcβ1 — 2Manα1
Fucα1
GlcNAcβ1 — 6 4 3 Manβ1 — 4GlcNAcβ1 — 6 4 3 GlcNAcβ1 — Asn
Neu5Acα2 — 6Galβ1 — 4GlcNAcβ1 — 2Manα1
Fucα1
Ganglioside
Glycosphingolipid biosynthesis - ganglio series
Neu5Acα2 — 8Neu5Acα2
6 3 GalNAc β1
Neu5Acα2 — 8Neu5Acα2 — 3Gal β1
4 3 Gal β1 — 4Glc β1 — 1ceramide
Neu5Acα2 — 8Neu5Ac α2 — 8Neu5Acα2
00511 2/1/11
(c) Kanehisa Laboratories

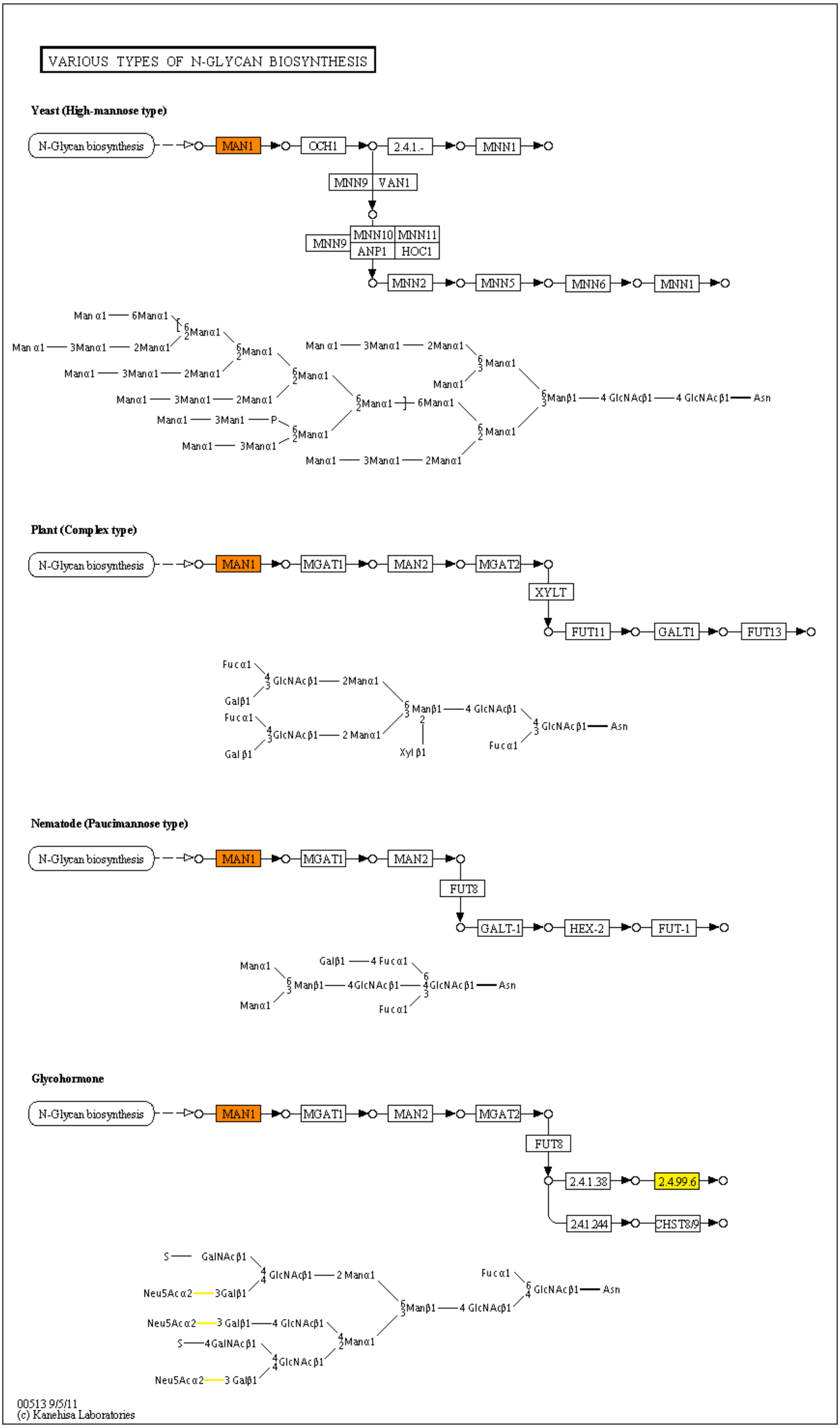
VARIOUS TYPES OF N-GLYCAN BIOSYNTHESIS
Yeast (High-mannose type)
N-Glycan biosynthesis
MAN1
OCH1
2.4.1.-
MNN1
MNN9
VAN1
MNN9
MNN10
MNN11
ANP1
HOC1
MNN2
MNN5
MNN6
MNN1
Plant (Complex type)
N-Glycan biosynthesis
MAN1
MGAT1
MAN2
MGAT2
XYLT
FUT11
GALT1
FUT13
Nematode (Paucimannose type)
N-Glycan biosynthesis
MAN1
MGAT1
MAN2
FUT8
GALT-1
HEX-2
FUT-1
Glycohormone
N-Glycan biosynthesis
MAN1
MGAT1
MAN2
MGAT2
FUT8
2.4.1.38
2.4.99.6
2.4.1.244
CHST8/9
00513 9/5/11
(c) Kanehisa Laboratories

OTHER TYPES OF O-GLYCAN BIOSYNTHESIS

O-linked GlcNAc type

GlcNAc β1 —— Ser/Thr

O-linked Man type

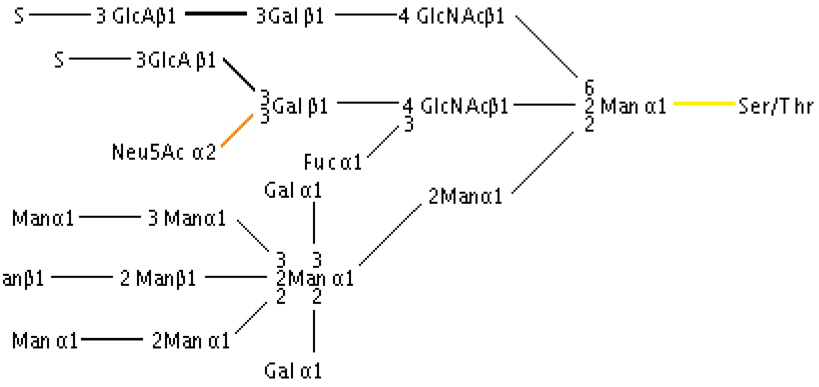

O-linked Fuc type

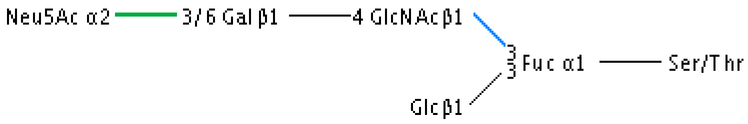

O-linked Glc type

Xyl α1 —— 3 Xyl α1 —— 3 Glc β1 —— Ser

O-linked Gal type

Glc α1 —— 2 Galβ1 —— Hydroxylysine

00514 1/24/11
(c) Kanehisa Laboratories

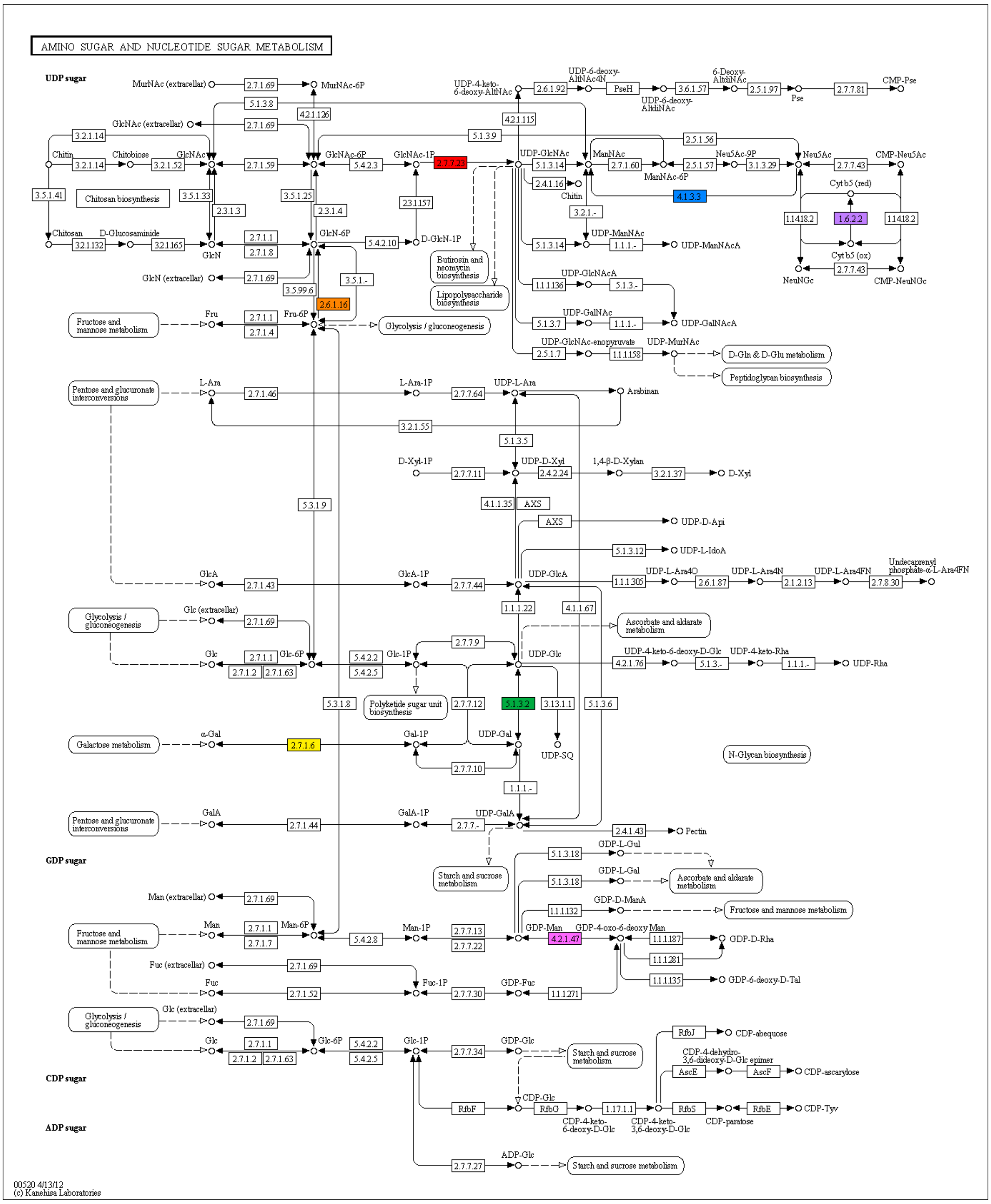
AMINO SUGAR AND NUCLEOTIDE SUGAR METABOLISM
UDP sugar
GDP sugar
CDP sugar
ADP sugar
MurNAc (extracellular)
MurNAc-6P
GlcNAc (extracellular)
Chitin
Chitobiose
GlcNAc
GlcNAc-6P
GlcNAc-1P
UDP-GlcNAc
ManNAc
ManNAc-6P
Neu5Ac-9P
Neu5Ac
CMP-Neu5Ac
UDP-4-keto-6-deoxy-AltNAc
UDP-6-deoxy-AltNAc4N
UDP-6-deoxy-AltdiNAc
6-Deoxy-AltdiNAc
Pse
CMP-Pse
Chitosan biosynthesis
Chitosan
D-Glucosaminide
GlcN
GlcN-6P
D-GlcN-1P
GlcN (extracellular)
Butirosin and neomycin biosynthesis
Lipopolysaccharide biosynthesis
UDP-ManNAc
UDP-ManNAcA
UDP-GlcNAcA
UDP-GalNAc
UDP-GalNAcA
UDP-GlcNAc-enolpyruvate
UDP-MurNAc
D-Gln & D-Glu metabolism
Peptidoglycan biosynthesis
Cyt b5 (red)
Cyt b5 (ox)
NeuNGc
CMP-NeuNGc
Fructose and mannose metabolism
Fru
Fru-6P
Glycolysis / gluconeogenesis
Pentose and glucuronate interconversions
L-Ara
L-Ara-1P
UDP-L-Ara
Arabinan
D-Xyl-1P
UDP-D-Xyl
1,4-β-D-Xylan
D-Xyl
UDP-D-Api
UDP-L-IdoA
GlcA
GlcA-1P
UDP-GlcA
UDP-L-Ara4O
UDP-L-Ara4N
UDP-L-Ara4FN
Undecaprenyl phosphate-α-L-Ara4FN
Glc (extracellular)
Glc
Glc-6P
Glc-1P
UDP-Glc
Ascorbate and aldarate metabolism
UDP-4-keto-6-deoxy-D-Glc
UDP-4-keto-Rha
UDP-Rha
Polyketide sugar unit biosynthesis
Galactose metabolism
α-Gal
Gal-1P
UDP-Gal
UDP-SQ
N-Glycan biosynthesis
GalA
GalA-1P
UDP-GalA
Pectin
Starch and sucrose metabolism
GDP-L-Gul
GDP-L-Gal
GDP-D-ManA
Man (extracellular)
Man
Man-6P
Man-1P
GDP-Man
GDP-4-oxo-6-deoxy Man
GDP-D-Rha
GDP-6-deoxy-D-Tal
Fuc (extracellular)
Fuc
Fuc-1P
GDP-Fuc
GDP-Glc
CDP-Glc
CDP-4-keto-6-deoxy-D-Glc
CDP-4-keto-3,6-deoxy-D-Glc
CDP-abequose
CDP-4-dehydro-3,6-dideoxy-D-Glc epimer
CDP-ascarylose
CDP-paratose
CDP-Tyv
ADP-Glc
2.7.1.69
5.1.3.8
4.2.1.126
3.2.1.14
2.7.1.59
5.1.3.9
5.4.2.3
2.7.7.23
4.2.1.115
2.6.1.92
PseH
3.6.1.57
2.5.1.97
2.7.7.81
5.1.3.14
2.7.1.60
2.5.1.56
2.5.1.57
3.1.3.29
2.7.7.43
2.4.1.16
3.2.1.-
4.1.3.3
1.14.18.2
1.6.2.2
3.5.1.41
3.2.1.52
3.5.1.33
3.5.1.25
2.3.1.4
2.3.1.157
2.3.1.3
3.2.1.132
3.2.1.165
2.7.1.1
2.7.1.8
5.4.2.10
3.5.1.-
3.5.99.6
2.6.1.16
1.1.1.-
1.1.1.136
5.1.3.-
5.1.3.7
2.5.1.7
1.1.1.158
2.7.1.4
2.7.1.46
2.7.7.64
3.2.1.55
5.1.3.5
2.7.7.11
2.4.2.24
3.2.1.37
4.1.1.35
AXS
5.1.3.12
5.3.1.9
2.7.1.43
2.7.7.44
1.1.1.305
2.6.1.87
2.1.2.13
2.7.8.30
1.1.1.22
4.1.1.67
2.7.7.9
5.4.2.2
5.4.2.5
2.7.1.2
2.7.1.63
4.2.1.76
5.1.3.6
5.3.1.8
2.7.7.12
5.1.3.2
3.13.1.1
2.7.1.6
2.7.7.10
2.7.1.44
2.7.7.-
2.4.1.43
5.1.3.18
1.1.1.132
2.7.1.7
5.4.2.8
2.7.7.13
2.7.7.22
4.2.1.47
1.1.1.187
1.1.1.281
1.1.1.135
2.7.1.52
2.7.7.30
1.1.1.271
2.7.7.34
RfbF
RfbG
1.17.1.1
RfbJ
AscE
AscF
RfbS
RfbE
2.7.7.27
00520 4/13/12
(c) Kanehisa Laboratories

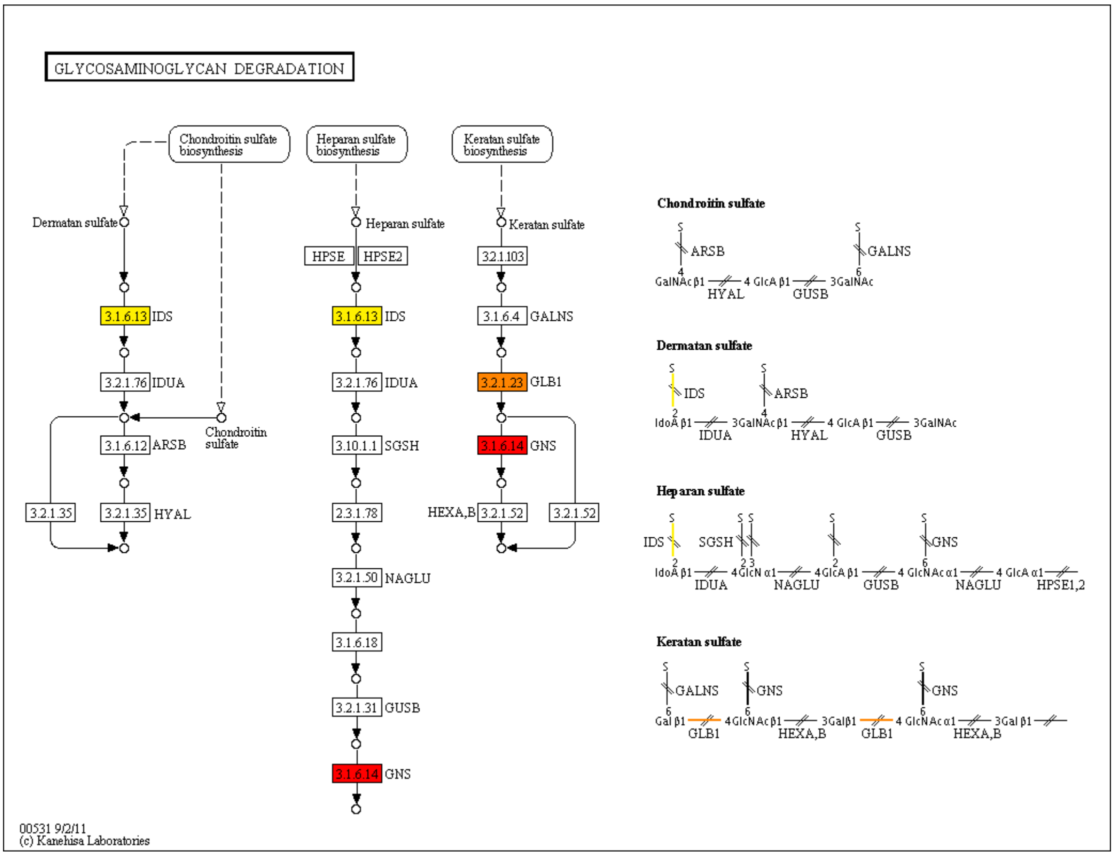
GLYCOSAMINOGLYCAN DEGRADATION
Chondroitin sulfate biosynthesis
Heparan sulfate biosynthesis
Keratan sulfate biosynthesis
Dermatan sulfate
Heparan sulfate
Keratan sulfate
HPSE
HPSE2
3.2.1.103
3.1.6.13 IDS
3.1.6.13 IDS
3.1.6.4 GALNS
3.2.1.76 IDUA
3.2.1.76 IDUA
3.2.1.23 GLB1
Chondroitin sulfate
3.1.6.12 ARSB
3.10.1.1 SGSH
3.1.6.14 GNS
3.2.1.35
3.2.1.35 HYAL
2.3.1.78
HEXA,B 3.2.1.52
3.2.1.52
3.2.1.50 NAGLU
3.1.6.18
3.2.1.31 GUSB
3.1.6.14 GNS
Chondroitin sulfate
ARSB
GALNS
GalNAc β1 4 GlcA β1 3GalNAc
HYAL
GUSB
Dermatan sulfate
IDS
ARSB
IdoA β1 3GalNAc β1 4 GlcA β1 3GalNAc
IDUA
HYAL
GUSB
Heparan sulfate
IDS
SGSH
GNS
IdoA β1 4GlcN α1 4GlcA β1 4 GlcNAc α1 4 GlcA α1
IDUA
NAGLU
GUSB
NAGLU
HPSE1,2
Keratan sulfate
GALNS
GNS
GNS
Gal β1 4 GlcNAc β1 3Gal β1 4 GlcNAc α1 3Gal β1
GLB1
HEXA,B
GLB1
HEXA,B
00531 9/2/11
(c) Kanehisa Laboratories

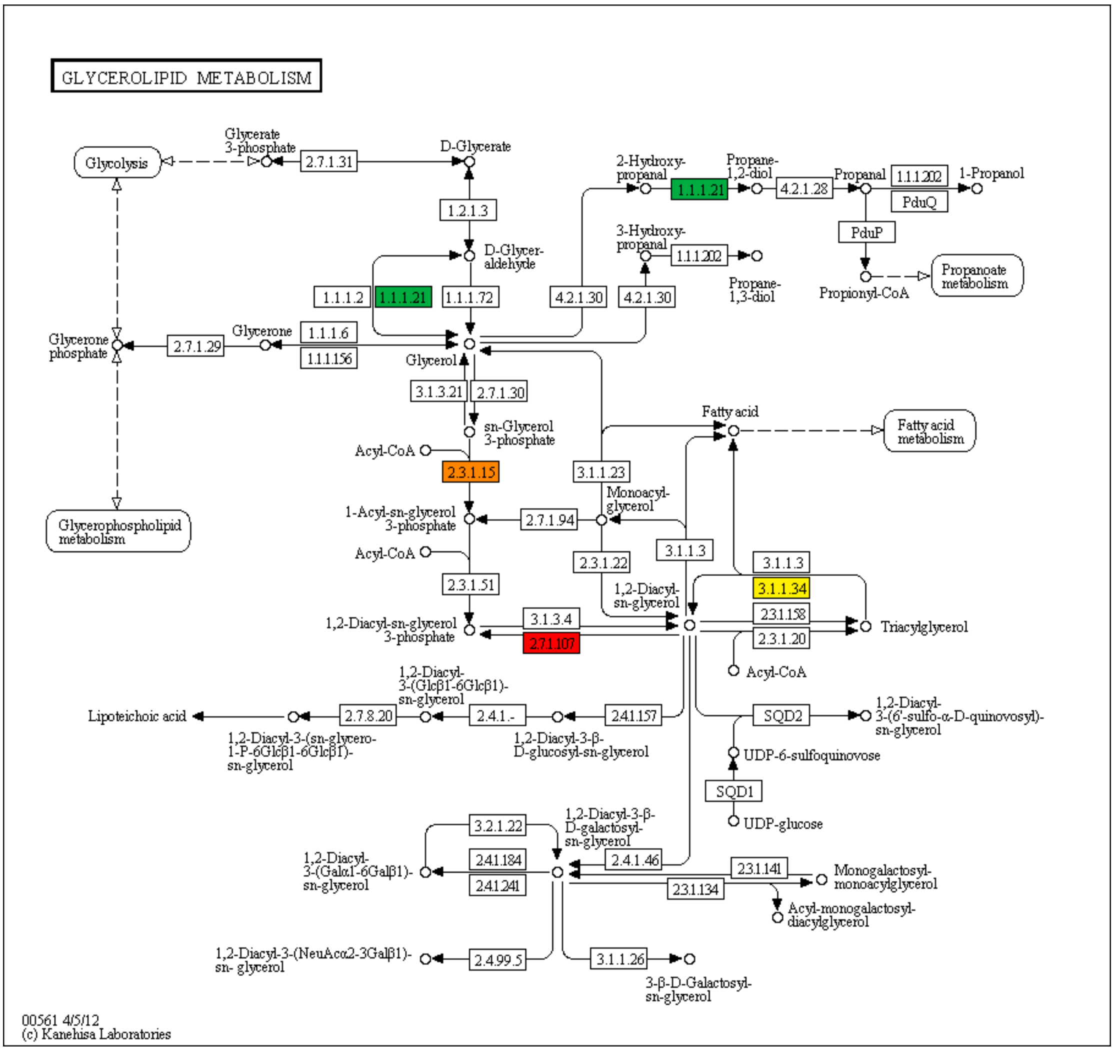
GLYCEROLIPID METABOLISM
Glycolysis
Glycerate 3-phosphate
2.7.1.31
D-Glycerate
1.2.1.3
D-Glyceraldehyde
1.1.1.2
1.1.1.21
1.1.1.72
2-Hydroxy-propanal
1.1.1.21
Propane-1,2-diol
4.2.1.28
Propanal
1.1.1.202
1-Propanol
PduQ
PduP
Propionyl-CoA
Propanoate metabolism
3-Hydroxy-propanal
1.1.1.202
Propane-1,3-diol
4.2.1.30
4.2.1.30
Glycerone phosphate
2.7.1.29
Glycerone
1.1.1.6
1.1.1.156
Glycerol
3.1.3.21
2.7.1.30
sn-Glycerol 3-phosphate
Acyl-CoA
2.3.1.15
3.1.1.23
Fatty acid
Fatty acid metabolism
Monoacylglycerol
1-Acyl-sn-glycerol 3-phosphate
2.7.1.94
Glycerophospholipid metabolism
Acyl-CoA
2.3.1.51
2.3.1.22
3.1.1.3
3.1.1.3
3.1.1.34
1,2-Diacyl-sn-glycerol
2.3.1.158
2.3.1.20
Triacylglycerol
1,2-Diacyl-sn-glycerol 3-phosphate
3.1.3.4
2.7.1.107
Acyl-CoA
1,2-Diacyl-3-(Glcβ1-6Glcβ1)-sn-glycerol
Lipoteichoic acid
2.7.8.20
2.4.1.-
2.4.1.157
1,2-Diacyl-3-(sn-glycero-1-P-6Glcβ1-6Glcβ1)-sn-glycerol
1,2-Diacyl-3-β-D-glucosyl-sn-glycerol
SQD2
1,2-Diacyl-3-(6'-sulfo-α-D-quinovosyl)-sn-glycerol
UDP-6-sulfoquinovose
SQD1
UDP-glucose
3.2.1.22
1,2-Diacyl-3-β-D-galactosyl-sn-glycerol
2.4.1.184
2.4.1.241
2.4.1.46
2.3.1.141
2.3.1.134
1,2-Diacyl-3-(Galα1-6Galβ1)-sn-glycerol
Monogalactosyl-monoacylglycerol
Acyl-monogalactosyl-diacylglycerol
1,2-Diacyl-3-(NeuAcα2-3Galβ1)-sn-glycerol
2.4.99.5
3.1.1.26
3-β-D-Galactosyl-sn-glycerol
00561 4/5/12
(c) Kanehisa Laboratories

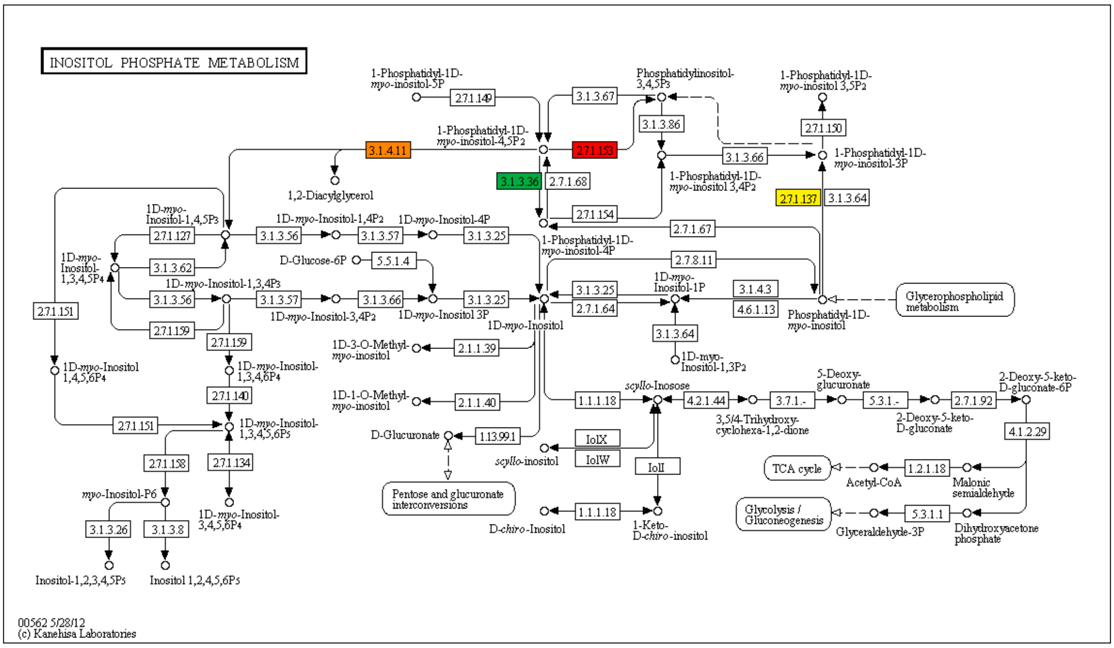
INOSITOL PHOSPHATE METABOLISM
1-Phosphatidyl-1D-myo-inositol-5P
2.7.1.149
3.1.3.67
Phosphatidylinositol-3,4,5P3
1-Phosphatidyl-1D-myo-inositol 3,5P2
3.1.3.86
2.7.1.150
1-Phosphatidyl-1D-myo-inositol-4,5P2
3.1.4.11
2.7.1.153
3.1.3.66
1-Phosphatidyl-1D-myo-inositol-3P
1,2-Diacylglycerol
3.1.3.36
2.7.1.68
1-Phosphatidyl-1D-myo-inositol 3,4P2
2.7.1.137
3.1.3.64
2.7.1.154
2.7.1.67
1D-myo-Inositol-1,4,5P3
1D-myo-Inositol-1,4P2
1D-myo-Inositol-4P
2.7.1.127
3.1.3.56
3.1.3.57
3.1.3.25
1-Phosphatidyl-1D-myo-inositol-4P
1D-myo-Inositol-1,3,4,5P4
3.1.3.62
D-Glucose-6P
5.5.1.4
2.7.8.11
1D-myo-Inositol-1,3,4P3
1D-myo-Inositol 1P
3.1.3.56
3.1.3.57
3.1.3.66
3.1.3.25
3.1.3.25
3.1.4.3
2.7.1.151
2.7.1.159
1D-myo-Inositol-3,4P2
1D-myo-Inositol 3P
2.7.1.64
4.6.1.13
Glycerophospholipid metabolism
1D-myo-Inositol
Phosphatidyl-1D-myo-inositol
3.1.3.64
1D-myo-Inositol-1,3P2
2.7.1.159
1D-3-O-Methyl-myo-inositol
2.1.1.39
1D-myo-Inositol 1,4,5,6P4
1D-myo-Inositol-1,3,4,6P4
1D-1-O-Methyl-myo-inositol
2.1.1.40
1.1.1.18
scyllo-Inosose
4.2.1.44
3.7.1.-
5-Deoxy-glucuronate
5.3.1.-
2.7.1.92
2-Deoxy-5-keto-D-gluconate-6P
2.7.1.140
3,5/4-Trihydroxy-cyclohexa-1,2-dione
2-Deoxy-5-keto-D-gluconate
4.1.2.29
2.7.1.151
1D-myo-Inositol-1,3,4,5,6P5
D-Glucuronate
1.13.99.1
IolX
IolW
scyllo-inositol
IolI
TCA cycle
Acetyl-CoA
1.2.1.18
Malonic semialdehyde
2.7.1.158
2.7.1.134
myo-Inositol-P6
Pentose and glucuronate interconversions
1D-myo-Inositol-3,4,5,6P4
1.1.1.18
D-chiro-Inositol
1-Keto-D-chiro-inositol
Glycolysis / Gluconeogenesis
5.3.1.1
Glyceraldehyde-3P
Dihydroxyacetone phosphate
3.1.3.26
3.1.3.8
Inositol-1,2,3,4,5P5
Inositol 1,2,4,5,6P5
00562 5/28/12
(c) Kanehisa Laboratories

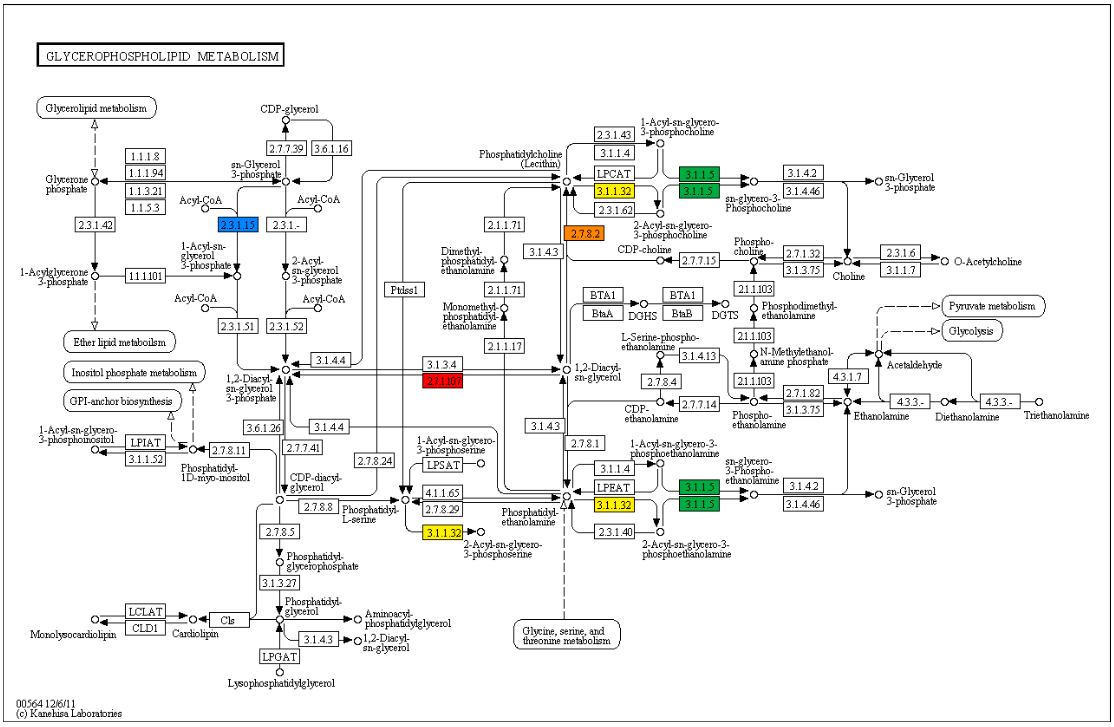
GLYCEROPHOSPHOLIPID METABOLISM
Glycerolipid metabolism
CDP-glycerol
2.7.7.39
3.6.1.16
sn-Glycerol
3-phosphate
1.1.1.8
1.1.1.94
1.1.3.21
1.1.5.3
Glycerone
phosphate
Acyl-CoA
2.3.1.15
2.3.1.-
2.3.1.42
1-Acyl-sn-
glycerol
3-phosphate
2-Acyl-
sn-glycerol
3-phosphate
1-Acylglycerone
3-phosphate
1.1.1.101
2.3.1.51
2.3.1.52
Ether lipid metabolism
Inositol phosphate metabolism
GPI-anchor biosynthesis
1,2-Diacyl-
sn-glycerol
3-phosphate
3.1.4.4
3.6.1.26
2.7.7.41
2.7.8.11
1-Acyl-sn-glycero-
3-phosphoinositol
LPIAT
3.1.1.52
Phosphatidyl-
1D-myo-inositol
CDP-diacyl-
glycerol
2.7.8.24
2.7.8.8
Phosphatidyl-
L-serine
2.7.8.5
Phosphatidyl-
glycerophosphate
3.1.3.27
Phosphatidyl-
glycerol
Aminoacyl-
phosphatidylglycerol
3.1.4.3
1,2-Diacyl-
sn-glycerol
LPGAT
Lysophosphatidylglycerol
LCLAT
CLD1
Cls
Monolysocardiolipin
Cardiolipin
Phosphatidylcholine
(Lecithin)
2.3.1.43
3.1.1.4
LPCAT
3.1.1.32
2.3.1.62
1-Acyl-sn-glycero-
3-phosphocholine
3.1.1.5
3.1.4.2
3.1.4.46
sn-Glycerol
3-phosphate
sn-glycero-3-
Phosphocholine
2-Acyl-sn-glycero-
3-phosphocholine
2.7.8.2
2.1.1.71
3.1.4.3
Dimethyl-
phosphatidyl-
ethanolamine
Ptdss1
Monomethyl-
phosphatidyl-
ethanolamine
2.1.1.17
3.1.3.4
2.7.1.107
1,2-Diacyl-
sn-glycerol
CDP-choline
2.7.7.15
Phospho-
choline
2.7.1.32
3.1.3.75
Choline
2.3.1.6
3.1.1.7
O-Acetylcholine
BTA1
BtaA
DGHS
BtaB
DGTS
2.1.1.103
Phosphodimethyl-
ethanolamine
Pyruvate metabolism
Glycolysis
L-Serine-phospho-
ethanolamine
3.1.4.13
N-Methylethanol-
amine phosphate
Acetaldehyde
2.7.8.4
4.3.1.7
2.7.7.14
2.7.1.82
3.1.3.75
CDP-
ethanolamine
Phospho-
ethanolamine
Ethanolamine
4.3.3.-
Diethanolamine
Triethanolamine
3.1.4.3
2.7.8.1
1-Acyl-sn-glycero-
3-phosphoserine
LPSAT
4.1.1.65
2.7.8.29
3.1.1.32
2-Acyl-sn-glycero-
3-phosphoserine
Phosphatidyl-
ethanolamine
LPEAT
2.3.1.40
1-Acyl-sn-glycero-3-
phosphoethanolamine
sn-glycero-
3-Phospho-
ethanolamine
2-Acyl-sn-glycero-3-
phosphoethanolamine
Glycine, serine, and
threonine metabolism
00564 12/6/11
(c) Kanehisa Laboratories

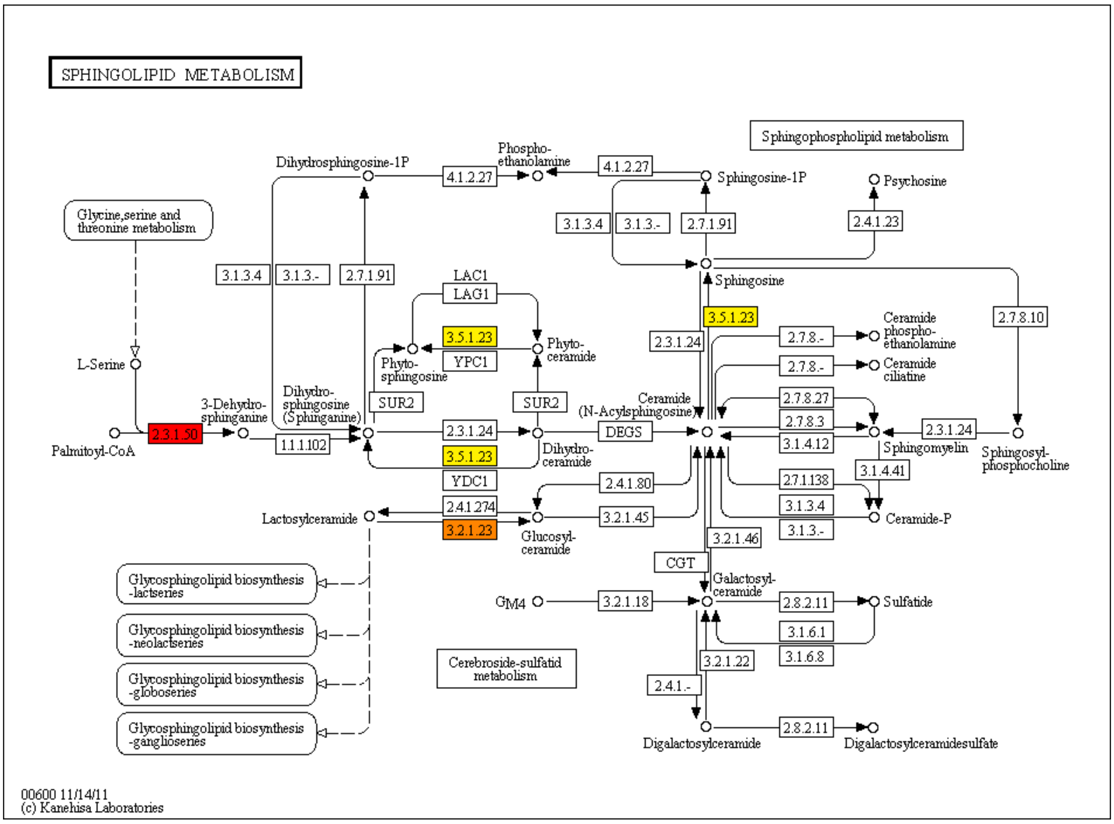
SPHINGOLIPID METABOLISM
Sphingophospholipid metabolism
Dihydrosphingosine-1P
4.1.2.27
Phospho-
ethanolamine
Sphingosine-1P
Psychosine
Glycine,serine and
threonine metabolism
3.1.3.4
3.1.3.-
2.7.1.91
2.4.1.23
Sphingosine
LAC1
LAG1
3.5.1.23
YPC1
Phyto-
sphingosine
Phyto-
ceramide
SUR2
L-Serine
3-Dehydro-
sphingosine
Dihydro-
sphingosine
(Sphinganine)
2.3.1.24
2.7.8.-
Ceramide
phospho-
ethanolamine
Ceramide
ciliatine
2.7.8.10
Ceramide
(N-Acylsphingosine)
2.7.8.27
2.7.8.3
Palmitoyl-CoA
2.3.1.50
1.1.1.102
DEGS
Dihydro-
ceramide
YDC1
3.1.4.12
Sphingomyelin
Sphingosyl-
phosphocholine
3.1.4.41
2.7.1.138
Ceramide-P
2.4.1.80
Lactosylceramide
2.4.1.274
3.2.1.23
3.2.1.45
Glucosyl-
ceramide
3.2.1.46
CGT
Galactosyl-
ceramide
Glycosphingolipid biosynthesis
-lactseries
-neolactseries
-globoseries
-ganglioseries
GM4
3.2.1.18
2.8.2.11
Sulfatide
3.1.6.1
3.1.6.8
3.2.1.22
Cerebroside-sulfatid
metabolism
2.4.1.-
Digalactosylceramide
Digalactosylceramidesulfate
00600 11/14/11
(c) Kanehisa Laboratories

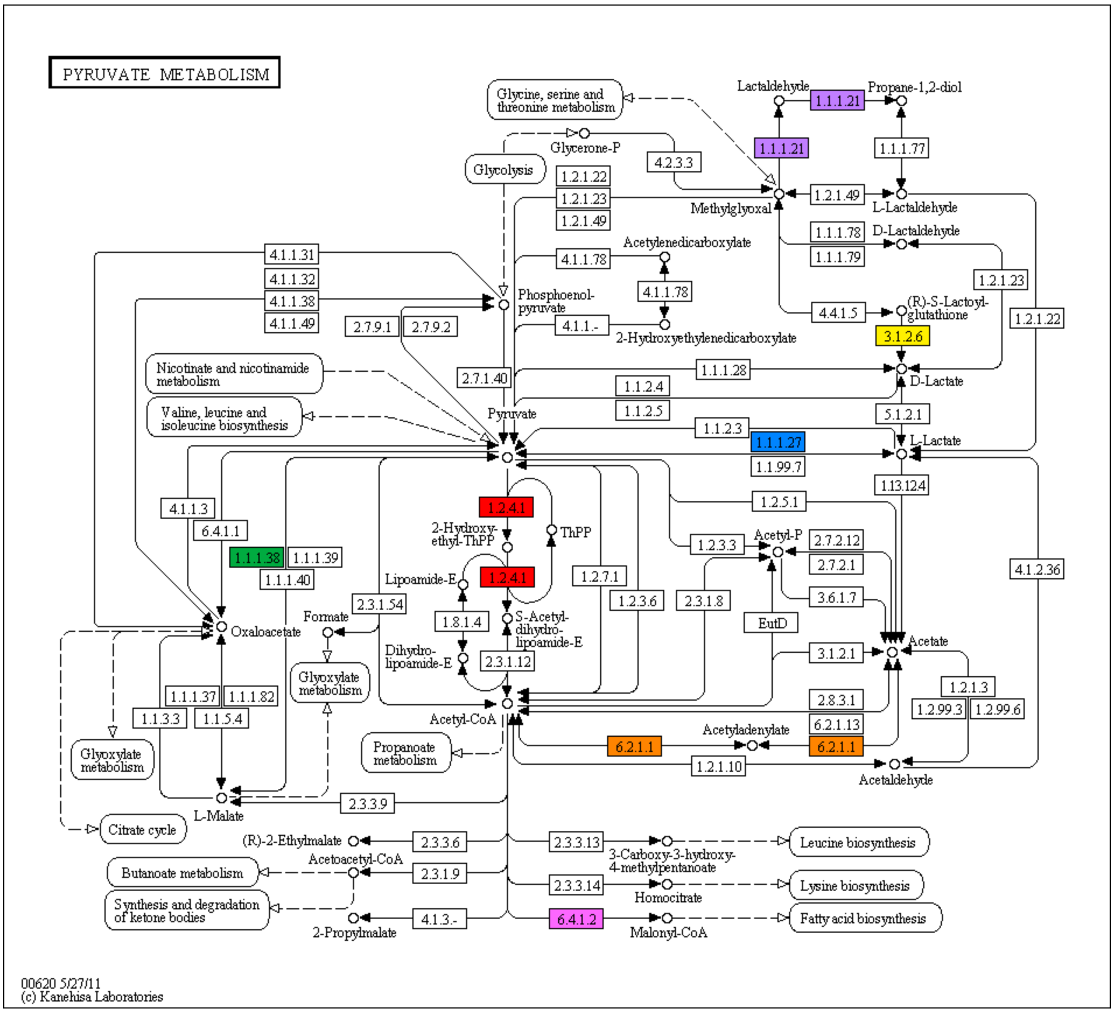
PYRUVATE METABOLISM
Glycine, serine and threonine metabolism
Lactaldehyde
1.1.1.21
Propane-1,2-diol
Glycerone-P
Glycolysis
4.2.3.3
1.2.1.22
1.2.1.23
1.2.1.49
Methylglyoxal
1.2.1.49
L-Lactaldehyde
1.1.1.77
1.1.1.78
D-Lactaldehyde
1.1.1.79
Acetylenedicarboxylate
4.1.1.78
4.1.1.31
4.1.1.32
4.1.1.38
4.1.1.49
Phosphoenol-pyruvate
2.7.9.1
2.7.9.2
4.1.1.-
2-Hydroxyethylenedicarboxylate
4.4.1.5
(R)-S-Lactoyl-glutathione
3.1.2.6
1.2.1.23
1.2.1.22
Nicotinate and nicotinamide metabolism
2.7.1.40
1.1.1.28
D-Lactate
1.1.2.4
1.1.2.5
Valine, leucine and isoleucine biosynthesis
Pyruvate
1.1.2.3
1.1.1.27
1.1.99.7
5.1.2.1
L-Lactate
1.13.12.4
4.1.1.3
6.4.1.1
1.2.4.1
1.2.5.1
2-Hydroxy-ethyl-ThPP
ThPP
1.1.1.38
1.1.1.39
1.1.1.40
Lipoamide-E
1.2.4.1
1.2.7.1
1.2.3.3
Acetyl-P
2.7.2.12
2.7.2.1
3.6.1.7
4.1.2.36
2.3.1.54
Formate
1.8.1.4
S-Acetyl-dihydro-lipoamide-E
1.2.3.6
2.3.1.8
EutD
Oxaloacetate
Dihydro-lipoamide-E
2.3.1.12
3.1.2.1
Acetate
Glyoxylate metabolism
1.2.1.3
1.1.1.37
1.1.1.82
2.8.3.1
1.2.99.3
1.2.99.6
1.1.3.3
1.1.5.4
Acetyl-CoA
6.2.1.13
Glyoxylate metabolism
Propanoate metabolism
Acetyladenylate
6.2.1.1
6.2.1.1
1.2.1.10
Acetaldehyde
2.3.3.9
L-Malate
Citrate cycle
(R)-2-Ethylmalate
2.3.3.6
2.3.3.13
Leucine biosynthesis
Acetoacetyl-CoA
3-Carboxy-3-hydroxy-4-methylpentanoate
Butanoate metabolism
2.3.1.9
2.3.3.14
Lysine biosynthesis
Homocitrate
Synthesis and degradation of ketone bodies
4.1.3.-
6.4.1.2
Fatty acid biosynthesis
2-Propylmalate
Malonyl-CoA
00620 5/27/11
(c) Kanehisa Laboratories

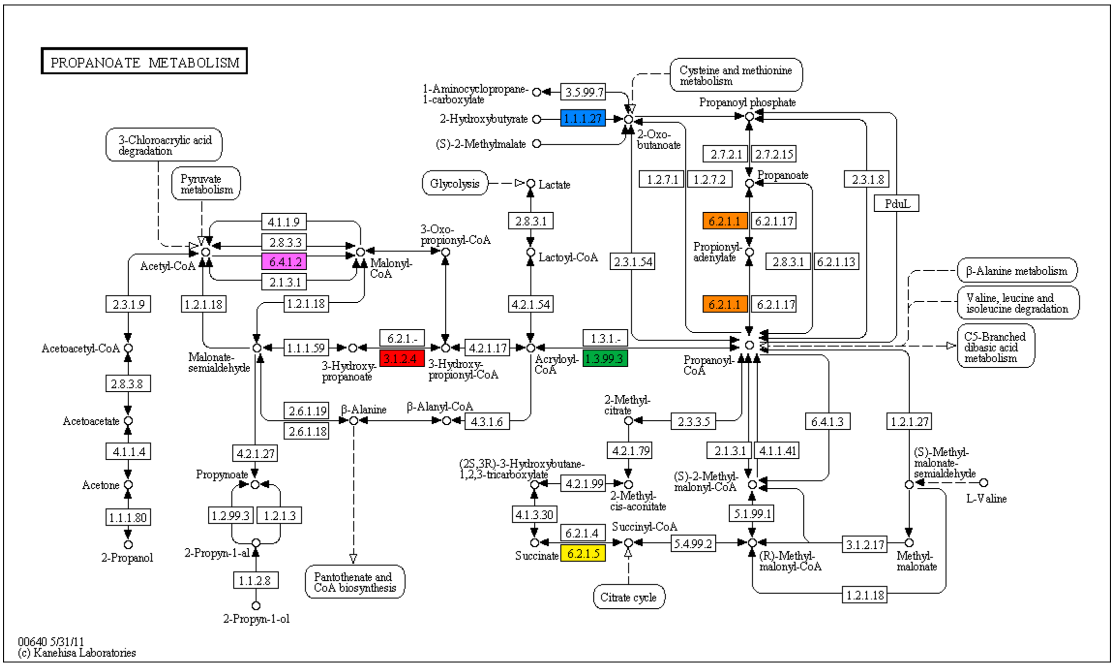
PROPANOATE METABOLISM
Cysteine and methionine metabolism
1-Aminocyclopropane-1-carboxylate
3.5.99.7
Propanoyl phosphate
2-Hydroxybutyrate
1.1.1.27
2-Oxo-butanoate
(S)-2-Methylmalate
3-Chloroacrylic acid degradation
2.7.2.1
2.7.2.15
Pyruvate metabolism
Glycolysis
Lactate
1.2.7.1
1.2.7.2
Propanoate
2.3.1.8
PduL
4.1.1.9
2.8.3.1
2.8.3.3
3-Oxo-propionyl-CoA
6.2.1.1
6.2.1.17
Acetyl-CoA
6.4.1.2
Malonyl-CoA
Lactoyl-CoA
Propionyl-adenylate
2.8.3.1
6.2.1.13
β-Alanine metabolism
2.1.3.1
2.3.1.9
1.2.1.18
1.2.1.18
4.2.1.54
2.3.1.54
Valine, leucine and isoleucine degradation
6.2.1.1
6.2.1.17
Acetoacetyl-CoA
Malonate-semialdehyde
1.1.1.59
6.2.1.-
3.1.2.4
4.2.1.17
1.3.1.-
1.3.99.3
C5-Branched dibasic acid metabolism
3-Hydroxy-propanoate
3-Hydroxy-propionyl-CoA
Acryloyl-CoA
Propanoyl-CoA
2.8.3.8
2.6.1.19
β-Alanine
β-Alanyl-CoA
2-Methyl-citrate
Acetoacetate
2.6.1.18
4.3.1.6
2.3.3.5
6.4.1.3
1.2.1.27
4.1.1.4
4.2.1.27
4.2.1.79
2.1.3.1
4.1.1.41
(S)-Methyl-malonate-semialdehyde
Acetone
Propynoate
(2S,3R)-3-Hydroxybutane-1,2,3-tricarboxylate
4.2.1.99
(S)-2-Methyl-malonyl-CoA
L-Valine
2-Methyl-cis-aconitate
1.1.1.80
1.2.99.3
1.2.1.3
4.1.3.30
5.1.99.1
Succinyl-CoA
6.2.1.4
2-Propanol
2-Propyn-1-al
Succinate
6.2.1.5
5.4.99.2
3.1.2.17
(R)-Methyl-malonyl-CoA
Methyl-malonate
Pantothenate and CoA biosynthesis
1.1.2.8
Citrate cycle
1.2.1.18
2-Propyn-1-ol
00640 5/31/11
(c) Kanehisa Laboratories

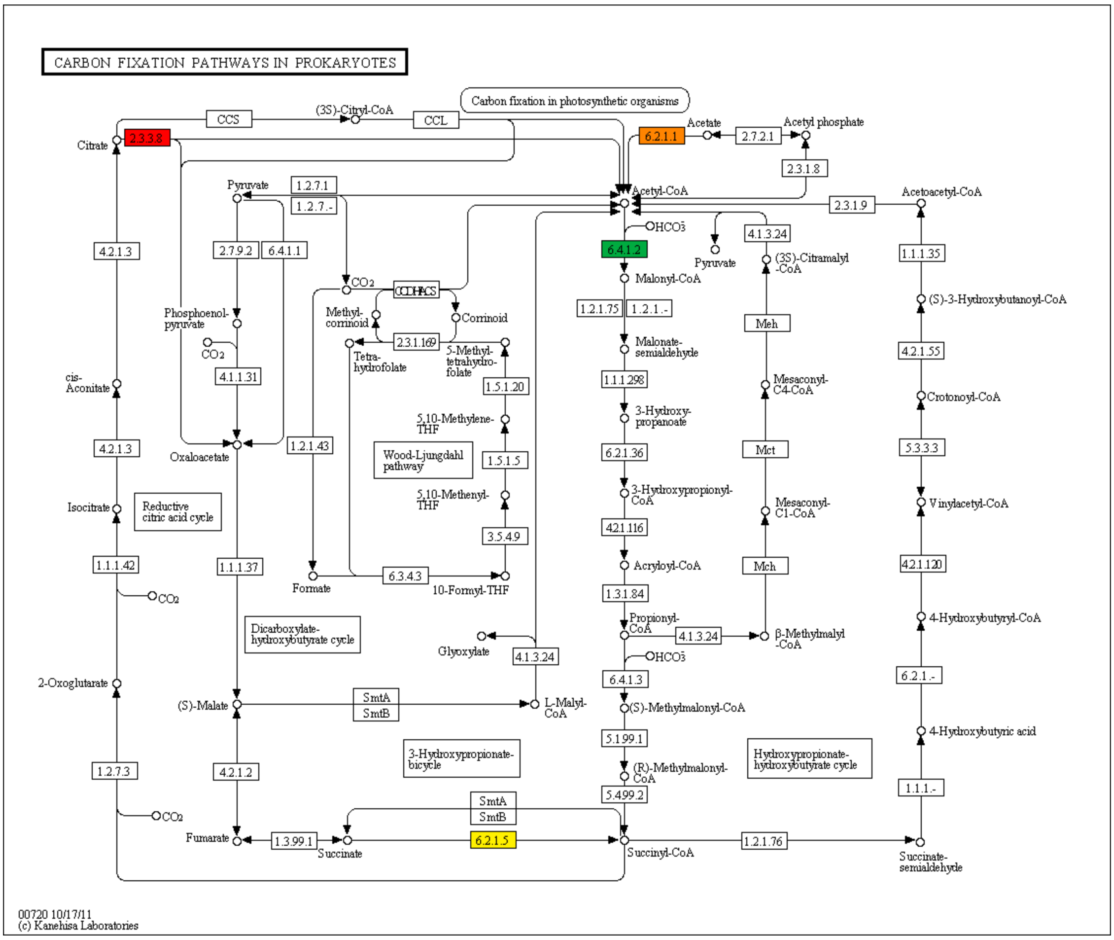
CARBON FIXATION PATHWAYS IN PROKARYOTES
Carbon fixation in photosynthetic organisms
Citrate
2.3.3.8
CCS
(3S)-Citryl-CoA
CCL
6.2.1.1
Acetate
2.7.2.1
Acetyl phosphate
2.3.1.8
Acetyl-CoA
2.3.1.9
Acetoacetyl-CoA
Pyruvate
1.2.7.1
1.2.7.-
4.2.1.3
2.7.9.2
6.4.1.1
HCO3
6.4.1.2
Malonyl-CoA
Pyruvate
4.1.3.24
(3S)-Citramalyl-CoA
1.1.1.35
(S)-3-Hydroxybutanoyl-CoA
Phosphoenol-pyruvate
CO2
CODH/ACS
Methyl-corrinoid
Corrinoid
1.2.1.75
1.2.1.-
Mch
2.3.1.169
Tetra-hydrofolate
5-Methyl-tetrahydro-folate
Malonate-semialdehyde
4.2.1.55
cis-Aconitate
4.1.1.31
1.5.1.20
1.1.1.298
Mesaconyl-C4-CoA
Crotonoyl-CoA
5,10-Methylene-THF
3-Hydroxy-propanoate
4.2.1.3
Oxaloacetate
1.2.1.43
Wood-Ljungdahl pathway
1.5.1.5
6.2.1.36
Mct
5.3.3.3
Isocitrate
Reductive citric acid cycle
5,10-Methenyl-THF
3-Hydroxypropionyl-CoA
Mesaconyl-C1-CoA
Vinylacetyl-CoA
3.5.4.9
4.2.1.116
1.1.1.42
1.1.1.37
Formate
6.3.4.3
10-Formyl-THF
Acryloyl-CoA
Mch
4.2.1.120
CO2
1.3.1.84
4-Hydroxybutyryl-CoA
Dicarboxylate-hydroxybutyrate cycle
Propionyl-CoA
4.1.3.24
β-Methylmalyl-CoA
Glyoxylate
4.1.3.24
HCO3
6.4.1.3
6.2.1.-
2-Oxoglutarate
SmtA
SmtB
(S)-Malate
L-Malyl-CoA
(S)-Methylmalonyl-CoA
4-Hydroxybutyric acid
3-Hydroxypropionate-bicycle
5.1.99.1
Hydroxypropionate-hydroxybutyrate cycle
(R)-Methylmalonyl-CoA
1.2.7.3
4.2.1.2
1.1.1.-
5.4.99.2
SmtA
SmtB
CO2
Fumarate
1.3.99.1
6.2.1.5
Succinate
Succinyl-CoA
1.2.1.76
Succinate-semialdehyde
00720 10/17/11
(c) Kanehisa Laboratories

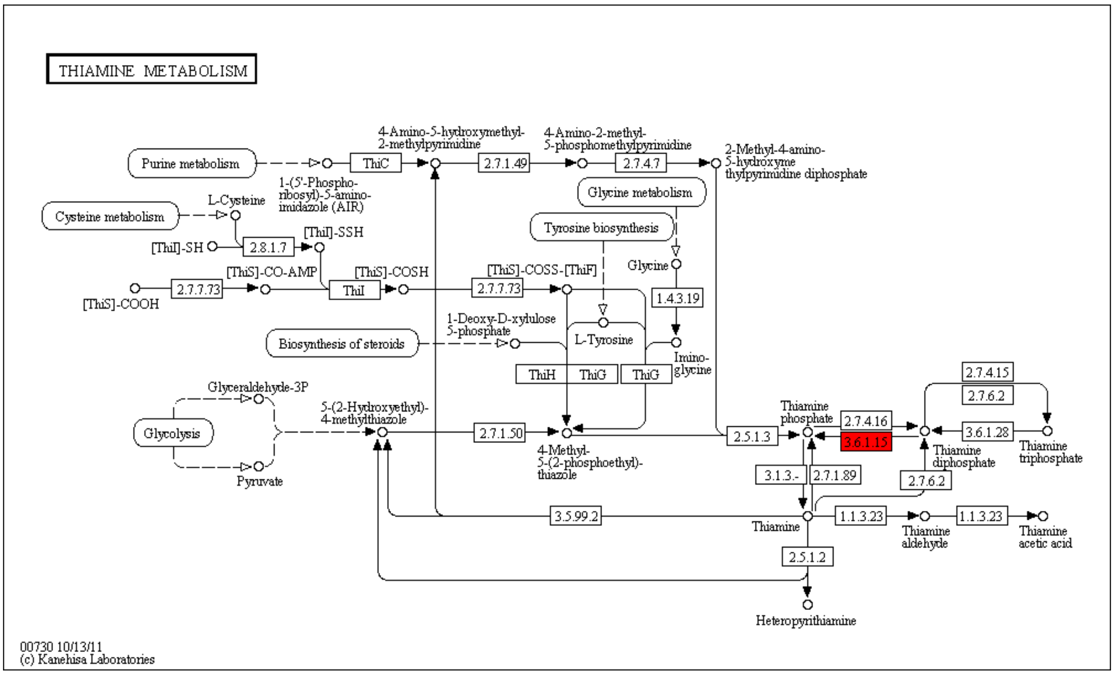
THIAMINE METABOLISM
4-Amino-5-hydroxymethyl-2-methylpyrimidine
4-Amino-2-methyl-5-phosphomethylpyrimidine
Purine metabolism
ThiC
2.7.1.49
2.7.4.7
2-Methyl-4-amino-5-hydroxymethylpyrimidine diphosphate
1-(5'-Phospho-ribosyl)-5-amino-imidazole (AIR)
Glycine metabolism
L-Cysteine
Cysteine metabolism
Tyrosine biosynthesis
[ThiI]-SH
2.8.1.7
[ThiI]-SSH
Glycine
[ThiS]-CO-AMP
[ThiS]-COSH
[ThiS]-COSS-[ThiF]
2.7.7.73
ThiI
2.7.7.73
1.4.3.19
[ThiS]-COOH
1-Deoxy-D-xylulose 5-phosphate
Biosynthesis of steroids
L-Tyrosine
Imino-glycine
ThiH
ThiG
ThiG
2.7.4.15
Glyceraldehyde-3P
2.7.6.2
5-(2-Hydroxyethyl)-4-methylthiazole
Thiamine phosphate
2.7.4.16
Glycolysis
2.7.1.50
2.5.1.3
3.6.1.15
3.6.1.28
Thiamine diphosphate
Thiamine triphosphate
4-Methyl-5-(2-phosphoethyl)-thiazole
Pyruvate
3.1.3.-
2.7.1.89
2.7.6.2
3.5.99.2
Thiamine
1.1.3.23
1.1.3.23
Thiamine aldehyde
Thiamine acetic acid
2.5.1.2
Heteropyrithiamine
00730 10/13/11
(c) Kanehisa Laboratories

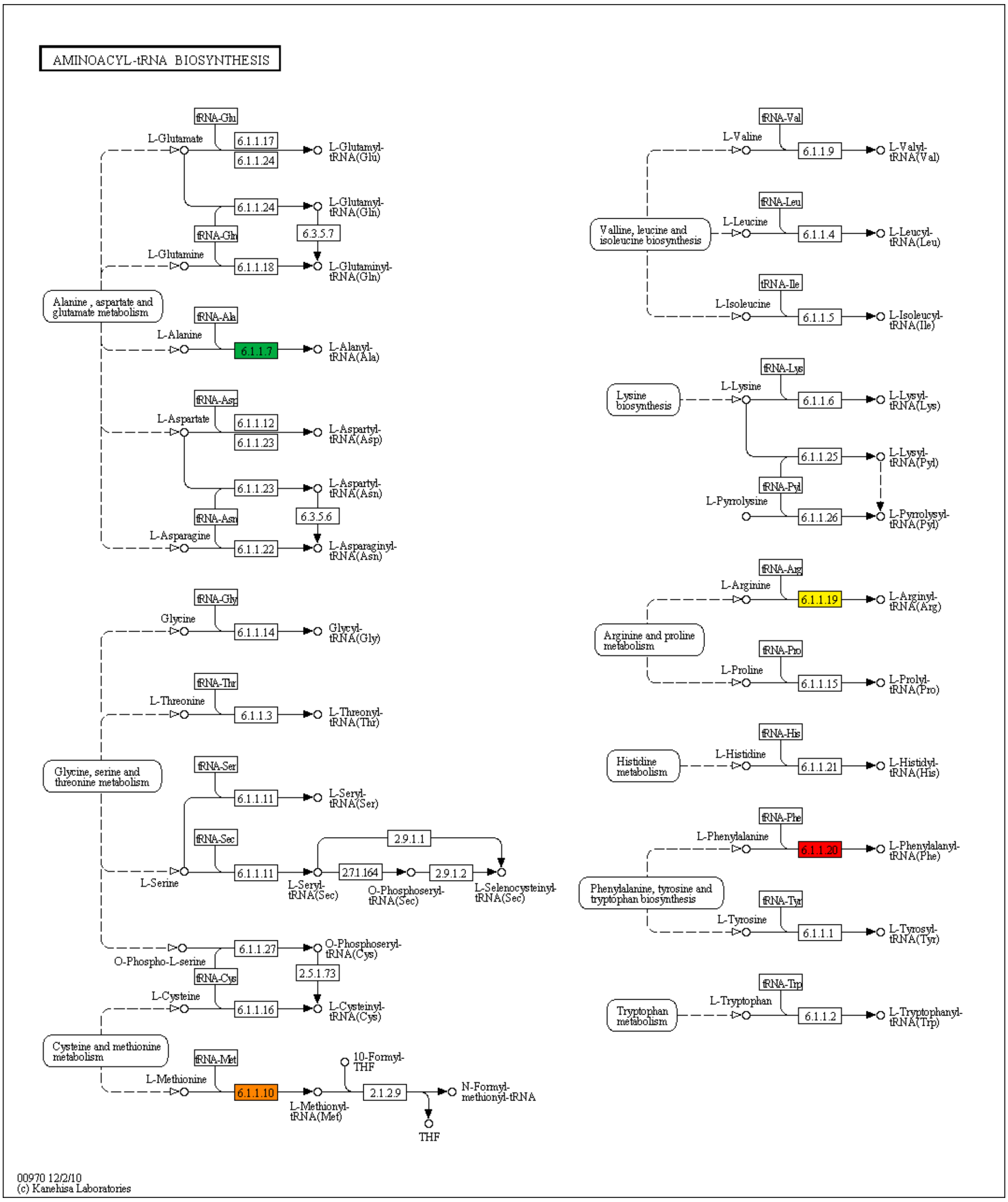
AMINOACYL-tRNA BIOSYNTHESIS
tRNA-Glu
L-Glutamate
6.1.1.17
6.1.1.24
L-Glutamyl-tRNA(Glu)
L-Glutamyl-tRNA(Gln)
6.3.5.7
tRNA-Gln
L-Glutamine
6.1.1.18
L-Glutaminyl-tRNA(Gln)
Alanine , aspartate and glutamate metabolism
tRNA-Ala
L-Alanine
6.1.1.7
L-Alanyl-tRNA(Ala)
tRNA-Asp
L-Aspartate
6.1.1.12
6.1.1.23
L-Aspartyl-tRNA(Asp)
L-Aspartyl-tRNA(Asn)
6.3.5.6
tRNA-Asn
L-Asparagine
6.1.1.22
L-Asparaginyl-tRNA(Asn)
tRNA-Gly
Glycine
6.1.1.14
Glycyl-tRNA(Gly)
tRNA-Thr
L-Threonine
6.1.1.3
L-Threonyl-tRNA(Thr)
Glycine, serine and threonine metabolism
tRNA-Ser
6.1.1.11
L-Seryl-tRNA(Ser)
tRNA-Sec
2.9.1.1
2.7.1.164
2.9.1.2
L-Serine
L-Seryl-tRNA(Sec)
O-Phosphoseryl-tRNA(Sec)
L-Selenocysteinyl-tRNA(Sec)
6.1.1.27
O-Phosphoseryl-tRNA(Cys)
O-Phospho-L-serine
tRNA-Cys
2.5.1.73
L-Cysteine
6.1.1.16
L-Cysteinyl-tRNA(Cys)
Cysteine and methionine metabolism
tRNA-Met
L-Methionine
6.1.1.10
10-Formyl-THF
2.1.2.9
L-Methionyl-tRNA(Met)
N-Formyl-methionyl-tRNA
THF
tRNA-Val
L-Valine
6.1.1.9
L-Valyl-tRNA(Val)
tRNA-Leu
Valline, leucine and isoleucine biosynthesis
L-Leucine
6.1.1.4
L-Leucyl-tRNA(Leu)
tRNA-Ile
L-Isoleucine
6.1.1.5
L-Isoleucyl-tRNA(Ile)
tRNA-Lys
Lysine biosynthesis
L-Lysine
6.1.1.6
L-Lysyl-tRNA(Lys)
6.1.1.25
L-Lysyl-tRNA(Pyl)
tRNA-Pyl
L-Pyrrolysine
6.1.1.26
L-Pyrrolysyl-tRNA(Pyl)
tRNA-Arg
L-Arginine
6.1.1.19
L-Arginyl-tRNA(Arg)
Arginine and proline metabolism
tRNA-Pro
L-Proline
6.1.1.15
L-Prolyl-tRNA(Pro)
tRNA-His
Histidine metabolism
L-Histidine
6.1.1.21
L-Histidyl-tRNA(His)
tRNA-Phe
L-Phenylalanine
6.1.1.20
L-Phenylalanyl-tRNA(Phe)
Phenylalanine, tyrosine and tryptophan biosynthesis
tRNA-Tyr
L-Tyrosine
6.1.1.1
L-Tyrosyl-tRNA(Tyr)
tRNA-Trp
Tryptophan metabolism
L-Tryptophan
6.1.1.2
L-Tryptophanyl-tRNA(Trp)
00970 12/2/10
(c) Kanehisa Laboratories

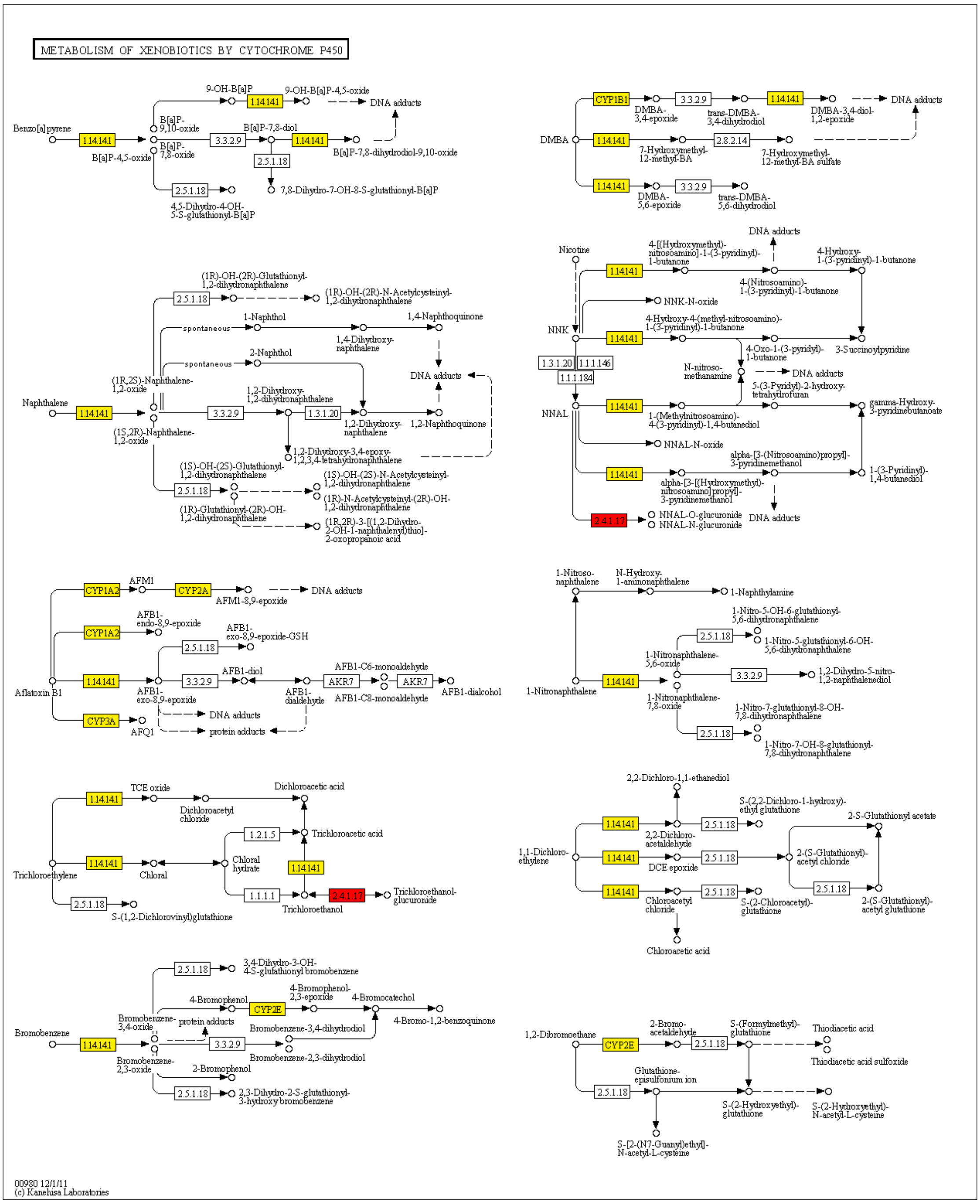
METABOLISM OF XENOBIOTICS BY CYTOCHROME P450
Benzo[a]pyrene
1.14.14.1
B[a]P-4,5-oxide
B[a]P-9,10-oxide
B[a]P-7,8-oxide
9-OH-B[a]P
9-OH-B[a]P-4,5-oxide
DNA adducts
B[a]P-7,8-diol
3.3.2.9
2.5.1.18
B[a]P-7,8-dihydrodiol-9,10-oxide
7,8-Dihydro-7-OH-8-S-glutathionyl-B[a]P
4,5-Dihydro-4-OH-5-S-glutathionyl-B[a]P
DMBA
CYP1B1
DMBA-3,4-epoxide
trans-DMBA-3,4-dihydrodiol
DMBA-3,4-diol-1,2-epoxide
7-Hydroxymethyl-12-methyl-BA
2.8.2.14
7-Hydroxymethyl-12-methyl-BA sulfate
DMBA-5,6-epoxide
trans-DMBA-5,6-dihydrodiol
Naphthalene
(1R,2S)-Naphthalene-1,2-oxide
(1S,2R)-Naphthalene-1,2-oxide
(1R)-OH-(2R)-Glutathionyl-1,2-dihydronaphthalene
(1R)-OH-(2R)-N-Acetylcysteinyl-1,2-dihydronaphthalene
spontaneous
1-Naphthol
1,4-Dihydroxy-naphthalene
1,4-Naphthoquinone
2-Naphthol
1,2-Dihydroxy-1,2-dihydronaphthalene
3.1.3.20
1,2-Dihydroxy-naphthalene
1,2-Naphthoquinone
1,2-Dihydroxy-3,4-epoxy-1,2,3,4-tetrahydronaphthalene
(1S)-OH-(2S)-Glutathionyl-1,2-dihydronaphthalene
(1S)-OH-(2S)-N-Acetylcysteinyl-1,2-dihydronaphthalene
(1R)-N-Acetylcysteinyl-(2R)-OH-1,2-dihydronaphthalene
(1R)-Glutathionyl-(2R)-OH-1,2-dihydronaphthalene
(1R,2R)-3-[(1,2-Dihydro-2-OH-1-naphthalenyl)thio]-2-oxopropanoic acid
Nicotine
NNK
4-[(Hydroxymethyl)-nitrosoamino]-1-(3-pyridinyl)-1-butanone
4-(Nitrosoamino)-1-(3-pyridinyl)-1-butanone
4-Hydroxy-1-(3-pyridinyl)-1-butanone
NNK-N-oxide
4-Hydroxy-4-(methyl-nitrosoamino)-1-(3-pyridinyl)-1-butanone
4-Oxo-1-(3-pyridyl)-1-butanone
3-Succinoylpyridine
1.3.1.20
1.1.1.146
1.1.1.184
N-nitroso-methanamine
5-(3-Pyridyl)-2-hydroxy-tetrahydrofuran
NNAL
1-(Methylnitrosoamino)-4-(3-pyridinyl)-1,4-butanediol
gamma-Hydroxy-3-pyridinebutanoate
NNAL-N-oxide
alpha-[3-(Nitrosoamino)propyl]-3-pyridinemethanol
1-(3-Pyridinyl)-1,4-butanediol
alpha-[3-[(Hydroxymethyl)-nitrosoamino]propyl]-3-pyridinemethanol
2.4.1.17
NNAL-O-glucuronide
NNAL-N-glucuronide
Aflatoxin B1
CYP1A2
AFM1
CYP2A
AFM1-8,9-epoxide
AFB1-endo-8,9-epoxide
AFB1-exo-8,9-epoxide-GSH
AFB1-exo-8,9-epoxide
AFB1-diol
AFB1-dialdehyde
AKR7
AFB1-C6-monoaldehyde
AFB1-C8-monoaldehyde
AFB1-dialcohol
CYP3A
AFQ1
protein adducts
1-Nitroso-naphthalene
N-Hydroxy-1-aminonaphthalene
1-Naphthylamine
1-Nitronaphthalene
1-Nitronaphthalene-5,6-oxide
1-Nitro-5-OH-6-glutathionyl-5,6-dihydronaphthalene
1-Nitro-5-glutathionyl-6-OH-5,6-dihydronaphthalene
1,2-Dihydro-5-nitro-1,2-naphthalenediol
1-Nitronaphthalene-7,8-oxide
1-Nitro-7-glutathionyl-8-OH-7,8-dihydronaphthalene
1-Nitro-7-OH-8-glutathionyl-7,8-dihydronaphthalene
Trichloroethylene
TCE oxide
Dichloroacetyl chloride
Dichloroacetic acid
1.2.1.5
Trichloroacetic acid
Chloral
Chloral hydrate
1.1.1.1
Trichloroethanol
Trichloroethanol-glucuronide
S-(1,2-Dichlorovinyl)glutathione
1,1-Dichloro-ethylene
2,2-Dichloro-1,1-ethanediol
2,2-Dichloro-acetaldehyde
S-(2,2-Dichloro-1-hydroxy)-ethyl glutathione
DCE epoxide
2-(S-Glutathionyl)-acetyl chloride
2-S-Glutathionyl acetate
Chloroacetyl chloride
S-(2-Chloroacetyl)-glutathione
2-(S-Glutathionyl)-acetyl glutathione
Chloroacetic acid
Bromobenzene
Bromobenzene-3,4-oxide
Bromobenzene-2,3-oxide
3,4-Dihydro-3-OH-4-S-glutathionyl bromobenzene
4-Bromophenol
CYP2E
4-Bromophenol-2,3-epoxide
4-Bromocatechol
4-Bromo-1,2-benzoquinone
Bromobenzene-3,4-dihydrodiol
Bromobenzene-2,3-dihydrodiol
2-Bromophenol
2,3-Dihydro-2-S-glutathionyl-3-hydroxy bromobenzene
1,2-Dibromoethane
2-Bromo-acetaldehyde
S-(Formylmethyl)-glutathione
Thiodiacetic acid
Thiodiacetic acid sulfoxide
Glutathione-episulfonium ion
S-(2-Hydroxyethyl)-glutathione
S-(2-Hydroxyethyl)-N-acetyl-L-cysteine
S-[2-(N7-Guanyl)ethyl]-N-acetyl-L-cysteine
00980 12/1/11
(c) Kanehisa Laboratories

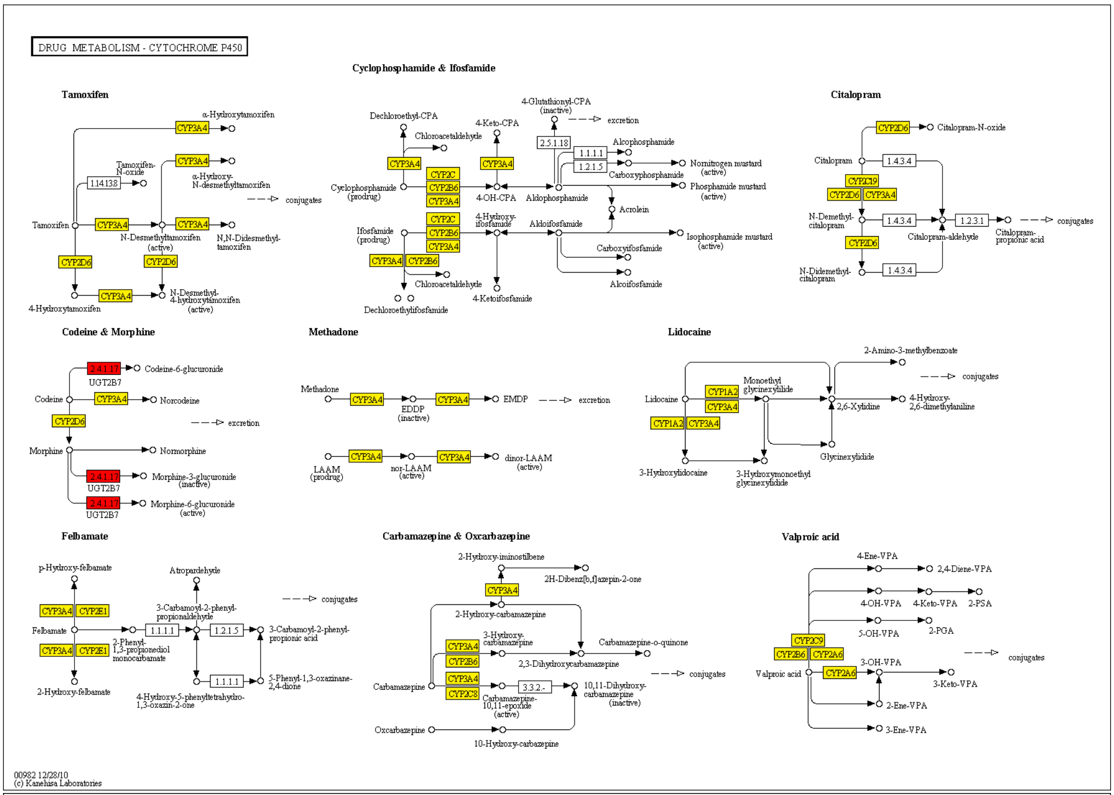
DRUG METABOLISM - CYTOCHROME P450
Cyclophosphamide & Ifosfamide
Tamoxifen
α-Hydroxytamoxifen
Tamoxifen-N-oxide
α-Hydroxy-N-desmethyltamoxifen
1.14.13.8
conjugates
N-Desmethyltamoxifen (active)
N,N-Didesmethyl-tamoxifen
4-Hydroxytamoxifen
N-Desmethyl-4-hydroxytamoxifen (active)
CYP3A4
CYP2D6
4-Glutathionyl-CPA (inactive)
excretion
Dechloroethyl-CPA
Chloroacetaldehyde
4-Keto-CPA
2.5.1.18
1.1.1.1
1.2.1.5
Alcophosphamide
Carboxyphosphamide
Nornitrogen mustard (active)
Phosphamide mustard (active)
Cyclophosphamide (prodrug)
CYP2C
CYP2B6
4-OH-CPA
Aldophosphamide
Acrolein
4-Hydroxy-ifosfamide
Aldoifosfamide
Isophosphamide mustard (active)
Ifosfamide (prodrug)
Carboxyifosfamide
Alcoifosfamide
Chloroacetaldehyde
4-Ketoifosfamide
Dechloroethylifosfamide
Citalopram
Citalopram-N-oxide
1.4.3.4
CYP2C19
N-Demethyl-citalopram
1.2.3.1
Citalopram-aldehyde
Citalopram-propionic acid
N-Didemethyl-citalopram
Codeine & Morphine
2.4.1.17
UGT2B7
Codeine-6-glucuronide
Codeine
Norcodeine
excretion
Morphine
Normorphine
Morphine-3-glucuronide (inactive)
Morphine-6-glucuronide (active)
Methadone
EDDP (inactive)
EMDP
LAAM (prodrug)
nor-LAAM (active)
dinor-LAAM (active)
Lidocaine
2-Amino-3-methylbenzoate
Monoethyl glycinexylidide
CYP1A2
2,6-Xylidine
4-Hydroxy-2,6-dimethylaniline
Glycinexylidide
3-Hydroxylidocaine
3-Hydroxymonoethyl glycinexylidide
Felbamate
p-Hydroxy-felbamate
Atropaldehyde
CYP2E1
3-Carbamoyl-2-phenyl-propionaldehyde
3-Carbamoyl-2-phenyl-propionic acid
2-Phenyl-1,3-propanediol monocarbamate
2-Hydroxy-felbamate
4-Hydroxy-5-phenyltetrahydro-1,3-oxazin-2-one
5-Phenyl-1,3-oxazinane-2,4-dione
Carbamazepine & Oxcarbazepine
2-Hydroxy-iminostilbene
2H-Dibenz[b,f]azepin-2-one
2-Hydroxy-carbamazepine
3-Hydroxy-carbamazepine
Carbamazepine-o-quinone
2,3-Dihydroxycarbamazepine
CYP2C8
3.3.2.-
Carbamazepine
Carbamazepine-10,11-epoxide (active)
10,11-Dihydroxy-carbamazepine (inactive)
Oxcarbazepine
10-Hydroxy-carbazepine
Valproic acid
4-Ene-VPA
2,4-Diene-VPA
4-OH-VPA
4-Keto-VPA
2-PSA
5-OH-VPA
2-PGA
CYP2C9
CYP2A6
3-OH-VPA
3-Keto-VPA
2-Ene-VPA
3-Ene-VPA
00982 12/28/10
(c) Kanehisa Laboratories

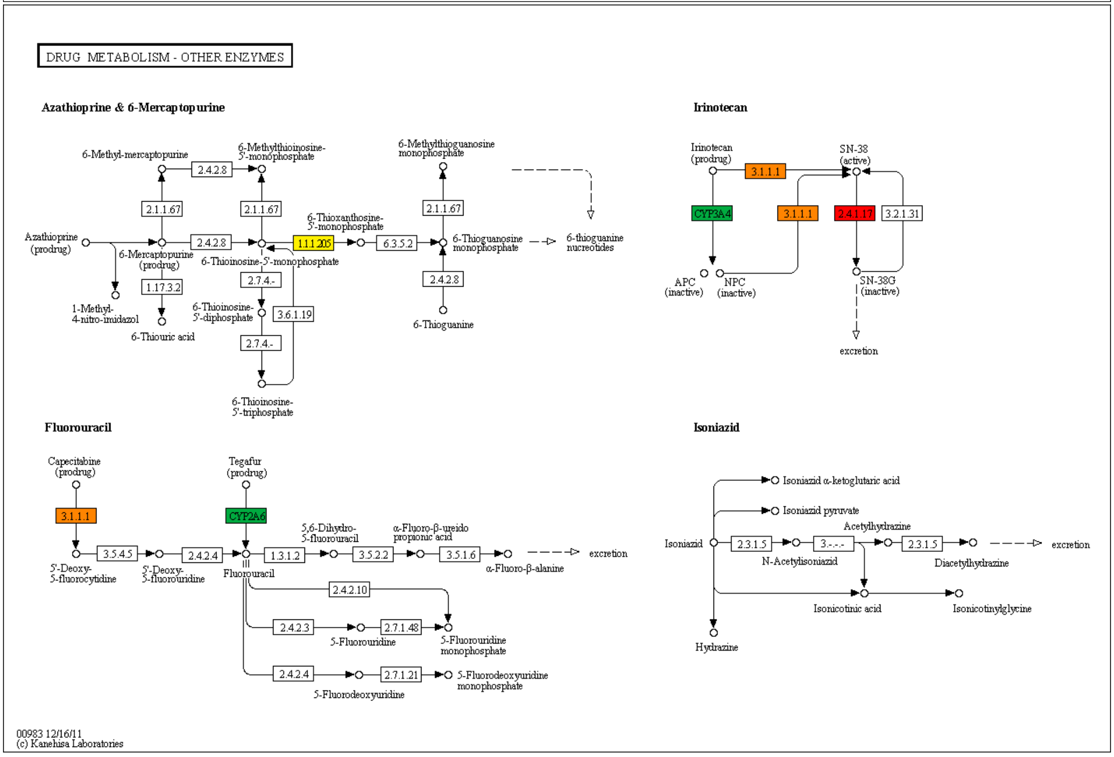
DRUG METABOLISM - OTHER ENZYMES
Azathioprine & 6-Mercaptopurine
6-Methyl-mercaptopurine
6-Methylthioinosine-5'-monophosphate
6-Methylthioguanosine monophosphate
2.4.2.8
2.1.1.67
Azathioprine (prodrug)
6-Mercaptopurine (prodrug)
6-Thioxanthosine-5'-monophosphate
1.1.1.205
6.3.5.2
6-Thioguanosine monophosphate
6-thioguanine nucleotides
6-Thioinosine-5'-monophosphate
2.7.4.-
1.17.3.2
1-Methyl-4-nitro-imidazol
6-Thiouric acid
6-Thioinosine-5'-diphosphate
3.6.1.19
6-Thioguanine
6-Thioinosine-5'-triphosphate
Irinotecan
Irinotecan (prodrug)
3.1.1.1
SN-38 (active)
CYP3A4
2.4.1.17
3.2.1.31
APC (inactive)
NPC (inactive)
SN-38G (inactive)
excretion
Fluorouracil
Capecitabine (prodrug)
Tegafur (prodrug)
CYP2A6
3.5.4.5
2.4.2.4
5'-Deoxy-5-fluorocytidine
5'-Deoxy-5-fluorouridine
Fluorouracil
1.3.1.2
5,6-Dihydro-5-fluorouracil
3.5.2.2
α-Fluoro-β-ureido propionic acid
3.5.1.6
α-Fluoro-β-alanine
excretion
2.4.2.10
2.4.2.3
5-Fluorouridine
2.7.1.48
5-Fluorouridine monophosphate
2.7.1.21
5-Fluorodeoxyuridine
5-Fluorodeoxyuridine monophosphate
Isoniazid
Isoniazid α-ketoglutaric acid
Isoniazid pyruvate
Acetylhydrazine
2.3.1.5
3.-.-.-
N-Acetylisoniazid
Diacetylhydrazine
excretion
Isonicotinic acid
Isonicotinylglycine
Hydrazine
00983 12/16/11
(c) Kanehisa Laboratories

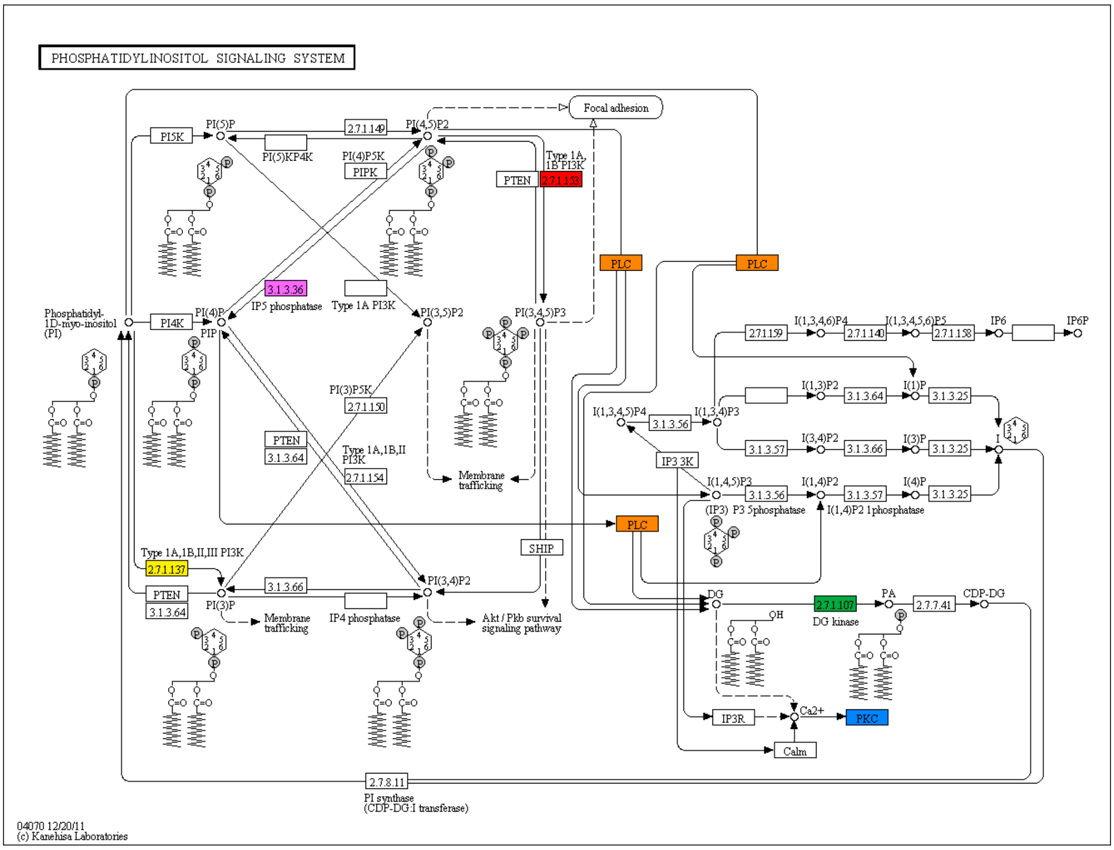
PHOSPHATIDYLINOSITOL SIGNALING SYSTEM
Focal adhesion
PI5K
PI(5)P
2.7.1.149
PI(4,5)P2
PI(5)KP4K
PI(4)P5K
PIPK
Type 1A, 1B PI3K
PTEN
2.7.1.153
PLC
Phosphatidyl-1D-myo-inositol (PI)
PI4K
PI(4)P
PIP
3.1.3.36
IP5 phosphatase
Type 1A PI3K
PI(3,5)P2
PI(3,4,5)P3
PI(3)P5K
2.7.1.150
PTEN
3.1.3.64
Type 1A,1B,II PI3K
2.7.1.154
Membrane trafficking
Type 1A,1B,II,III PI3K
2.7.1.137
3.1.3.66
PI(3,4)P2
PI(3)P
IP4 phosphatase
Akt / Pkb survival signaling pathway
SHIP
I(1,3,4,6)P4
I(1,3,4,5,6)P5
IP6
IP6P
2.7.1.159
2.7.1.140
2.7.1.158
I(1,3)P2
I(1)P
I(1,3,4,5)P4
3.1.3.56
I(1,3,4)P3
3.1.3.25
I(3,4)P2
I(3)P
3.1.3.57
IP3 3K
I(1,4,5)P3
I(1,4)P2
I(4)P
(IP3) P3 5phosphatase
I(1,4)P2 1phosphatase
I
DG
2.7.1.107
DG kinase
PA
2.7.7.41
CDP-DG
IP3R
Ca2+
PKC
Calm
2.7.8.11
PI synthase (CDP-DG:I transferase)
04070 12/20/11
(c) Kanehisa Laboratories

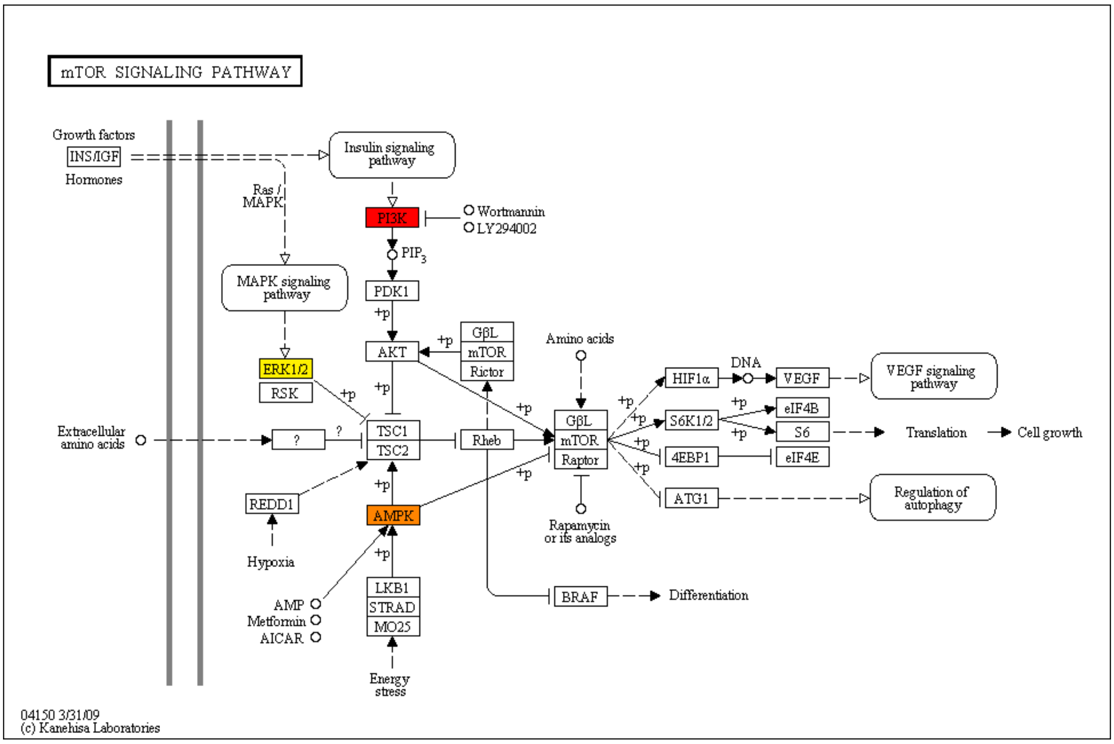
mTOR SIGNALING PATHWAY
Growth factors
INS/IGF
Hormones
Insulin signaling pathway
Ras / MAPK
PI3K
Wortmannin
LY294002
PIP3
MAPK signaling pathway
PDK1
+p
GβL
mTOR
Rictor
AKT
ERK1/2
RSK
Amino acids
Extracellular amino acids
?
TSC1
TSC2
Rheb
GβL
mTOR
Raptor
HIF1α
DNA
VEGF
VEGF signaling pathway
S6K1/2
eIF4B
S6
Translation
Cell growth
4EBP1
eIF4E
ATG1
Regulation of autophagy
REDD1
Hypoxia
AMPK
Rapamycin or its analogs
AMP
Metformin
AICAR
LKB1
STRAD
MO25
Energy stress
BRAF
Differentiation
04150 3/31/09
(c) Kanehisa Laboratories
